# Factors to preserve CpG-rich sequences in methylated CpG islands

**DOI:** 10.1186/s12864-015-1286-x

**Published:** 2015-02-28

**Authors:** Hiroki Miyahara, Osamu Hirose, Kenji Satou, Yoichi Yamada

**Affiliations:** Division of Electrical and Computer Engineering, Graduate School of Natural Science and Technology, Kanazawa University, 920-1192 Kanazawa, Japan; Faculty of Electrical and Computer Engineering, Institute of Science and Engineering, Kanazawa University, 920-1192 Kanazawa, Japan

**Keywords:** CpG island, DNA methylation, CpG selection, CpG fixation, Biased gene conversion

## Abstract

**Background:**

Mammalian CpG islands (CGIs) normally escape DNA methylation in all adult tissues and developmental stages. However, in our previous study we unexpectedly identified many methylated CGIs in human peripheral blood leukocytes. Methylated CpG dinucleotides convert to TpG dinucleotides through deaminization of their cytosine bases more frequently than hypomethylated CpG dinucleotides. Therefore, we wondered how methylated CGIs in germline or non-germline cells maintain their CpG-rich sequences. It is known that events such as germline hypomethylation, CpG selection, biased gene conversion (BGC), and frequent CpG fixation can contribute to the maintenance of CpG-rich sequences in methylated CGIs in germline or non-germline cells. However, it has not been investigated which of the processes maintain CpG-rich sequences of methylated CGIs in each genomic position.

**Results:**

In this study, we comprehensively examined the contribution of the processes described above to the maintenance of CpG-rich sequences in methylated CGIs in germline and non-germline cells which were classified by genomic positions. Approximately 60–80% of CGIs with high methylation in H1 cell line (H1-HM) in all the genomic positions showed a low average CpG → TpG/CpA substitution rate. In contrast, fewer than half the numbers of CGIs with H1-HM in all the genomic positions showed a low average CpG → TpG/CpA substitution rate and low levels of methylation in sperm cells (SPM-LM). Furthermore, a small fraction of CGIs with a low average CpG → TpG/CpA substitution rate and high levels of methylation in sperm cells (SPM-HM) showed CpG selection.

On the other hand, independent of the positions in genes, most CGIs with SPM-HM showed a slightly higher average TpG/CpA → CpG substitution rate compared with those with SPM-LM.

**Conclusions:**

Relatively high numbers (approximately 60–80%) of CGIs with H1-HM in all the genomic positions preserve their CpG-rich sequences by a low CpG → TpG/CpA substitution rate caused mainly by their SPM-LM, and for those with SPM-HM partly by CpG selection and TpG/CpA → CpG fixation. BGC has little contribution to the maintenance of CpG-rich sequences of CGIs with SPM-HM which were classified by genomic positions.

**Electronic supplementary material:**

The online version of this article (doi:10.1186/s12864-015-1286-x) contains supplementary material, which is available to authorized users.

## Background

Mammalian DNA methylation occurs at the 5-position of the cytosine of CpG dinucleotides and is catalyzed by DNA methyltransferases (DNMT1, DNMT3A, DNMT3B) [1,2]. DNA methylation contributes to spatial and temporal gene regulation [3], inactivation of transposable elements [4], genomic stabilization [5], X chromosome inactivation in females [6], and genomic imprinting [7]. Aberrant DNA methylation leads to various types of cancer [8,9], and the knockout of DNA methyltransferases in mice results in embryonic and postnatal lethality [1,2].

Methylated CpG dinucleotides convert to TpG dinucleotides through deaminization of their cytosine bases more frequently than hypomethylated CpG dinucleotides [10]. This leads to G/T mismatches in double-stranded DNA, resulting in transition from G/T to A/T base pair by incomplete DNA repair systems [10]. This CpG → TpG (or CpA in the complementary strand) transition in germline cells often leads to genetic mutations, resulting in genetic disorders [11]. Since most of CpG dinucleotides in mammalian genomes are subject to DNA methylation, only ~25% of the number of CpG dinucleotides expected from the GC-content in mammalian genomes are present [12]. The exception is CpG islands (CGIs) that lie in the promoter regions of house-keeping and a proportion of tissue-specific genes [13,14]. CGIs are rich in CpG dinucleotides because they are generally subject to low levels of DNA methylation (LM) in all developmental stages and adult tissues [15,16]. However, a fraction of CGIs are subjected to a parent-of-origin dependent allele-specific [17,18] and random-mono-allelic methylation in imprinted genes and X-chromosomes [6], respectively.

Contrary to these common beliefs, we previously found a certain number of CGIs that are biallelically methylated in adult human peripheral blood leucocytes [19,20]. As a result, we were confronted with the question of how CGIs with high methylation (HM) in somatic cells (or germline cells) can maintain their CpG rich sequences because methylated CGIs have more a rapid CpG → TpG/CpA substitution.

The loss of CGIs is also a common event in mammalian genomes [21], although some countermeasures have been suggested that suppress an extreme decrease of CpG dinucleotides on methylated CGIs in germline or non-germline cells. 1) Methylated CGIs in non-germline cells are hypomethylated in germline cells, resulting in suppression of CpG → TpG/CpA substitution because base substitution in non-germline cells is not transmitted from parent to progeny. 2) CpG selection preserves CpG density on methylated CGIs in germline cells [22]. 3) BGC occurring within recombination hotspots leads to rapid CpG gain in methylated CGIs in germline cells [23]. 4) TpG/CpA → CpG substitution often occurs at newly generated CpG → TpG/CpA substitution sites [24]. However, it has not been investigated which of the countermeasures maintain CpG-rich sequences of methylated CGIs in each genomic position.

On the other hand, recent comprehensive methylome analyses at single-base resolution have revealed whole genome methylation statuses in some human tissues. These pioneering studies were based on bisulfite conversion in human H1 embryonic stem cells, IMR90 fetal lung fibroblasts, and mature sperm cells [25]. In addition, the numerous orthology identification projects between primates have been developed, allowing one to calculate evolutionary base substitution rates [26].

In this study, using these genome-wide datasets, we thus classified human CGIs into four categories according to their locations in genes and comprehensively examined the contribution of the countermeasures listed above that limit extreme reduction in the number of CpG dinucleotides in the methylated CGIs in H1, IMR90 and mature sperm cells.

## Methods

### Classification of CGIs based on their locations in genes

Human genome sequence (hg18) and CGI sequences (CpgIslandExt.txt from hg18) were downloaded from the UCSC database [http://genome.ucsc.edu/]. The CpgIslandExt contains 28,226 of CGIs that are computationally annotated. Known gene sequences (knownGene.txt from hg18) and conversion text from UCSC ID to Gene symbol (kgXref.txt from hg18) were also downloaded from the UCSC database [http://genome.ucsc.edu/]. The Known Genes table included 66,803 transcripts (53,036 coding transcripts and 13,767 noncoding transcripts). Using these files, we classified the CGIs into four categories (5′CGI, 3′CGI, intragenic CGI, and intergenic CGI) according to their locations in genes as follows: (1) 5′CGIs that overlap with a region from 3 kb upstream of the first exon to the first exon; (2) 3′CGIs that overlap with a region between the final exon and 3 kb downstream; (3) intragenic CGIs overlaps with a region between the first intron of any gene and its final intron; (4) intergenic CGIs that overlap with any genomic region excluding those defined by (1), (2), and (3). When a CGI belongs to several classes, it was classified into one of the four classes according to the following priority: (1) → (2) → (3) → (4). In addition, if a CGI classified according to the categories above was associated with a protein-coding gene, it was defined as a coding CGI. The other CGIs were defined as non-coding CGIs. In the Known Genes table, transcripts that had the same $cdsStart and $cdsEnd values were defined as non-coding transcripts.

### Calculation of DNA methylation and hydroxymethylation levels on a CGI

**Figure 1:**
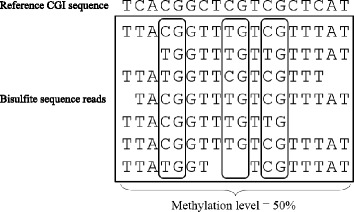
**Calculation of CGI methylation level.** Reference CGI sequence indicates the CGI sequence prior to bisulfite conversion. Sequences within the square show bisulfite sequence reads mapped to the reference CGI sequence.

We downloaded whole-genome DNA methylation data at single-base resolution from two types of human cell lines (H1 and IMR90) from the human DNA methylome website [http://neomorph.salk.edu/human_methylome/] [25]. As shown in Figure 1, for H1 and IMR90 cell lines, we calculated DNA methylation level on each CGI according to the following formula:

$$ \mathrm{D}\mathrm{N}\mathrm{A}\ \mathrm{Methylation}\ \mathrm{Level}\ \left(\%\right) = 100 \times \mathrm{S}\ /\ \left(\mathrm{S}+\mathrm{C}\right) $$

in which S is the total number of methylated cytosines (i.e., CG) on bisulfite sequence reads mapped to each CGI, and C denotes the total number of hypomethylated cytosines (i.e., TG or CA). Methylation level was calculated on both two DNA strands, and the average methylation level of the two strands was calculated as the methylation level of a CGI.

Reduced representation bisulfite sequencing (RRBS) DNA methylation data from mature human sperm cells were downloaded from Gene Expression Omnibus (GEO accession numbers: GSM1396533, GSM1396534). For the RRBS data from mature sperm cells, DNA methylation level on each CGI was calculated according to the following formula:

$$ \mathrm{D}\mathrm{N}\mathrm{A}\ \mathrm{methylation}\ \mathrm{level}\ \left(\%\right) = 100\times \mathrm{S}\ /\ \left(\mathrm{S}+\mathrm{C}\right) $$

in which S is the total number of methylated cytosines on bisulfite sequence reads mapped to each CGI, and C denotes the total number of hypomethylated cytosines.

Whole-genome 5-hydroxymethylcytosine (5hmC) data at single-base resolution from H1 cell line was downloaded from Gene Expression Omnibus (GEO accession number: GSM882245). 5hmC level on each CGI was calculated according to the following formula:

$$ 5\mathrm{h}\mathrm{m}\mathrm{C}\ \mathrm{level}\ \left(\%\right) = 100 \times \mathrm{H}\mathrm{C}\ /\ \left(\mathrm{H}\mathrm{C}+\mathrm{N}\mathrm{H}\mathrm{C}\right) $$

in which HC is the total number of hydroxymethylated cytosines on bisulfite sequence reads mapped to each CGI, and NHC denotes the total number of hypo-hydroxymethylated cytosines.

In calculation of DNA methylation and 5hmC levels above, when there were fewer than five methylated or hypomethylated cytosines over all reads mapped to all CpG dinucleotides in a CGI, the CGI was excluded from further analysis.

We also downloaded whole-genome DNA methylation data at single-base resolution from mature human mature sperm cells (BCMsample25783, BCMsample25784, and Molaro_pooled) from the Bioinformatics Research Laboratory, Baylor College of Medicine website [http://genboree.org/EdaccData/Genomic_Human_Germline_Hypomethylation/Sperm_Bisulfite-Seq_Data/] [27,28]. For the mature human sperm cell samples, the average of DNA methylation levels on all CpG sites in a CGI was calculated as DNA methylation level on the CGI.

RRBS DNA methylation data from human H1 cell line was downloaded from Gene Expression Omnibus (GEO accession numbers: GSM621705). In the GSM621705 from H1 cell line, methylation proportions are calculated as (methylated calls/(methylated calls + unmethylated calls)) for all CpG sites covered by at least 4 reads. For the GSM621705, we calculated the average of DNA methylation levels on informative all CpG sites in a CGI, and considered it as DNA methylation level on the CGI.

### Calculation of base substitution rates for CGIs: CpG → TpG/CpA, TpG/CpA → CpG, CpG → GpG/ApG/CpC/CpT, GpG/ApG/CpC/CpT → CpG, A/T → G/C, and G/C → A/T substitution rates

To calculate base substitution rates for each CGI, alignments of orthologous regions between human, chimpanzee, and their ancestral sequences were used. The alignments (release 51 epo 9 eutherian) of orthologous regions among human, chimpanzee, and their ancestral sequence were downloaded from the ensemble database [ftp://ftp.ensembl.org/pub/release-51/emf/ensembl-compara/epo_9_eutherian/].

When the alignments covered less than a 50% region of a CGI, that CGI was removed from analysis. CpG → TpG/CpA, TpG/CpA → CpG, CpG → GpG/ApG/CpC/CpT, GpG/ApG/CpC/CpT → CpG, A/T → G/C, and G/C → A/T substitution rates for each CGI were calculated according to the following formula:

i). CpG → TpG/CpA substitution rate = the number of derived TpG/CpAs on given orthologous CGIs in human and chimpanzee/twice the number of their common ancestral CpGs.
ii). TpG/CpA → CpG substitution rate = the number of derived CpGs on given orthologous CGIs in human and chimpanzee/twice the number of their common ancestral TpG/CpAs.
iii). CpG → GpG/ApG/CpC/CpT substitution rate = the number of derived GpG/ApG/CpC/CpTs on given orthologous CGIs in human and chimpanzee/twice the number of their common ancestral CpGs.
iv). GpG/ApG/CpC/CpT → CpG substitution rate = the number of derived CpGs on given orthologous CGIs in human and chimpanzee/twice the number of their common ancestral GpG/ApG/CpC/CpTs.
v). A/T → G/C substitution rate = the number of derived G/Cs in a non-CpG context on given orthologous CGIs in human and chimpanzee/twice the number of their common ancestral A/Ts.
vi). G/C → A/T substitution rate = the number of derived A/Ts on given orthologous CGIs in human and chimpanzee/twice the number of their common ancestral G/Cs in a non-CpG context.

### Decision tree

We determined association rules between methylation levels in mature sperm cells and CpG → TpG/CpA substitution rates by using a decision tree. R package, mvpart and its default parameters were used. Distinctive class labels were low methylation (LM: methylation level ≤20%) and high methylation (HM: methylation level ≥80%) for CGIs.

### Clustering of gene expression data

The gene expression data [DataSet Record: GDS596] from 79 physiologically normal human tissues were downloaded from GEO [http://www.ncbi.nlm.nih.gov/geo/] [29]. The gene expression data were normalized using the Robust Multi-array Average (RMA) algorithm. Clustering was performed using Cluster3.0 [http://bonsai.hgc.jp/~mdehoon/software/cluster/software.htm].

## Results and discussion

### Identification of highly methylated CGIs in H1 and IMR90 cells

To identify highly methylated CGIs in non-germline cells, we used whole-genome DNA methylation data at single base-pair resolution from two human cell lines (H1 and IMR90) [25]. Hereafter, we refer to this whole-genome bisulfite sequencing as WGBS. H1 cell line is from inner cell masses in blastocyst stage by which erasure and *de novo* re-establishment of global DNA methylation occurs. Consequently, DNA methylation attains its somatic level by the blastocyst stage. In addition, since IMR90 is a diploid fibroblast-like cell line and established from lung tissue of a female fetus, its DNA methylation is also a somatic level. We therefore expected that these cells have many highly methylated CGIs, and used them to identify highly methylated CGIs. The numbers of CGIs in which five or more methylated/hypomethylated CpG sites were mapped from bisulfite sequence reads were 22,353 and 22,357 in H1 and IMR90, respectively. Table 1 shows the numbers of CGIs with high (≥80%) and low (≤20%) methylation in the relative positions in a gene. Most (~90%) of the 5′CGIs showed LM in both cell types, hence ~10% of 5′CGIs were highly methylated. In contrast, relatively high numbers of 3′CGIs (~65%), intragenic CGIs (~70%), and intergenic CGIs (30–50%) were subject to high methylation in both cell types. A total of 6,257 and 4,615 highly methylated CGIs were identified in H1 and IMR90 cells, respectively.

**Table 1:** **Number of CGIs in each genomic position showing HM and LM in H1 and IMR90 cells**

		**H1**		**IMR90**	
		**LM (≤20%)**	**HM (≥80%)**	**LM (≤20%)**	**HM (≥80%)**
5'CGI	coding	10733	985	10762	677
	non-coding	355	224	358	163
3'CGI	coding	455	1047	459	894
	non-coding	61	74	67	52
Intragenic CGI	coding	862	2481	873	2045
	non-coding	98	79	93	53
Intergenic CGI	non-coding	1505	1367	1643	731
Total		14069	6257	14255	4615

Coding indicates CGIs that associate with a protein-coding gene. Non-coding indicates CGIs that associate with a non-protein-coding gene. LM shows CGIs with methylation of ≤20%. HM shows CGIs with methylation of ≥80%.

### Comparison of DNA methylation status of CGIs in non-germline and sperm cells

To compare DNA methylation status between non-germline (H1 and IMR90) and mature sperm cells, the average DNA methylation level of each CGI was calculated using whole-genome DNA methylation data from mature human sperm cells at single base-pair resolution [27,28]. Table 2 shows the comparison of CGI DNA methylation statuses in H1 and sperm cells. Independent of coding/non-coding and positions in a gene, most of CGIs with LM in H1 cells (denoted H1-LM) showed LM also in sperm cells. This corresponds to previous reports where most CGIs are hypomethylated in all developmental stages and adult tissues. More than half the numbers of coding 5′CGIs with H1-HM (denoted as H1-HM-coding-5′CGIs), non-coding intragenic CGIs with H1-HM, and intergenic CGIs with H1-HM were lowly methylated in sperm cells, suggesting that their CpG rich sequences are maintained mainly by their LM in germline cells. Moreover, 20–45% of the other CGIs with H1-HM also showed SPM-LM. This suggests that many of the other CGIs with H1-HM also preserve their CpG-rich sequences by their LM in sperm cells. These tendencies were also observed in IMR90 cells (data not shown).

**Table 2:** **Comparison of CGI methylation between H1 and sperm cells**

					**Sperm methylation**	
**CGI**	**HI methylation**	**Gene**	**CGI number**	**Sperm sample**	**LM**	**HM**
5'CGI	HM	coding	985	BCMsample 25783	531	395
				BCMsample 25784	534	395
				Molaro_metylation	523	384
		non- coding	224	BCMsample 25783	102	100
				BCMsample 25784	102	105
				Molaro_metylation	105	96
	LM	coding	10733	BCMsample 25783	10693	6
				BCMsample 25784	10683	7
				Molaro_metylation	10721	5
		non- coding	355	BCMsample 25783	349	0
				BCMsample 25784	350	0
				Molaro_metylation	355	0
3′CGI	HM	coding	1047	BCMsample 25783	358	622
				BCMsample 25784	348	617
				Molaro_metylation	337	613
		non-coding	74	BCMsample 25783	30	41
				BCMsample 25784	26	40
				Molaro_metylation	24	37
	LM	coding	455	BCMsample 25783	445	3
				BCMsample 25784	411	4
				Molaro_metylation	448	3
		non coding	61	BCMsample 25783	61	0
				BCMsample 25784	60	0
				Molaro_metylation	61	0
Intragenic CGI	HM	coding	2481	BCMsample 25783	581	1706
				BCMsample 25784	556	1727
				Molaro_metylation	559	1628
		non-coding	79	BCMsample 25783	48	22
				BCMsample 25784	49	26
				Molaro_metylation	52	20
	LM	coding	862	BCMsample 25783	848	7
				BCMsample 25784	845	6
				Molaro_metylation	851	3
		non-coding	98	BCMsample 25783	98	0
				BCMsample 25784	95	0
				Molaro_metylation	98	0
Intergenic CGI	HM	-	1367	BCMsample 25783	805	422
				BCMsample 25784	800	439
				Molaro_metylation	815	379
	LM	-	1505	BCMsample 25783	1480	4
				BCMsample 25784	1471	5
				Molaro_metylation	1492	0

Coding indicates CGIs that overlap with a protein-coding gene. Non-coding indicates CGIs that overlap with a non-protein-coding gene. LM shows CGIs with ≤20% of methylation. HM shows CGIs with ≥80% methylation. Molaro_pooled, BCMsample25784, and BCMsample25783 describe separate human sperm samples.

### Contribution of SPM-LM and CpG selection in the maintenance of CpG-rich sequences in methylated CGIs.

Independent of the CGI positions within a gene, we found many highly methylated CGIs in H1 and IMR90 cells. We then addressed the question of how these CGIs maintain their CpG rich sequences because methylated CpG dinucleotides convert to TpG dinucleotides more frequently than hypomethylated CpG dinucleotides.

**Figure 2:**
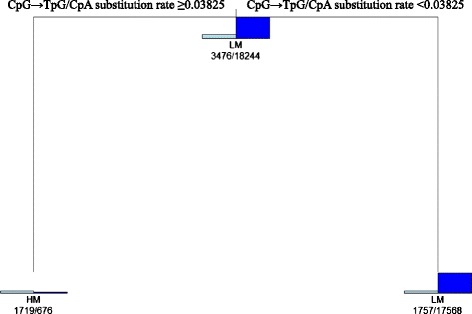
**Determination of a threshold for the CpG → TpG/CpA substitution rate by a decision tree.** CGIs with a methylation level of ≥80% in human sperm cells are depicted by a light blue box labeled HM, and CGIs with a methylation level of ≤20% in human sperm cells are indicated by a the blue box and labeled as LM. In each node of the tree, either label (HM or LM) is assigned to the greater number of CGIs as described under the two boxes (light blue and blue). CpG → TpG/CpA substitution rate ≥0.03825 shows a CpG → TpG/CpA substitution rate of ≥0.03825. CpG → TpG/CpA substitution rate <0.03825 denotes a CpG → TpG/CpA substitution rate of <0.03825.

We first examined the possibility that methylated CGIs in H1 and IMR90 cells are lowly methylated in sperm cells, resulting in a lower CpG → TpG/CpA substitution rate compared with highly methylated CGIs in sperm cells. Here, a threshold was needed to infer whether or not CGIs have a low CpG → TpG/CpA substitution rate, and to determine this a decision tree was applied. High and low levels of methylation on CGIs in sperm cells were used as distinctive class labels (HM: high methylation; LM: low methylation) of CGIs in the decision tree. The decision tree can find an optimal threshold for the CpG → TpG/CpA substitution rate that split CGIs into either one of two class labels in each leaf (Figure 2).

Figure 2 shows the decision tree for the CpG → TpG/CpA substitution rate. Of 21,720 CGIs used in the decision tree, 3,476 and 18,244 have HM (≥80%) and LM (≤20%) in sperm cells, respectively (Figure 2). Of the 21,720 CGIs, 2,395 CGIs (HM: 1,719; LM: 676) showed a CpG → TpG/CpA substitution rate of ≥0.03825, whereas 19,325 (HM: 1,757; LM: 17,568) showed a CpG → TpG/CpA substitution rate of <0.03825 (Figure 2). Accordingly, this means that a threshold of 0.03825 for the CpG → TpG/CpA substitution rate achieved the highest ratio of HM to total CGIs in the left leaf (1,719/2,395; ~70% for CGIs with a CpG → TpG/CpA substitution rate of ≥0.03825) and the highest ratio of LM to total CGIs in the right leaf (17,568/19,325; ~90% for CGIs with a CpG → TpG/CpA substitution rate of <0.03825).

The number of CGIs with a CpG → TpG/CpA substitution rate of <0.03825 and their sperm-methylation status are described in Additional file 1: Table S1. 78% (770/985) of H1-HM-coding-5′CGIs and 72% (761/1047) of coding 3′CGIs with H1-HM showed a CpG → TpG/CpA substitution rate of <0.03825, whereas 56–67% of the other CGIs with H1-HM showed a CpG → TpG/CpA substitution rate of <0.03825 (Additional file 1: Table S1). These results suggest that approximately 60–80% of CGIs with H1-HM in all the genomic positons maintain their CpG-rich sequences by a low CpG → TpG/CpA substitution rate. On the other hand, about half the numbers of H1-HM-coding-5′CGIs, non-coding intragenic CGIs with H1-HM, and intergenic CGIs with H1-HM showed a CpG → TpG/CpA substitution rate of <0.03825 and SPM-LM (Additional file 1: Table S1). In contrast, fewer than half the numbers (approximately 20–40%) of the other CGIs with H1-HM showed a CpG → TpG/CpA substitution rate of <0.03825 and SPM-LM (Additional file 1: Table S1). These results suggest that approximately 60–80% of CGIs with H1-HM in all the genomic positons maintain their CpG-rich sequences by a low CpG → TpG/CpA substitution rate caused not only by their SPM-LM but also by another mechanism, such as CpG selection (Additional file 1: Table S1).

In addition, to confirm the results obtained from the WGBS data, we also conducted the same analysis as described above using RRBS data from human H1 cell lines and mature sperm cells instead of the WGBS data. Here, to infer whether or not CGIs have a low CpG → TpG/CpA substitution rate, a CpG → TpG/CpA substitution rate of 0.03984 was used as a threshold. Since the RRBS data lacked DNA methylation data sufficient to calculate DNA methylation levels in more CGIs compared with the WGBS data, we were not able to identity DNA methylation levels in most CGIs especially in mature sperm cells. However, apart from that, tendencies similar to the above were observed also in results obtained from the RRBS data (Additional file 2: Table S2).

We next examined the contribution of CpG selection to a low CpG → TpG/CpA substitution rate in CGIs with SPM-HM. Since CpG selection but not hypo-deamination leads to not only a low CpG → TpG/CpA substitution rate but also a low CpG → GpG/ApG/CpC/CpT substitution rate, examining the CpG → GpG/ApG/CpC/CpT substitution rate can distinguish between low CpG → TpG/CpA substitution rates by CpG selection and by hypo-deamination.

**Figure 3:**
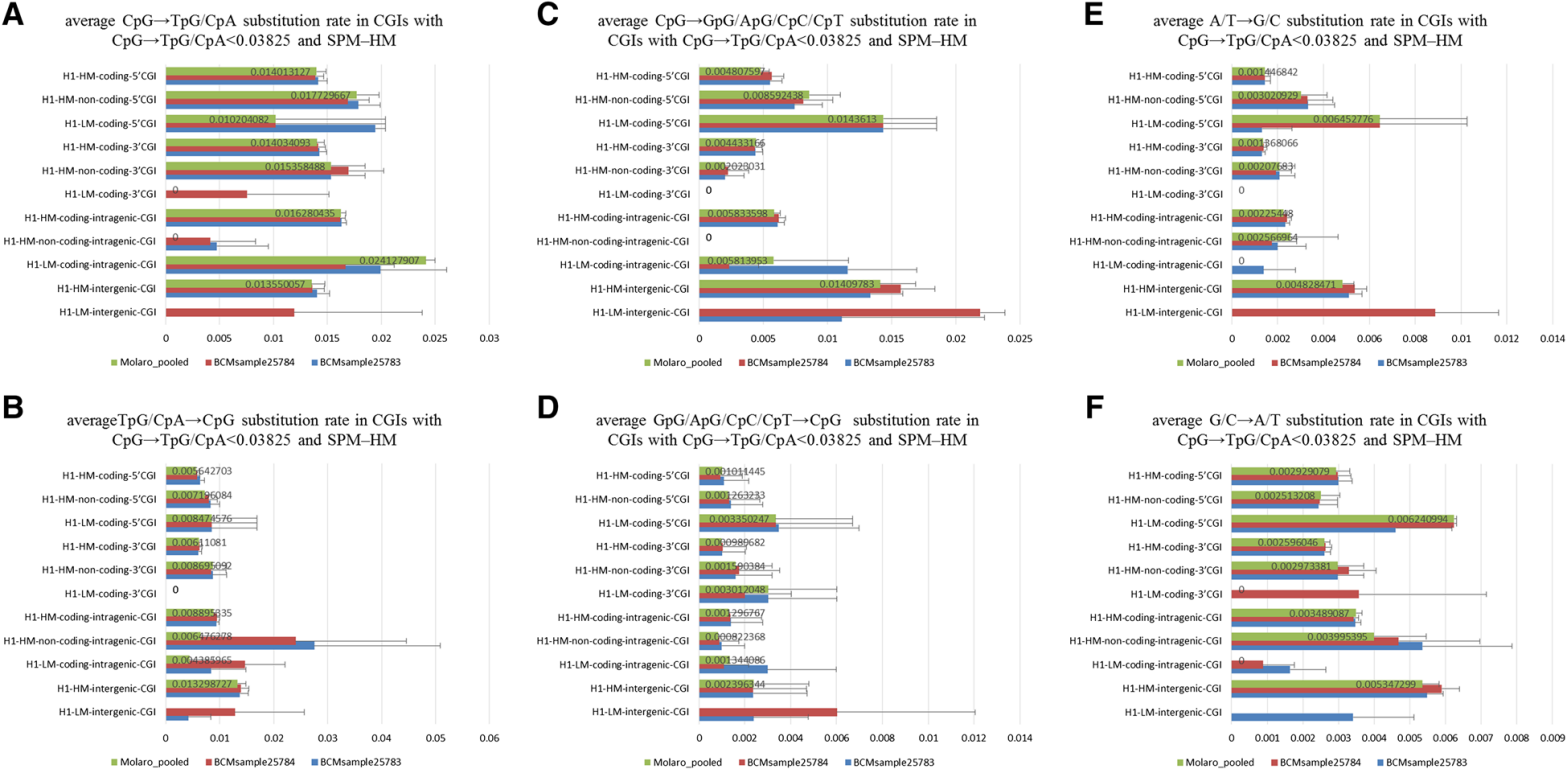
**Base substitution rate in sperm methylated CGIs with CpG → TpG/CpA < 0.03825. A**, **B**, **C**, **D**, **E**, and **F** show the average CpG → TpG/CpA, TpG/CpA → CpG, CpG → GpG/ApG/CpC/CpT, GpG/ApG/CpC/CpT → CpG, A/T → G/C, and G/C → A/T substitution rates in CGIs with a CpG → TpG/CpA substitution rate of <0.03825 and SPM-HM, respectively. The prefixes of H1-LM- and H1-HM- show low and high methylation of CGIs in H1 cells, respectively. Coding- and non-coding- indicate CGIs that associate with protein-coding and non-coding genes, respectively. Molaro_pooled, BCMsample25784, and BCMsample25783 describe separate human sperm samples. SPM-LM denotes CGIs with a low methylation of ≤20% in sperm cells. SPM-HM shows CGIs with a high methylation of ≥80% in sperm cells. Error bars represent standard errors. CpG → TpG/CpA < 0.03825 shows a CpG → TpG/CpA substitution rate of <0.03825.

Calculation of several base substitution rates involved in CpG selection, CpG fixation, and BGC showed that only H1-HM-non-coding-3′CGIs and H1-HM-non-coding-intragenic CGIs have CpG selection, because the average CpG → GpG/ApG/CpC/CpT substitution rate, but not the other average substitution rates, was extremely low in the above CGIs (Figure 3C and Additional file 1: Table S1). Note that although half of the H1-HM-non-coding-3′CGIs but none of the H1-HM-non-coding-intragenic CGIs overlap protein-coding exons, average TpG/CpA → CpG, GpG/ApG/CpC/CpT → CpG, A/T → G/C, and G/C →A/T substitution rates were not extremely low in these CGIs compared with the other CGIs (Figure 3). This indicates that base selection for protein-coding exons is not the cause of CpG selection for these CGIs. In addition, we did not find any imprinting control regions (ICRs) in these CGIs, which are often associated with imprinted genes and subject to CpG selection, suggesting that these CGIs may have important functions distinct from those of ICRs [30]. However, the cause of the low average CpG → TpG/CpA substitution rate in sperm methylated CGIs, other than in H1-HM-non-coding-3′CGIs and H1-HM-non-coding-intragenic CGIs, is unknown.

We also examined the contribution of CpG selection to a low CpG → TpG/CpA substitution rate in CGIs with SPM-HM using the RRBS data from H1 cell line and mature sperm cells instead of the WGBS data. As a result, we were not able to find CpG selection in any CGIs with a CpG → TpG/CpA substitution rate of <0.03984 and SPM-HM (Additional file 3: Figure S1A–S1B and Additional file 2: Table S2). This may result from much fewer numbers of H1-HM-non-coding-3′CGIs and H1-HM-non-coding-intragenic CGIs identified by the RRBS compared with those identified by the WGBS (Additional file 1: Table S1 and Additional file 2: Table S2).

**Figure 4:**
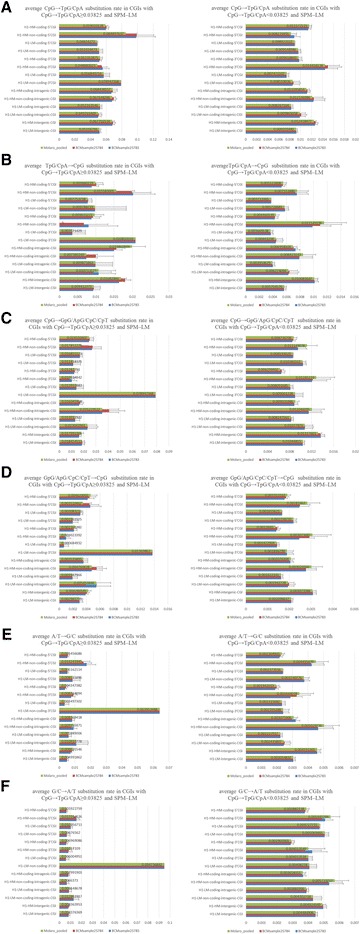
**Comparison of base substitution rates between lowly methylated CGIs with CpG → TpG/CpA ≥ 0.03825 and CpG → TpG/CpA < 0.03825. A**, **B**, **C**, **D**, **E**, and **F** show the average CpG → TpG/CpA, TpG/CpA → CpG, CpG → GpG/ApG/CpC/CpT, GpG/ApG/CpC/CpT → CpG, A/T → G/C, and G/C → A/T substitution rates in lowly methylated CGIs with a CpG → TpG/CpA substitution rate of ≥0.03825 or a CpG → TpG/CpA substitution rate of <0.03825, respectively. The details of the figure are the same as those described in the legend to Figure 3. CpG → TpG/CpA ≥ 0.03825 shows a CpG → TpG/CpA substitution rate of ≥0.03825.

Moreover, we unexpectedly found that some CGIs showed an average CpG → TpG/CpA substitution rate of ≥0.03825 despite their SPM-LM, indicating that SPM-LM does not necessarily lead to a low CpG → TpG/CpA substitution rate (Figure 4A and Additional file 4: Table S3). Specifically, independent of coding/non-coding and the positions in genes, the average CpG → TpG/CpA substitution rate was more than five times higher in most CGIs with an average CpG → TpG/CpA substitution rate of ≥0.03825 compared with those with an average CpG → TpG/CpA substitution rate of <0.03825, whereas the other substitution rates were approximately two times higher in most CGIs with an average CpG → TpG/CpA substitution rate of ≥0.03825 compared with those with an average CpG → TpG/CpA substitution rate of <0.03825 (Figure 4 and Additional file 4: Table S3). Relatively higher average A/T → G/C and G/C → A/T substitution rates can lead to relatively higher average TpG/CpA → CpG, CpG → GpG/ApG/CpC/CpT, and GpG/ApG/CpC/CpT → CpG substitution rates but not the extremely high average substitution rate of CpG → TpG/CpA (Figure 4 and Additional file 4: Table S3). In other words, similar average substitution rates of A/T → G/C and G/C → A/T should involve similar substitution rates of TpG/CpA → CpG, CpG → GpG/ApG/CpC/CpT, and GpG/ApG/CpC/CpT → CpG but not the extremely high substitution rate of CpG → TpG/CpA. Therefore, these CGIs may be highly methylated in any germline cells. Since CGIs with BGC are generally subject to constitutive methylation and to frequent deamination of cytosine in CpG dinucleotides, it remains to be seen whether these relatively higher A/T → G/C and G/C → A/T substitution rates result from a BGC effect. When we identified CGIs with an average CpG → TpG/CpA substitution rate of ≥0.03984 and SPM-LM using the RRBS data instead of the WGBS data, results similar to those obtained from the WGBS data were obtained, also indicating that SPM-LM does not necessarily lead to a low CpG → TpG/CpA substitution rate (Additional file 5: Figure S2 and Additional file 6: Table S4).

### Contribution of CpG fixation and BGC to maintenance of CpG-rich sequences in CGIs.

**Figure 5:**
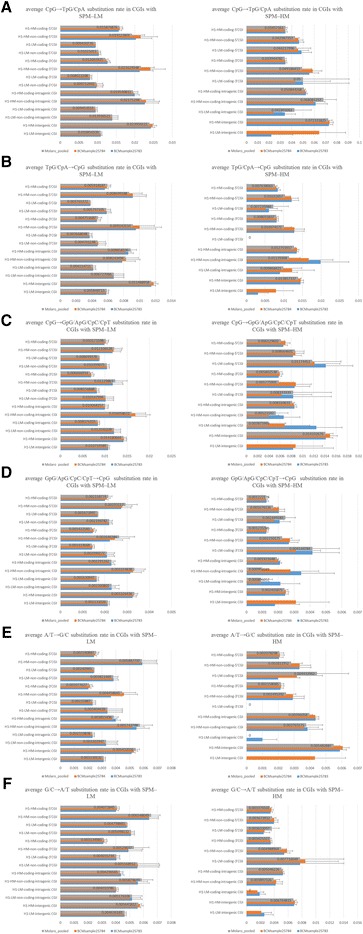
**Comparison of base substitution rates between CGIs with SPM-LM and SPM-HM. A**, **B**, **C**, **D**, **E**, and **F** show the average CpG → TpG/CpA, TpG/CpA → CpG, CpG → GpG/ApG/CpC/CpT, GpG/ApG/CpC/CpT → CpG, A/T → G/C, and G/C → A/T substitution rates in CGIs with SPM-LM or SPM-HM, respectively. The details of the figure are the same as those described in the legend to Figure 4.

We next examined the possibility that frequent CpG fixation and BGC contribute to the maintenance of CpG-rich sequence in sperm methylated CGIs. We used TpG/CpA → CpG and GpG/ApG/CpC/CpT → CpG substitution rates as indicators for CpG fixation, and A/T → G/C and G/C → A/T substitution rates as indicators for BGC. These substitution rates were first compared between highly methylated and hypomethylated CGIs in sperm cells (Additional file 7: Table S5). Figure 5 shows that average CpG → TpG/CpA and TpG/CpA → CpG substitution rates but not the other average substitution rates were higher in almost all CGIs with SPM-HM compared with those with SPM-LM (Figure 5). These results suggest that TpG/CpA → CpG substitution occurs at CGIs according to sperm-methylation followed by frequent CpG → TpG/CpA substitution to compensate CGIs for rapid CpG loss. However, the average TpG/CpA → CpG substitution rate was not much higher in almost all CGIs with SPM-HM compared with those with SPM-LM, and was different from the average CpG → TpG/CpA substitution rate (Figure 5A–5B). Considering these results, CGIs with SPM-HM may preserve their CpG-rich sequences partly by a slightly higher TpG/CpA → CpG substitution rate (Figure 5B). Note that it is unclear whether this TpG/CpA → CpG substitution occurs at newly generated CpG → TpG/CpA substitution sites.

In contrast, the average A/T → G/C substitution rate was relatively higher (~1.5 times) in H1-LM-coding-5′CGIs and H1-LM-intergenic CGIs with SPM-HM compared with those with SPM-LM (Figure 5E). While usual CGIs with BGC (denoted BGC-CGIs) are thought to be highly methylated in both H1 and sperm cells, the sperm methylated H1-LM-coding-5′CGIs and H1-LM-intergenic CGIs are lowly methylated in H1 cells, indicating that BGC does not result from this relatively higher substitution rate (Figure 5E). This suggests little contribution of BGC to the maintenance of CpG-rich sequences in these sperm methylated CGIs classified by genomic positions. It remains unclear why the average A/T → G/C substitution rates for these CGIs are higher in CGIs with SPM-HM than in CGIs with SPM-LM, but this relatively higher A/T → G/C substitution rate may contribute to maintenance of CpG-rich sequences in these CGIs with SPM-HM.

When we similarly examined the contribution of frequent CpG fixation and BGC to the maintenance of CpG-rich sequence in sperm methylated CGIs identified by RRBS instead of WGBS, we also found that most CGIs showed a higher average TpG/CpA → CpG substitution rate and a much higher average CpG → TpG/CpA substitution rate independent of their H1-methylation status in CGIs with SPM-HM compared with those with SPM-LM (Additional file 8: Figure S3 and Additional file 9: Table S6). Moreover, the average A/T → G/C substitution rate was relatively higher (~1.5 times) in some CGIs with SPM-HM compared with those with SPM-LM (Additional file 8: Figure S3E and Additional file 9: Table S6). Since the slightly higher average A/T → G/C substitution rate was not significantly different between highly and lowly methylated CGIs in H1 cells, BGC does not appear to lead to this slightly higher substitution rate (Additional file 8: Figure S3E and Additional file 9: Table S6). These results are similar to those obtained from the WGBS data and support them.

**Figure 6:**
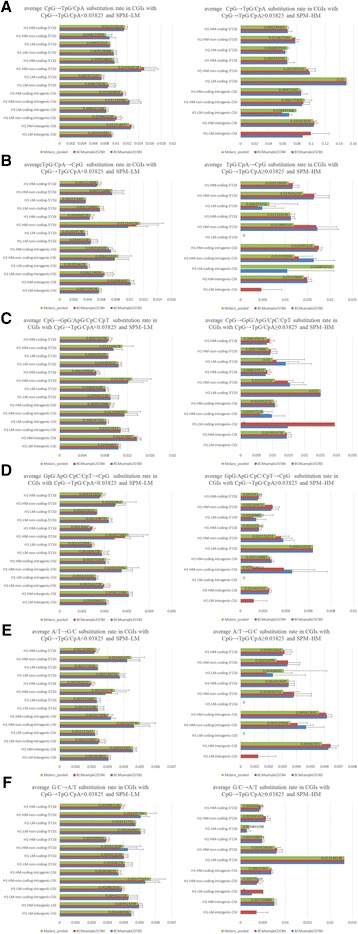
**Comparison of base substitution rates between lowly methylated CGIs with CpG → TpG/CpA < 0.03825 and highly methylated CGIs with CpG → TpG/CpA ≥ 0.03825. A**, **B**, **C**, **D**, **E**, and **F** show the average CpG → TpG/CpA, TpG/CpA → CpG, CpG → GpG/ApG/CpC/CpT, GpG/ApG/CpC/CpT → CpG, A/T → G/C, and G/C → A/T substitution rates in CGIs with a CpG → TpG/CpA substitution rate of <0.03825 and SPM-LM or a CpG → TpG/CpA substitution rate of ≥0.03825 and SPM-HM, respectively. The details of the figure are the same as those described in the legend to Figure 4.

Since BGC-CGIs are constitutively methylated with a rapid cytosine deamination [30], we next used CGIs with SPM-HM and a CpG → TpG/CpA substitution rate of ≥0.03825 (denoted SPM-HM-CpG→TpG/CpA≥0.03825-CGIs) to resemble BGC-CGIs, and used CGIs with SPM-LM and a CpG → TpG/CpA substitution rate of <0.03825 as their hypodeaminated control to examine the contribution of BGC to the maintenance of CpG-rich sequences in sperm methylated CGIs (Figure 6). Most CGIs showed a higher average TpG/CpA → CpG substitution rate and a much higher average CpG → TpG/CpA substitution rate independent of their H1-methylation status in SPM-HM-CpG→TpG/CpA≥0.03825-CGIs compared with the control (Figure 6 and Additional file 1: Table S1 and Additional file 2: Table S2). These results also suggest that TpG/CpA → CpG substitution occurs at CGIs according to sperm-methylation followed by frequent CpG → TpG/CpA substitution to compensate CGIs for rapid CpG loss.

In addition, some CGIs showed a slightly higher average A/T → G/C substitution rate independent of their H1-methylation status in SPM-HM-CpG→TpG/CpA≥0.03825-CGIs compared with the control (Figure 6E). Since the slightly higher average A/T → G/C substitution rate was not significantly different between CGIs with H1-LM and H1-HM, BGC does not appear to lead to this slightly higher substitution rate (Figure 6E–6F and Additional file 1: Table S1 and Additional file 2: Table S2). It also remains unclear why the average A/T → G/C substitution rates for these CGIs are higher in SPM-HM-CpG→TpG/CpA≥0.03825-CGIs than in the control, but this slightly higher A/T → G/C substitution rate may contribute to maintenance of CpG-rich sequences in these CGIs (Figure 6E–6F, Additional file 1: Table S1 Additional file 2: Table S2). These results also suggest little contribution of BGC to the maintenance of CpG-rich sequences in SPM-HM-CpG→TpG/CpA≥0.03825-CGIs.

We next replaced the WGBS data with the RRBS data, and identified CGIs with SPM-HM and a CpG → TpG/CpA substitution rate of ≥0.03984 (denoted SPM-HM-CpG→TpG/CpA≥0.03984-CGIs) and their hypodeaminated control (i.e., CGIs with SPM-LM and a CpG → TpG/CpA substitution rate of <0.03984) in the same way as described above. Then, the average substitution rates were compared between SPM-HM-CpG→TpG/CpA≥0.03984-CGIs and the hypodeaminated control. Consequently, we also found that most CGIs showed a higher average TpG/CpA → CpG substitution rate and a much higher average CpG → TpG/CpA substitution rate independent of their H1-methylation status in SPM-HM-CpG→TpG/CpA≥0.03984-CGIs compared with the control (Additional file 10: Figure S4, Additional file 2: Table S2 and Additional file 6: Table S4). Moreover, although for some CGIs, the average A/T → G/C substitution rate was relatively higher (~1.5 times) in CGIs with SPM-HM compared with those with SPM-LM, the slightly higher average A/T → G/C substitution rate was not significantly different between highly and lowly methylated CGIs in H1 cells (Additional file 10: Figure S4E). Consequently, BGC does not appear to lead to this slightly higher substitution rate (Additional file 8: Figure S3E and Additional file 9: Table S6). These results are consistent with those obtained from the WGBS data.

Considering these results, CGIs with SPM-HM may maintain their CpG-rich sequences, at least in part, by CpG selection and a slightly high TpG/CpA → CpG, fixation or they might disappear from the human genome in the future, because they have extremely frequent CpG → TpG/CpA fixation.

The CGIs shown in Additional file 1: Table S1, Additional file 2: Table S2, Additional file 4: Table S3, Additional file 6: Table S4, Additional file 7: Table S5 and Additional file 9: Table S6 are listed in Additional file 11: Table S7 and Additional file 12: Table S8. Chromosomal positions of CGI IDs in Additional file 11: Table S7 and Additional file 12: Table S8 are shown in Additional file 13: Table S9.

### Removing hydroxymethylated CGIs from all the CGIs

Hydroxymethylated CpG dinucleotides convert to TpG dinucleotides less frequently than methylated CpG dinucleotides. In addition, bisulfite sequencing technique cannot distinguish between hydroxymethylated and methylated CpG dinucleotides. Consequently, hydroxymethylated CGIs should be removed from methylated CGIs identified by WGBS in germline and non-germline cells, because hydroxymethylated CpG dinucleotides have a lower CpG → TpG/CpA substitution rate compared with methylated CpG dinucleotides. We thus worked to remove CGIs with at least one 5hmC from all the CGIs used above and conduct the same analysis as described above. However, we were able to obtain whole-genome 5hmC data at single-base resolution from H1 cell line but not those from mature human sperm cells. We therefore removed CGIs with at least one 5hmC in H1 cell line (denoted as H1_5hmC_CGIs) from all the CGIs and conducted the same analysis as described above.

As a result, similar to results obtained from all the CGIs including H1_5hmC_CGIs (denoted All_CGIs_including_5hmC), more than half the numbers (approximately 55–75%) of CGIs with H1-HM in all the genomic positions showed a CpG → TpG/CpA substitution rate of <0.03984 (Additional file 14: Table S10). Note that a CpG → TpG/CpA substitution rate of 0.03984, which is determined by a decision tree, is used as a threshold to infer whether or not CGIs have a low CpG → TpG/CpA substitution rate. In contrast, fewer than half the numbers (10–47%) of CGIs with H1-HM in all the genomic positions showed a CpG → TpG/CpA substitution rate of <0.03984 and SPM-LM (Additional file 14: Table S10). These results also suggest that more than half the numbers of CGIs with H1-HM in all the genomic positions maintain their CpG-rich sequences by a low CpG → TpG/CpA substitution rate caused not only by their SPM-LM but also by another mechanism, such as CpG selection.

In addition, similar to results obtained from All_CGIs_including_5hmC, only H1-HM-non-coding-3′CGIs and H1-HM-non-coding-intragenic CGIs had CpG selection, because the average CpG → GpG/ApG/CpC/CpT substitution rate, but not the other average substitution rates, is extremely low in the above CGIs (Additional file 15: Figure S5 and Additional file 14: Table S10).

Moreover, some CGIs showed an average CpG → TpG/CpA substitution rate of ≥0.03984 despite their SPM-LM (Additional file 16: Table S11). Specifically, independent of the positions in genes, the average CpG → TpG/CpA substitution rate was more than five times higher in CGIs with an average CpG → TpG/CpA substitution rate of ≥0.03984 and SPM-LM compared with those with an average CpG → TpG/CpA substitution rate of <0.03984 and SPM-LM, whereas the other substitution rates were approximately two times higher in most CGIs with an average CpG → TpG/CpA substitution rate of ≥0.03984 compared with those with an average CpG → TpG/CpA substitution rate of <0.03984 (Additional file 17: Figure S6 and Additional file 14: Table S10 and Additional file 16: Table S11). This also indicates that SPM-LM does not necessarily lead to a low CpG → TpG/CpA substitution rate.

As for CpG fixation and BGC, for CGIs in almost all the genomic positions, the average CpG → TpG/CpA and TpG/CpA → CpG substitution rates but not the other average substitution rates were much higher and relatively higher in CGIs with SPM-HM compared with those with SPM-LM, respectively (Additional file 18: Figure S7A–S7B and Additional file 19: Table S12). Consequently, it is suggested that CpG fixation from TpG/CpA, at least in part, contributes to maintenance of CpG-rich sequences on CGIs with SPM-HM, similar to results obtained from All_CGIs_including_5hmC.

The average A/T → G/C substitution rate was relatively higher (~1.5 times) in H1-LM-coding-5′CGIs and H1-LM-intergenic CGIs with SPM-HM compared with those with SPM-LM (Additional file 18: Figure S7E and Additional file 19: Table S12). However, since both the sperm methylated H1-LM-coding-5′CGIs and H1-LM-intergenic CGIs are lowly methylated in H1 cells, BGC does not appear to result from this relatively higher substitution rate, similar to results obtained from All_CGIs_including_5hmC (Additional file 18: Figure S7E and Additional file 19: Table S12). Similarly, CGIs in most genomic positions showed a higher average TpG/CpA → CpG substitution rate and a much higher average CpG → TpG/CpA substitution rate independent of their H1-methylation status in CGIs with SPM-HM-CpG→TpG/CpA≥0.03984-CGIs compared with their hypodeaminated control (i.e., CGIs with SPM-LM and a CpG → TpG/CpA substitution rate of <0.03984) (Additional file 20: Figure S8A–S8B and Additional file 14: Table S10 and Additional file 16: Table S11).

In addition, CGIs in some genomic positions showed a slightly higher average A/T → G/C substitution rate independent of their H1-methylation status in SPM-HM-CpG→TpG/CpA≥0.03984-CGIs compared with the control (Additional file 20: Figure S8E). Since the slightly higher average A/T → G/C substitution rate was not significantly different between highly and lowly methylated CGIs in H1 cells, BGC does not appear to lead to this slightly higher substitution rate (Additional file 20: Figure S8E, Additional file 14: Table S10 and Additional file 16: Table S11).

These results indicate that removing H1_5hmC_CGIs from All_CGIs_including_5hmC has negligible impact on the conclusions obtained from All_CGIs_including_5hmC.

The CGIs shown in Additional file 14: Table S10, Additional file 16: Table S11, Additional file 19: Table S12 are listed in Additional file 21: Table S13. Chromosomal positions of CGI IDs in Additional file 21: Table S13 are shown in Additional file 13: Table S9.

### Biological roles of sperm LM for H1-HM-coding-5′CGIs with a CpG → TpG/CpA substitution rate of <0.03825

**Figure 7:**
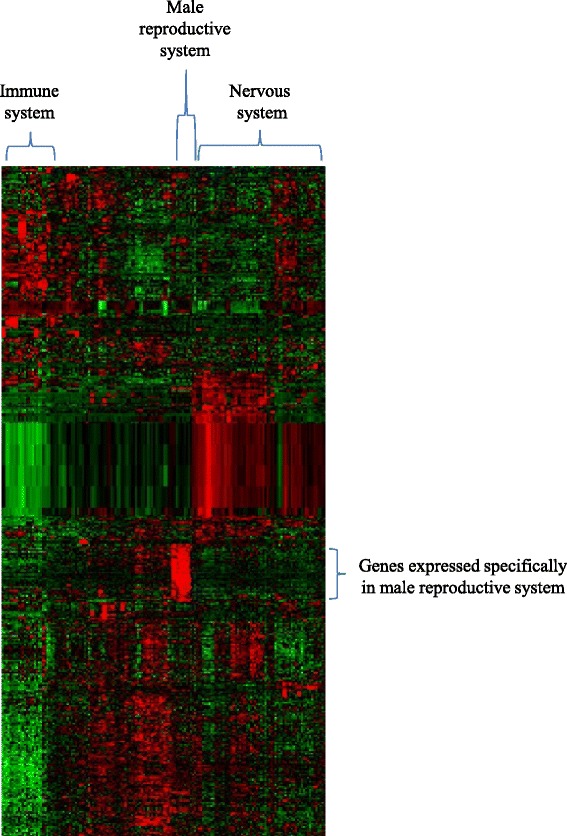
**A gene cluster with high expression only in male reproductive system-related tissues.** The horizontal axis shows adult tissue samples, and the vertical axis denotes genes associated with H1-HM-coding-5′CGIs with a CpG → TpG/CpA substitution rate of <0.03825 and SPM-LM. Red and green colors describe high and low expression of a gene, respectively.

As explained above, when we examined the contribution of SPM-LM to a low CpG → TpG/CpA substitution rate in CGIs with H1-HM using All_CGIs_including_5hmC and the WGBS data, about half the numbers of H1-HM-coding-5′CGIs showed an average CpG → TpG/CpA substitution rate of <0.03825 and SPM-LM. Therefore, SPM-LM for these CGIs seems to be for maintenance of CpGs through their slow CpG → TpG/CpA substitution rates. However, it is uncertain whether SPM-LM is also necessary for sperm-specific expression of these CGIs-associated genes. We thus clustered tissue expression of genes associated with H1-HM-coding-5′CGIs with an average CpG → TpG/CpA substitution rate of <0.03825 and SPM-LM (*N* = 464; these CGIs are hypomethylated in all the sperm samples). As shown in Figure 7, only a fraction of the genes showed a high expression in male reproductive system-related tissues, suggesting that most SPM-LM on these H1-HM-coding-5′CGIs with an average CpG → TpG/CpA substitution rate of <0.03825 and SPM-LM are for maintenance of their CpG rich sequences but not for sperm-specific expression of their associated genes.

## Conclusion

More than half the numbers of CGIs with H1-HM in all the genomic positions maintain their CpG-rich sequences by a low CpG → TpG/CpA substitution rate caused mainly by their SPM-LM, but when these have SPM-HM, CpG selection and relatively higher TpG/CpA → CpG fixation partly compensate them for frequent CpG loss. BGC does not contribute to the maintenance of CpG-rich sequences of CGIs with SPM-HM which were classified by genomic positions.

## Additional files

Additional file 1: Table S1. The table shows the number of CGIs with a CpG → TpG/CpA substitution rate of <0.03825 for each methylation status in mature sperm cells identified by the WGBS and their average base substitution rates. Additional file 2: Table S2. The table shows the number of CGIs with a CpG → TpG/CpA substitution rate of <0.03984 for each methylation status in mature sperm cells identified by the RRBS and their average base substitution rates. Additional file 3: Figure S1. Base substitution rate in sperm methylated CGIs with CpG → TpG/CpA < 0.03984. Methylation levels of CGIs in H1 and sperm cells are calculated using the RRBS data. The prefixes of H1-LM- and H1-HM- show low and high methylation of CGIs in H1 cells, respectively. Coding- and non-coding- indicate CGIs that associate with protein-coding and non-coding genes, respectively. RRBS_Sperm3 and RRBS_Sperm4 describe separate human sperm samples. SPM-HM shows CGIs with a high methylation of ≥80% in sperm cells. Error bars represent standard errors. CpG → TpG/CpA < 0.03984 shows a CpG → TpG/CpA substitution rate of <0.03984. Additional file 4: Table S3. The table shows the number of CGIs with a CpG → TpG/CpA substitution rate of ≥0.03825 for each methylation status in mature sperm cells identified by the WGBS and their average base substitution rates. Additional file 5: Figure S2. Comparison of base substitution rates between lowly methylated CGIs with CpG → TpG/CpA ≥ 0.03984 and CpG → TpG/CpA < 0.03984. The details of the figure are the same as those described in the legend to Additional file 3: Figure S1. CpG → TpG/CpA ≥ 0.03984 shows a CpG → TpG/CpA substitution rate of ≥0.03984. Additional file 6: Table S4. The table shows the number of CGIs with a CpG → TpG/CpA substitution rate of ≥0.03984 for each methylation status in mature sperm cells identified by RRBS and their average base substitution rates. Additional file 7: Table S5. The table shows the number of CGIs for each methylation status in mature sperm cells identified by the GBS and their average base substitution rates. Additional file 8: Figure S3. Comparison of base substitution rates between CGIs with SPM-LM and SPM-HM. The details of the figure are the same as those described in the legend to Additional file 5: Figure S2. Additional file 9: Table S6. The table shows the number of CGIs for each methylation status in mature sperm cells identified by the RRBS and their average base substitution rates. Additional file 10: Figure S4. Comparison of base substitution rates between lowly methylated CGIs with CpG → TpG/CpA < 0.03984 and highly methylated CGIs with CpG → TpG/CpA ≥ 0.03984. The details of the figure are the same as those described in the legend to Additional file 5: Figure S2. Additional file 11: Table S7. The CGIs shown in Additional file 1: Table S1, Additional file 4: Table S3 and Additional file 7: Table S5 are listed in the table. Additional file 12: Table S8. The CGIs shown in Additional file 2: Table S2, Additional file 6: Table S4 and Additional file 9: Table S6 are listed in the table. Additional file 13: Table S9. Chromosomal positions of CGI IDs in Additional file 11: Table S7 and Additional file 12: Table S8 are shown in the table. Additional file 14: Table S10. The table shows the number of CGIs with a CpG → TpG/CpA substitution rate of <0.03984 for each methylation status in mature sperm cells identified by WGBS and their average base substitution rates. Note that hydroxymethylated CGIs in H1 cells are removed in these CGIs. Additional file 15: Figure S5. Base substitution rate in sperm methylated CGIs with CpG → TpG/CpA < 0.03984. Hydroxymethylated CGIs in H1 cells are not included in these CGIs. The details of the figure are the same as those described in the legend to Figure 3. Additional file 16: Table S11. The table shows the number of CGIs with a CpG → TpG/CpA substitution rate of ≥0.03984 for each methylation status in mature sperm cells identified by WGBS and their average base substitution rates. Note that the hydroxymethylated CGIs in H1 cells are removed in these CGIs. Additional file 17: Figure S6. Comparison of base substitution rates between lowly methylated CGIs with CpG → TpG/CpA ≥ 0.03984 and CpG → TpG/CpA < 0.03984. Hydroxymethylated CGIs in H1 cells are not included in these CGIs. The details of the figure are the same as those described in the legend to Figure 4. Additional file 18: Figure S7. Comparison of base substitution rates between CGIs with SPM-LM and SPM-HM. Hydroxymethylated CGIs in H1 cells are not included in these CGIs. The details of the figure are the same as those described in the legend to Figure 5. Additional file 19: Table S12. The table shows the number of CGIs for each methylation status in mature sperm cells identified by WGBS and their average base substitution rates. Note that the hydroxymethylated CGIs in H1 cells are removed in these CGIs. Additional file 20: Figure S8. Comparison of base substitution rates between lowly methylated CGIs with CpG → TpG/CpA < 0.03984 and highly methylated CGIs with CpG → TpG/CpA ≥ 0.03984. Hydroxymethylated CGIs in H1 cells are not included in these CGIs. The details of the figure are the same as those described in the legend to Figure 6. Additional file 21: Table S13. The CGIs shown in Additional file 4: Table S10, Additional file 16: Table S11 and Additional file 19: Table S12 are listed in the table.

## Abbreviations

CGIs: CpG islands
BGC: Biased gene conversion
LM: A low methylation level of ≤20%
HM: A high methylation level of ≥80%
SPM-LM: Low levels of methylation in sperm cells
SPM-HM: High levels of methylation in sperm cells
H1-LM: Low levels of methylation in H1 cells
WGBS: Whole genome bisulfite sequencing
ICRs: Imprinting control regions
RRBS: Reduced representation bisulfite sequencing
H1-HM-coding-5′CGIs: CGIs with high methylation in H1 cells, associated with the promoter of a protein coding gene
SPM-HM-CpG→TpG/CpA≥0.03825-CGIs: CGIs with SPM-HM and a CpG → TpG/CpA substitution rate of ≥0.03825
SPM-HM-CpG→TpG/CpA≥0.03984-CGIs: CGIs with SPM-HM and a CpG→TpG/CpA substitution rate of ≥0.03984
H1_5hmC_CGIs: CGIs with at least one 5hmC
All_CGIs_including_5hmC: all CGIs including H1_5hmC_CGIs used in this study
