# Supplementary material for: Factors to preserve CpG-rich sequences in methylated CpG islands
Source: BMC Genomics. 2015 Feb 28;16(1):144. doi: 10.1186/s12864-015-1286-x (PMC4417305; doi:10.1186/s12864-015-1286-x)
Supplement: Additional file 3: Figure S1. — Base substitution rate in sperm methylated CGIs with CpG → TpG/CpA < 0.03984. Methylation levels of CGIs in H1 and sperm cells are calculated using the RRBS data. The prefixes of H1-LM- and H1-HM- show low and high methylation of CGIs in H1 cells, respectively. Coding- and non-coding- indicate CGIs that associate with protein-coding and non-coding genes, respectively. RRBS_Sperm3 and RRBS_Sperm4 describe separate human sperm samples. SPM-HM shows CGIs with a high methylation of ≥80% in sperm cells. Error bars represent standard errors. CpG → TpG/CpA < 0.03984 shows a CpG → TpG/CpA substitution rate of <0.03984. [file 12864_2015_1286_MOESM3_ESM.pdf]

A

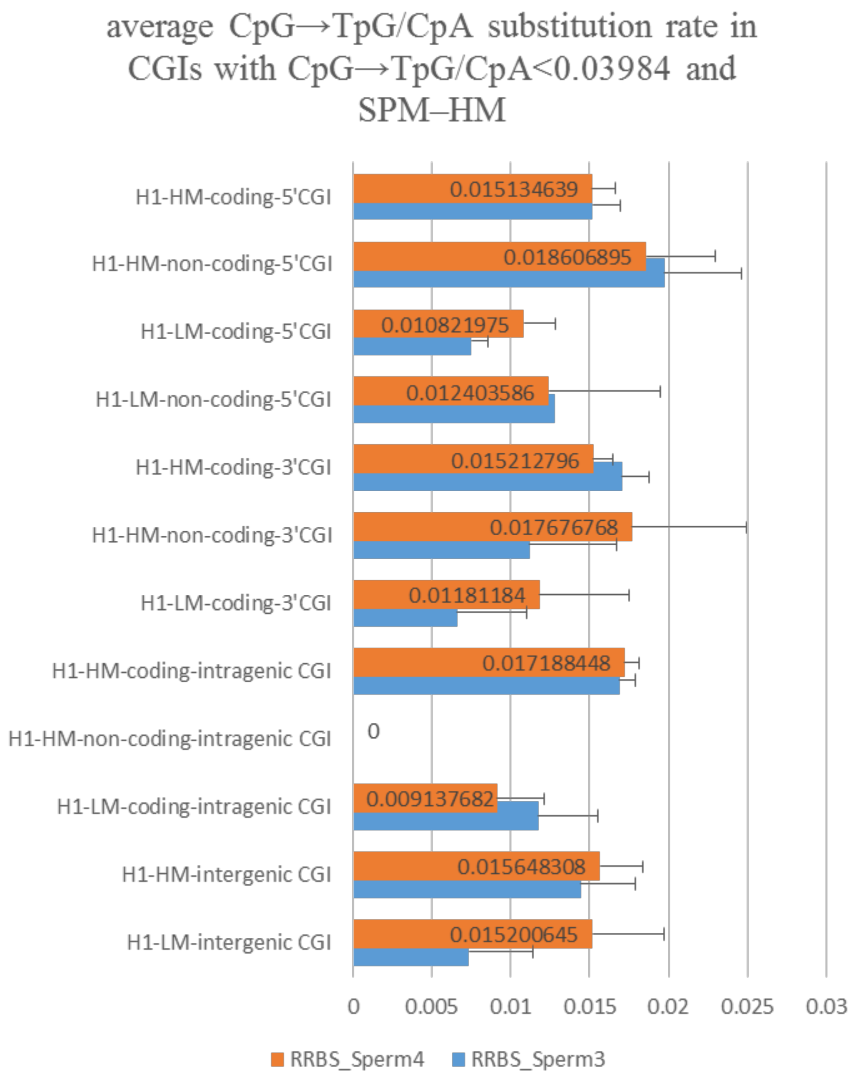

average CpG→TpG/CpA substitution rate in CGIs with CpG→TpG/CpA<0.03984 and SPM–HM
H1-HM-coding-5'CGI
H1-HM-non-coding-5'CGI
H1-LM-coding-5'CGI
H1-LM-non-coding-5'CGI
H1-HM-coding-3'CGI
H1-HM-non-coding-3'CGI
H1-LM-coding-3'CGI
H1-HM-coding-intragenic CGI
H1-HM-non-coding-intragenic CGI
H1-LM-coding-intragenic CGI
H1-HM-intergenic CGI
H1-LM-intergenic CGI
0.015134639
0.018606895
0.010821975
0.012403586
0.015212796
0.017676768
0.01181184
0.017188448
0
0.009137682
0.015648308
0.015200645
0
0.005
0.01
0.015
0.02
0.025
0.03
RRBS_Sperm4
RRBS_Sperm3

B

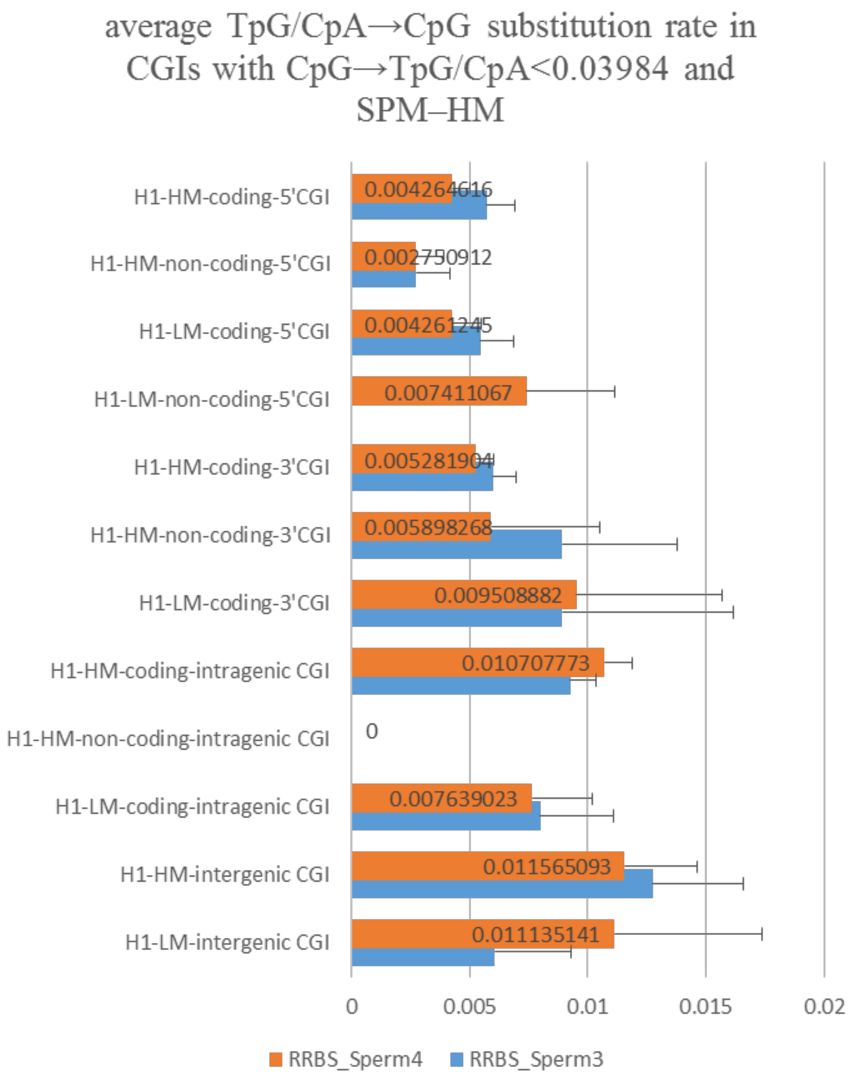

average TpG/CpA→CpG substitution rate in CGIs with CpG→TpG/CpA<0.03984 and SPM–HM
H1-HM-coding-5'CGI
H1-HM-non-coding-5'CGI
H1-LM-coding-5'CGI
H1-LM-non-coding-5'CGI
H1-HM-coding-3'CGI
H1-HM-non-coding-3'CGI
H1-LM-coding-3'CGI
H1-HM-coding-intragenic CGI
H1-HM-non-coding-intragenic CGI
H1-LM-coding-intragenic CGI
H1-HM-intergenic CGI
H1-LM-intergenic CGI
0.004264616
0.002750912
0.004261245
0.007411067
0.005281904
0.005898268
0.009508882
0.010707773
0
0.007639023
0.011565093
0.011135141
0
0.005
0.01
0.015
0.02
RRBS_Sperm4
RRBS_Sperm3

C

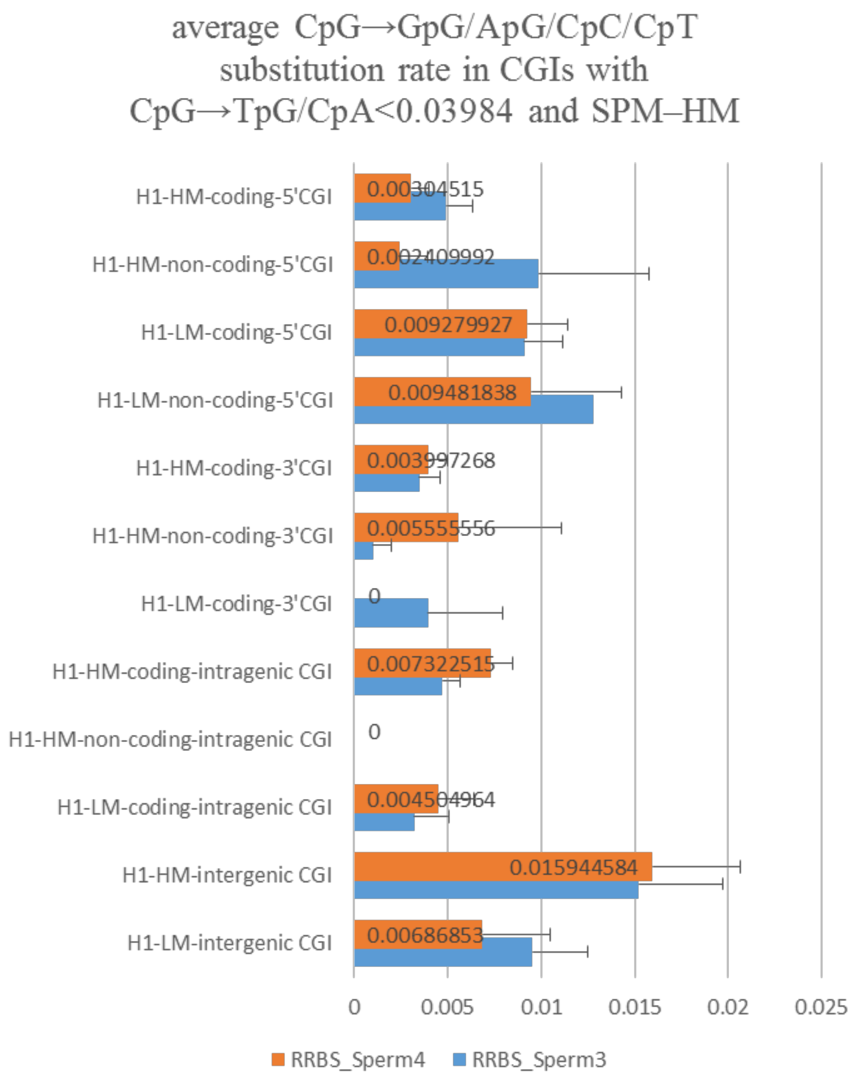
average CpG→GpG/ApG/CpC/CpT
substitution rate in CGIs with
CpG→TpG/CpA<0.03984 and SPM–HM
H1-HM-coding-5'CGI
H1-HM-non-coding-5'CGI
H1-LM-coding-5'CGI
H1-LM-non-coding-5'CGI
H1-HM-coding-3'CGI
H1-HM-non-coding-3'CGI
H1-LM-coding-3'CGI
H1-HM-coding-intragenic CGI
H1-HM-non-coding-intragenic CGI
H1-LM-coding-intragenic CGI
H1-HM-intergenic CGI
H1-LM-intergenic CGI
0.00304515
0.002409992
0.009279927
0.009481838
0.003997268
0.005555556
0
0.007322515
0
0.004504964
0.015944584
0.00686853
0
0.005
0.01
0.015
0.02
0.025
RRBS_Sperm4
RRBS_Sperm3

D

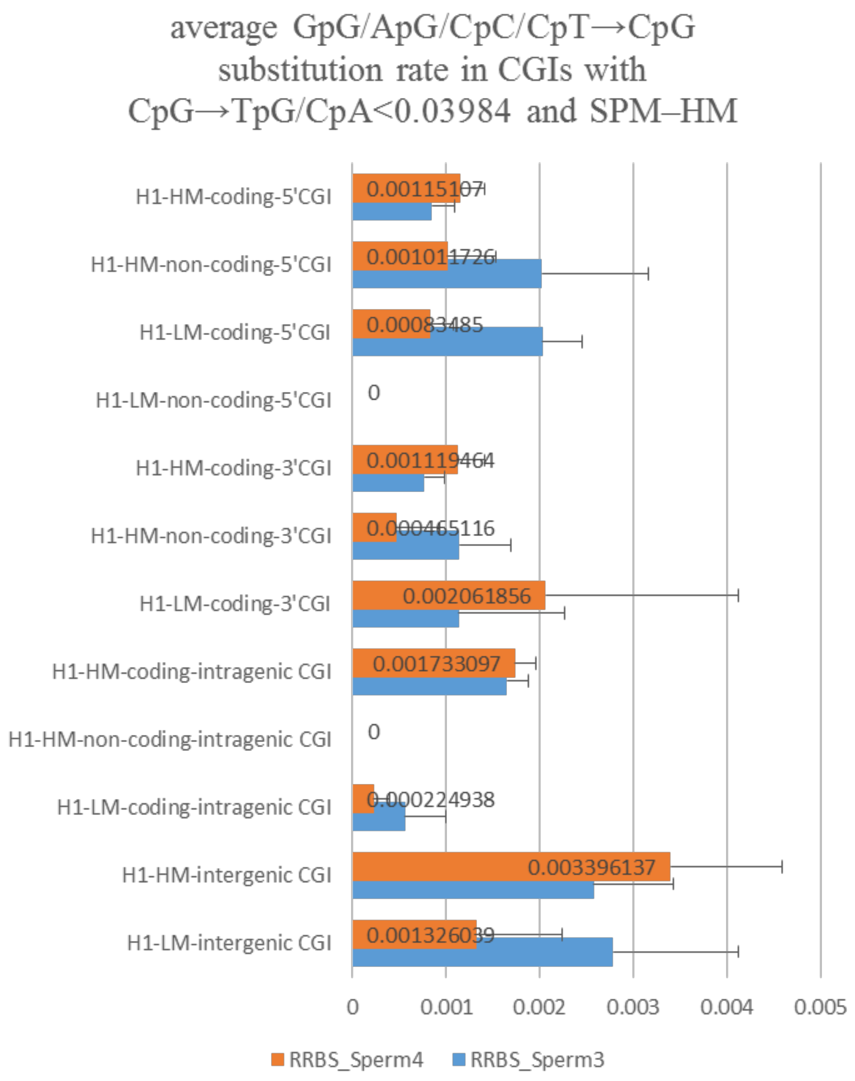
average GpG/ApG/CpC/CpT→CpG
substitution rate in CGIs with
CpG→TpG/CpA<0.03984 and SPM–HM
H1-HM-coding-5'CGI
H1-HM-non-coding-5'CGI
H1-LM-coding-5'CGI
H1-LM-non-coding-5'CGI
H1-HM-coding-3'CGI
H1-HM-non-coding-3'CGI
H1-LM-coding-3'CGI
H1-HM-coding-intragenic CGI
H1-HM-non-coding-intragenic CGI
H1-LM-coding-intragenic CGI
H1-HM-intergenic CGI
H1-LM-intergenic CGI
0.00115107
0.001011726
0.00083485
0
0.001119464
0.000465116
0.002061856
0.001733097
0
0.000224938
0.003396137
0.001326039
0
0.001
0.002
0.003
0.004
0.005
RRBS_Sperm4
RRBS_Sperm3

E

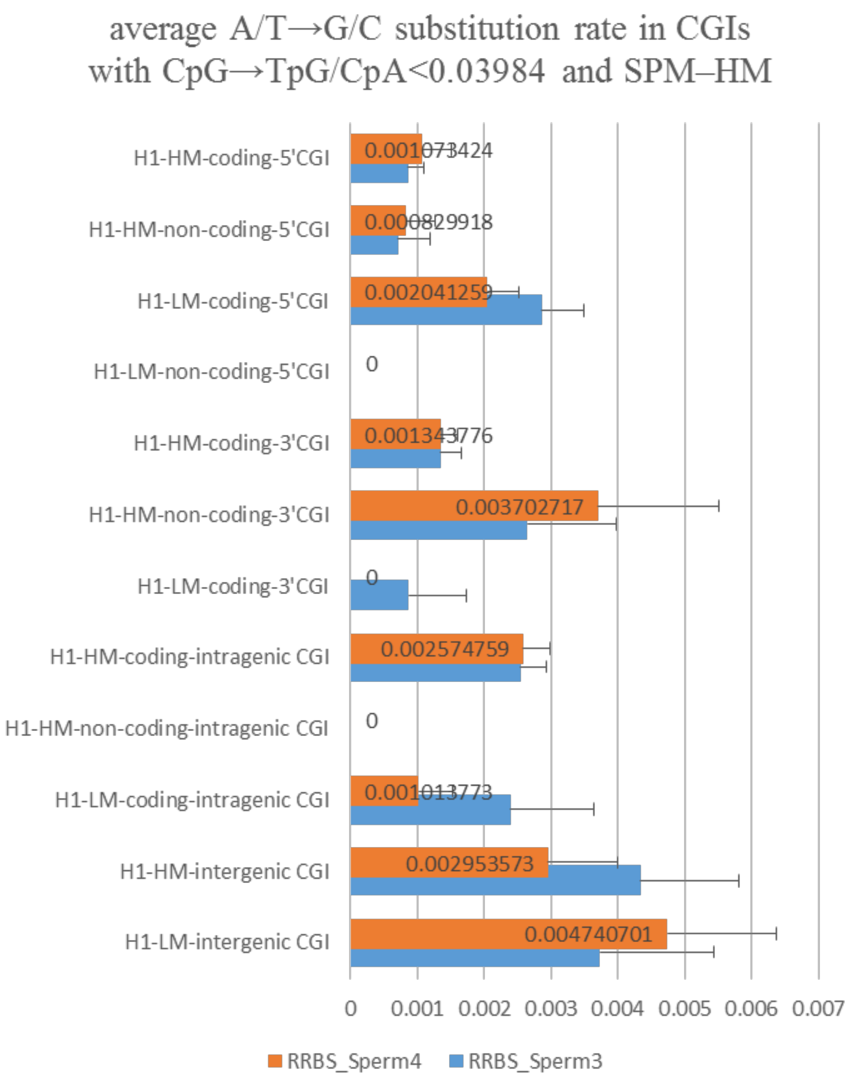

average A/T→G/C substitution rate in CGIs
with CpG→TpG/CpA<0.03984 and SPM–HM
H1-HM-coding-5'CGI
H1-HM-non-coding-5'CGI
H1-LM-coding-5'CGI
H1-LM-non-coding-5'CGI
H1-HM-coding-3'CGI
H1-HM-non-coding-3'CGI
H1-LM-coding-3'CGI
H1-HM-coding-intragenic CGI
H1-HM-non-coding-intragenic CGI
H1-LM-coding-intragenic CGI
H1-HM-intergenic CGI
H1-LM-intergenic CGI
0.001073424
0.000829918
0.002041259
0
0.001343776
0.003702717
0
0.002574759
0
0.001013773
0.002953573
0.004740701
0
0.001
0.002
0.003
0.004
0.005
0.006
0.007
RRBS_Sperm4
RRBS_Sperm3

F

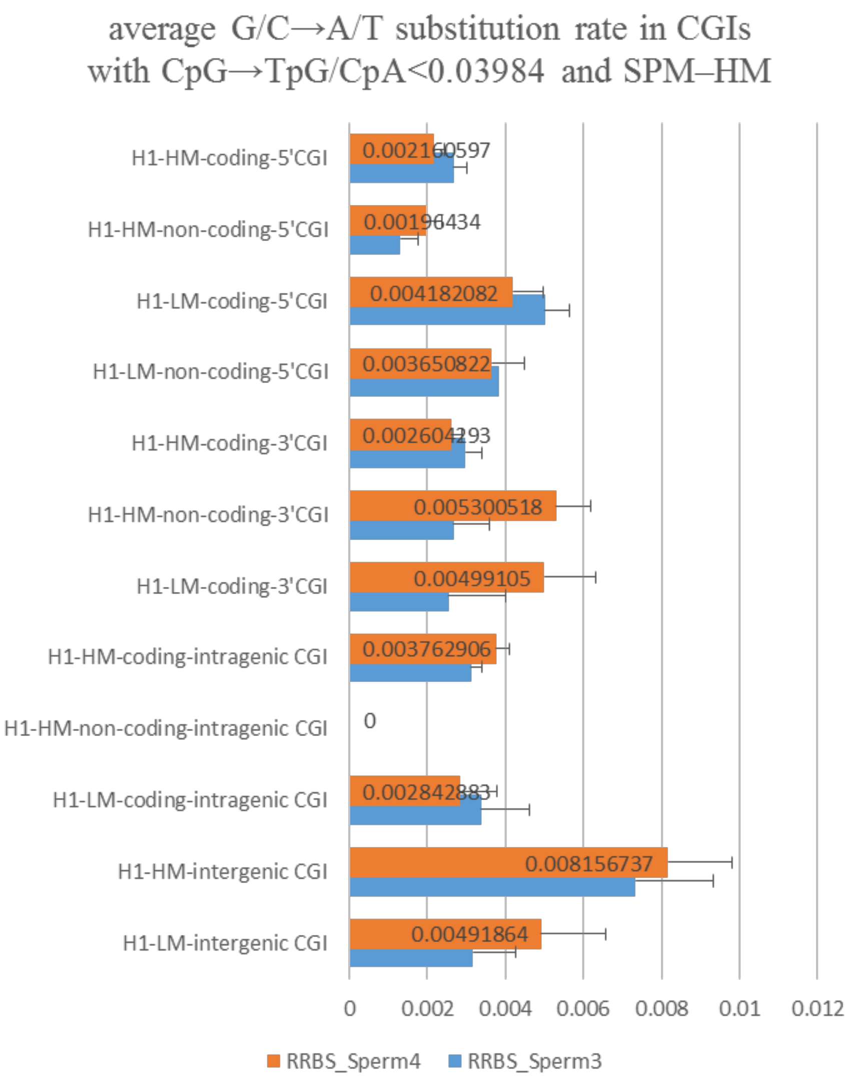

average G/C→A/T substitution rate in CGIs
with CpG→TpG/CpA<0.03984 and SPM–HM
H1-HM-coding-5'CGI
H1-HM-non-coding-5'CGI
H1-LM-coding-5'CGI
H1-LM-non-coding-5'CGI
H1-HM-coding-3'CGI
H1-HM-non-coding-3'CGI
H1-LM-coding-3'CGI
H1-HM-coding-intragenic CGI
H1-HM-non-coding-intragenic CGI
H1-LM-coding-intragenic CGI
H1-HM-intergenic CGI
H1-LM-intergenic CGI
0.002160597
0.00196434
0.004182082
0.003650822
0.002604293
0.005300518
0.00499105
0.003762906
0
0.002842883
0.008156737
0.00491864
0
0.002
0.004
0.006
0.008
0.01
0.012
RRBS_Sperm4
RRBS_Sperm3
