# Supplementary material for: Factors to preserve CpG-rich sequences in methylated CpG islands
Source: BMC Genomics. 2015 Feb 28;16(1):144. doi: 10.1186/s12864-015-1286-x (PMC4417305; doi:10.1186/s12864-015-1286-x)
Supplement: Additional file 5: Figure S2. — Comparison of base substitution rates between lowly methylated CGIs with CpG → TpG/CpA ≥ 0.03984 and CpG → TpG/CpA < 0.03984. The details of the figure are the same as those described in the legend to Additional file 3: Figure S1. CpG → TpG/CpA ≥ 0.03984 shows a CpG → TpG/CpA substitution rate of ≥0.03984. [file 12864_2015_1286_MOESM5_ESM.pdf]

A

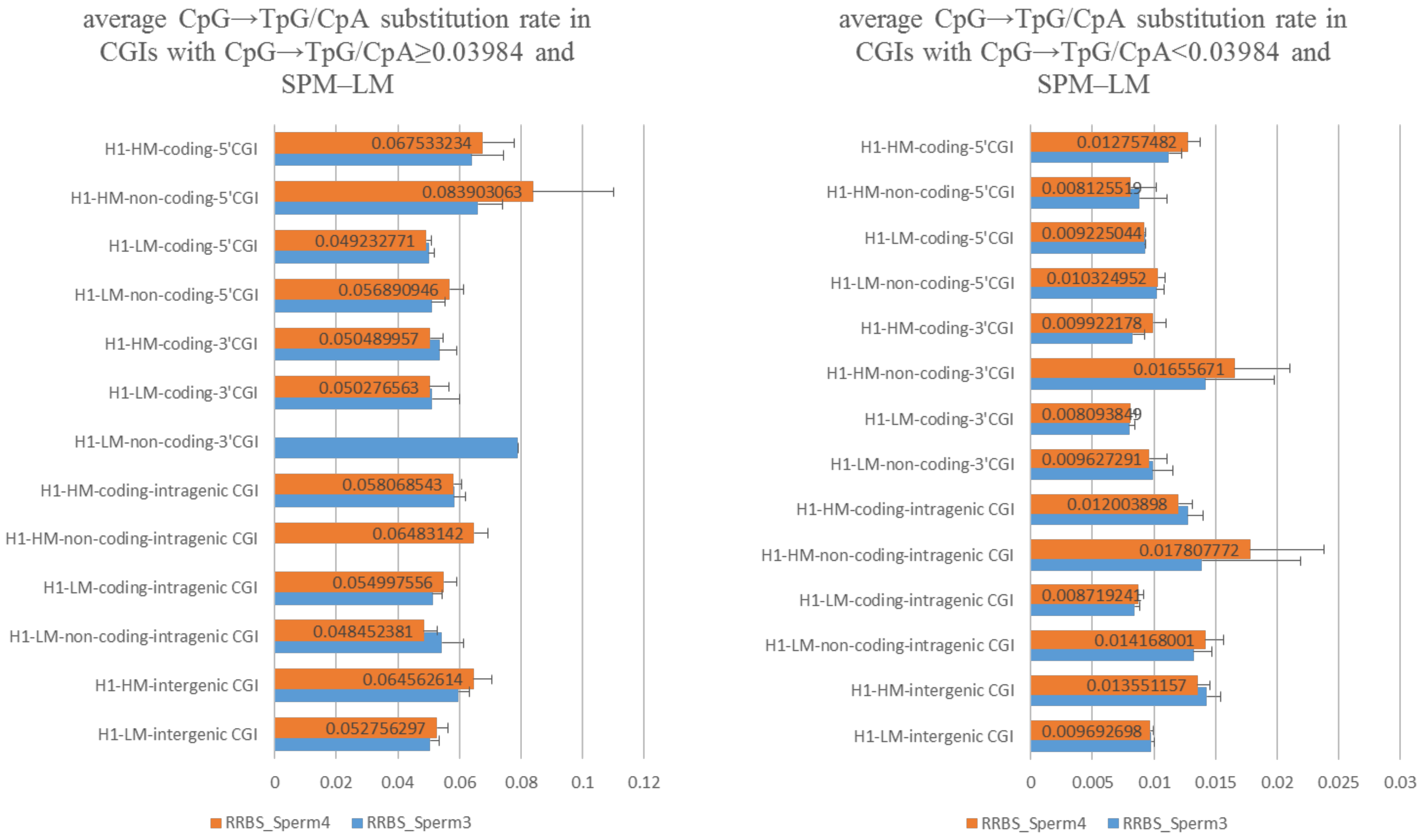

average CpG→TpG/CpA substitution rate in CGIs with CpG→TpG/CpA≥0.03984 and SPM–LM
H1-HM-coding-5'CGI
H1-HM-non-coding-5'CGI
H1-LM-coding-5'CGI
H1-LM-non-coding-5'CGI
H1-HM-coding-3'CGI
H1-LM-coding-3'CGI
H1-LM-non-coding-3'CGI
H1-HM-coding-intragenic CGI
H1-HM-non-coding-intragenic CGI
H1-LM-coding-intragenic CGI
H1-LM-non-coding-intragenic CGI
H1-HM-intergenic CGI
H1-LM-intergenic CGI
0.067533234
0.083903063
0.049232771
0.056890946
0.050489957
0.050276563
0.058068543
0.06483142
0.054997556
0.048452381
0.064562614
0.052756297
0
0.02
0.04
0.06
0.08
0.1
0.12
RRBS_Sperm4
RRBS_Sperm3
average CpG→TpG/CpA substitution rate in CGIs with CpG→TpG/CpA<0.03984 and SPM–LM
H1-HM-coding-5'CGI
H1-HM-non-coding-5'CGI
H1-LM-coding-5'CGI
H1-LM-non-coding-5'CGI
H1-HM-coding-3'CGI
H1-HM-non-coding-3'CGI
H1-LM-coding-3'CGI
H1-LM-non-coding-3'CGI
H1-HM-coding-intragenic CGI
H1-HM-non-coding-intragenic CGI
H1-LM-coding-intragenic CGI
H1-LM-non-coding-intragenic CGI
H1-HM-intergenic CGI
H1-LM-intergenic CGI
0.012757482
0.008125519
0.009225044
0.010324952
0.009922178
0.01655671
0.008093849
0.009627291
0.012003898
0.017807772
0.008719241
0.014168001
0.013551157
0.009692698
0
0.005
0.01
0.015
0.02
0.025
0.03
RRBS_Sperm4
RRBS_Sperm3

B

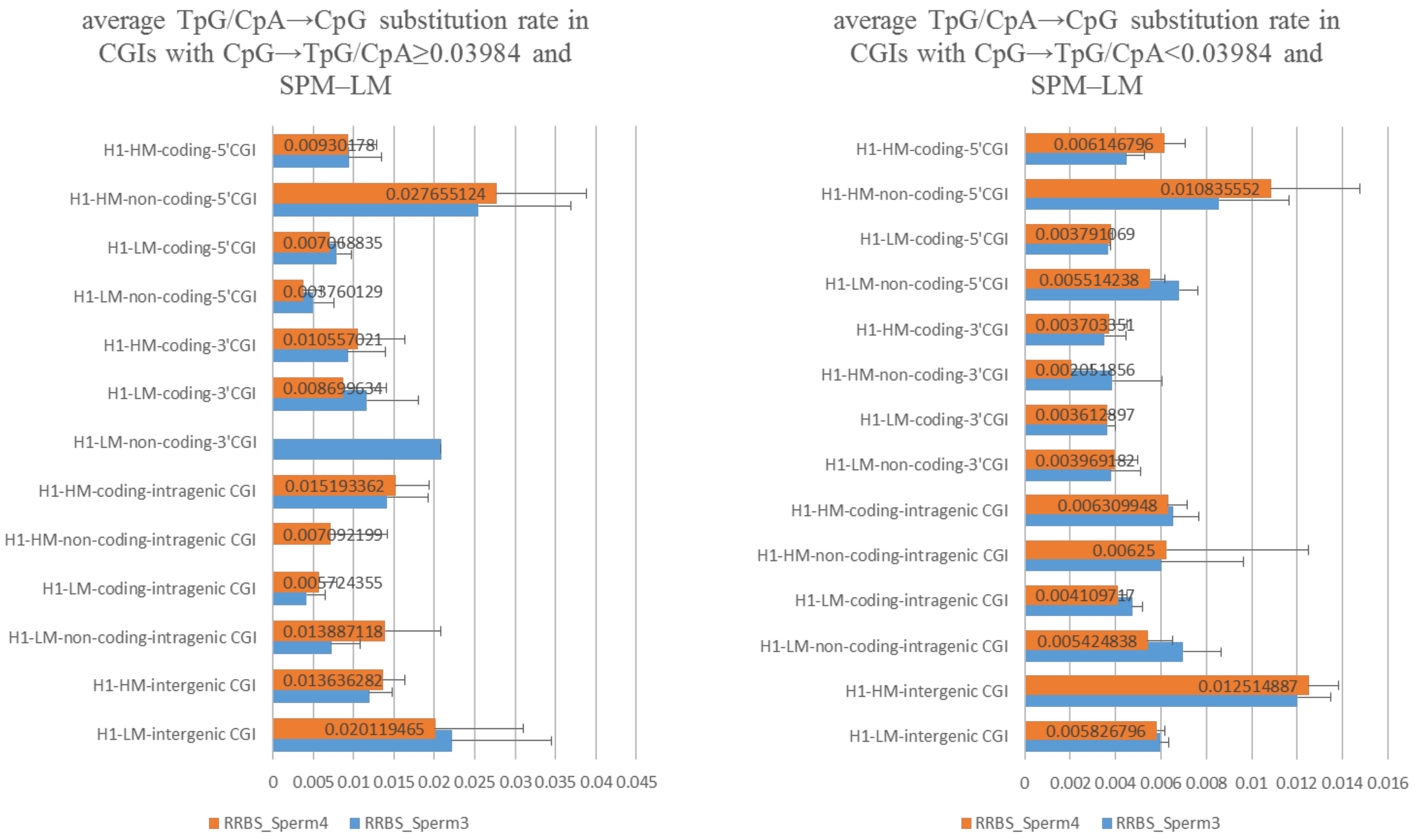

average TpG/CpA→CpG substitution rate in CGIs with CpG→TpG/CpA≥0.03984 and SPM–LM
H1-HM-coding-5'CGI
H1-HM-non-coding-5'CGI
H1-LM-coding-5'CGI
H1-LM-non-coding-5'CGI
H1-HM-coding-3'CGI
H1-LM-coding-3'CGI
H1-LM-non-coding-3'CGI
H1-HM-coding-intragenic CGI
H1-HM-non-coding-intragenic CGI
H1-LM-coding-intragenic CGI
H1-LM-non-coding-intragenic CGI
H1-HM-intergenic CGI
H1-LM-intergenic CGI
0.00930178
0.027655124
0.007068835
0.003760129
0.010557021
0.008699634
0.015193362
0.007092199
0.005724355
0.013887118
0.013636282
0.020119465
0 0.005 0.01 0.015 0.02 0.025 0.03 0.035 0.04 0.045
RRBS_Sperm4
RRBS_Sperm3
average TpG/CpA→CpG substitution rate in CGIs with CpG→TpG/CpA<0.03984 and SPM–LM
H1-HM-coding-5'CGI
H1-HM-non-coding-5'CGI
H1-LM-coding-5'CGI
H1-LM-non-coding-5'CGI
H1-HM-coding-3'CGI
H1-HM-non-coding-3'CGI
H1-LM-coding-3'CGI
H1-LM-non-coding-3'CGI
H1-HM-coding-intragenic CGI
H1-HM-non-coding-intragenic CGI
H1-LM-coding-intragenic CGI
H1-LM-non-coding-intragenic CGI
H1-HM-intergenic CGI
H1-LM-intergenic CGI
0.006146796
0.010835552
0.003791069
0.005514238
0.003703351
0.002051856
0.003612897
0.003969182
0.006309948
0.00625
0.004109717
0.005424838
0.012514887
0.005826796
0 0.002 0.004 0.006 0.008 0.01 0.012 0.014 0.016
RRBS_Sperm4
RRBS_Sperm3

C

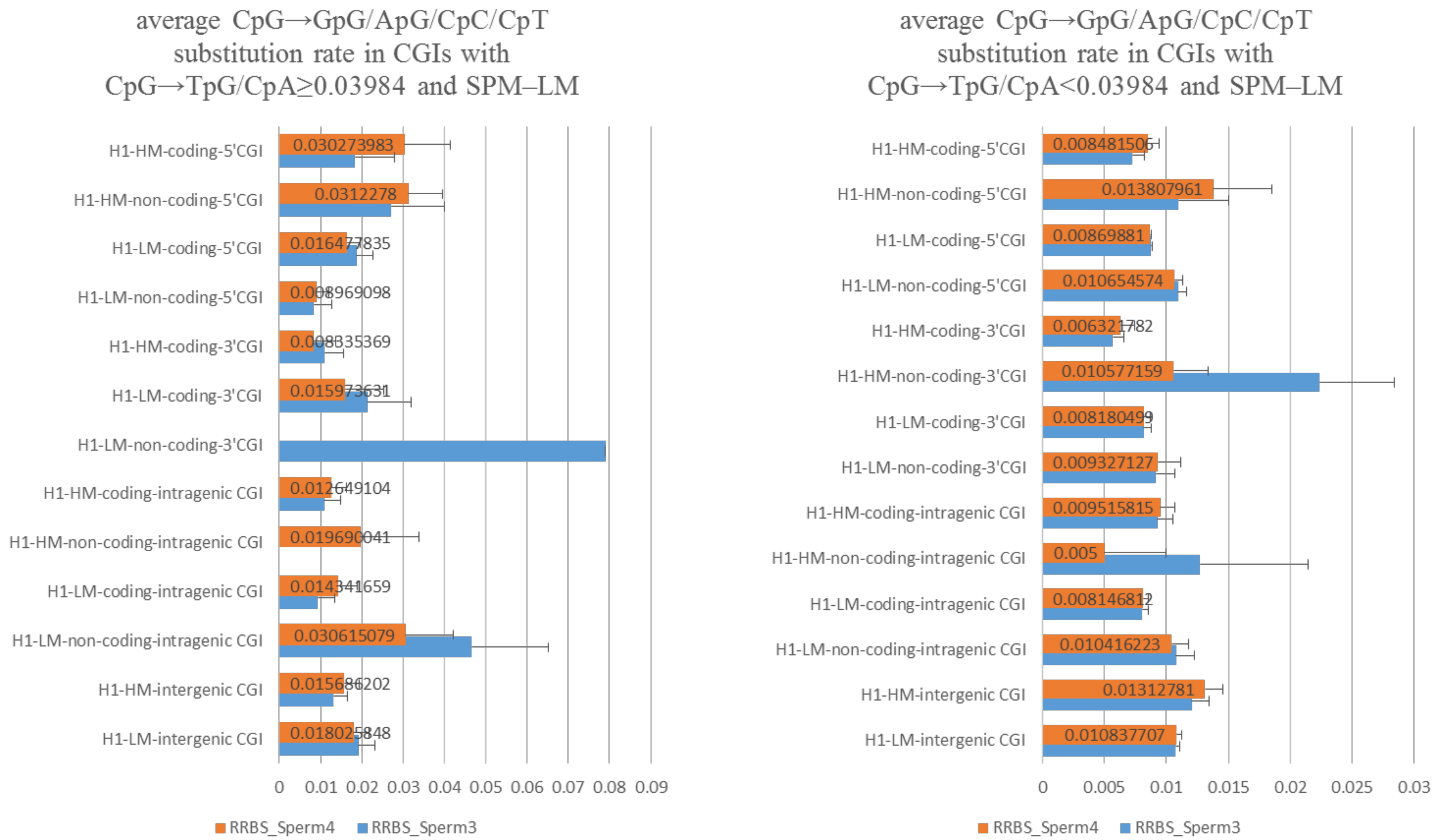
average CpG→GpG/ApG/CpC/CpT substitution rate in CGIs with CpG→TpG/CpA≥0.03984 and SPM–LM
H1-HM-coding-5'CGI
H1-HM-non-coding-5'CGI
H1-LM-coding-5'CGI
H1-LM-non-coding-5'CGI
H1-HM-coding-3'CGI
H1-LM-coding-3'CGI
H1-LM-non-coding-3'CGI
H1-HM-coding-intragenic CGI
H1-HM-non-coding-intragenic CGI
H1-LM-coding-intragenic CGI
H1-LM-non-coding-intragenic CGI
H1-HM-intergenic CGI
H1-LM-intergenic CGI
0.030273983
0.0312278
0.016477835
0.008969098
0.008335369
0.015973631
0.012649104
0.019690041
0.014341659
0.030615079
0.015686202
0.018025848
0
0.01
0.02
0.03
0.04
0.05
0.06
0.07
0.08
0.09
RRBS_Sperm4
RRBS_Sperm3
average CpG→GpG/ApG/CpC/CpT substitution rate in CGIs with CpG→TpG/CpA<0.03984 and SPM–LM
H1-HM-coding-5'CGI
H1-HM-non-coding-5'CGI
H1-LM-coding-5'CGI
H1-LM-non-coding-5'CGI
H1-HM-coding-3'CGI
H1-HM-non-coding-3'CGI
H1-LM-coding-3'CGI
H1-LM-non-coding-3'CGI
H1-HM-coding-intragenic CGI
H1-HM-non-coding-intragenic CGI
H1-LM-coding-intragenic CGI
H1-LM-non-coding-intragenic CGI
H1-HM-intergenic CGI
H1-LM-intergenic CGI
0.008481506
0.013807961
0.00869881
0.010654574
0.006321782
0.010577159
0.008180499
0.009327127
0.009515815
0.005
0.008146812
0.010416223
0.01312781
0.010837707
0
0.005
0.01
0.015
0.02
0.025
0.03
RRBS_Sperm4
RRBS_Sperm3

D

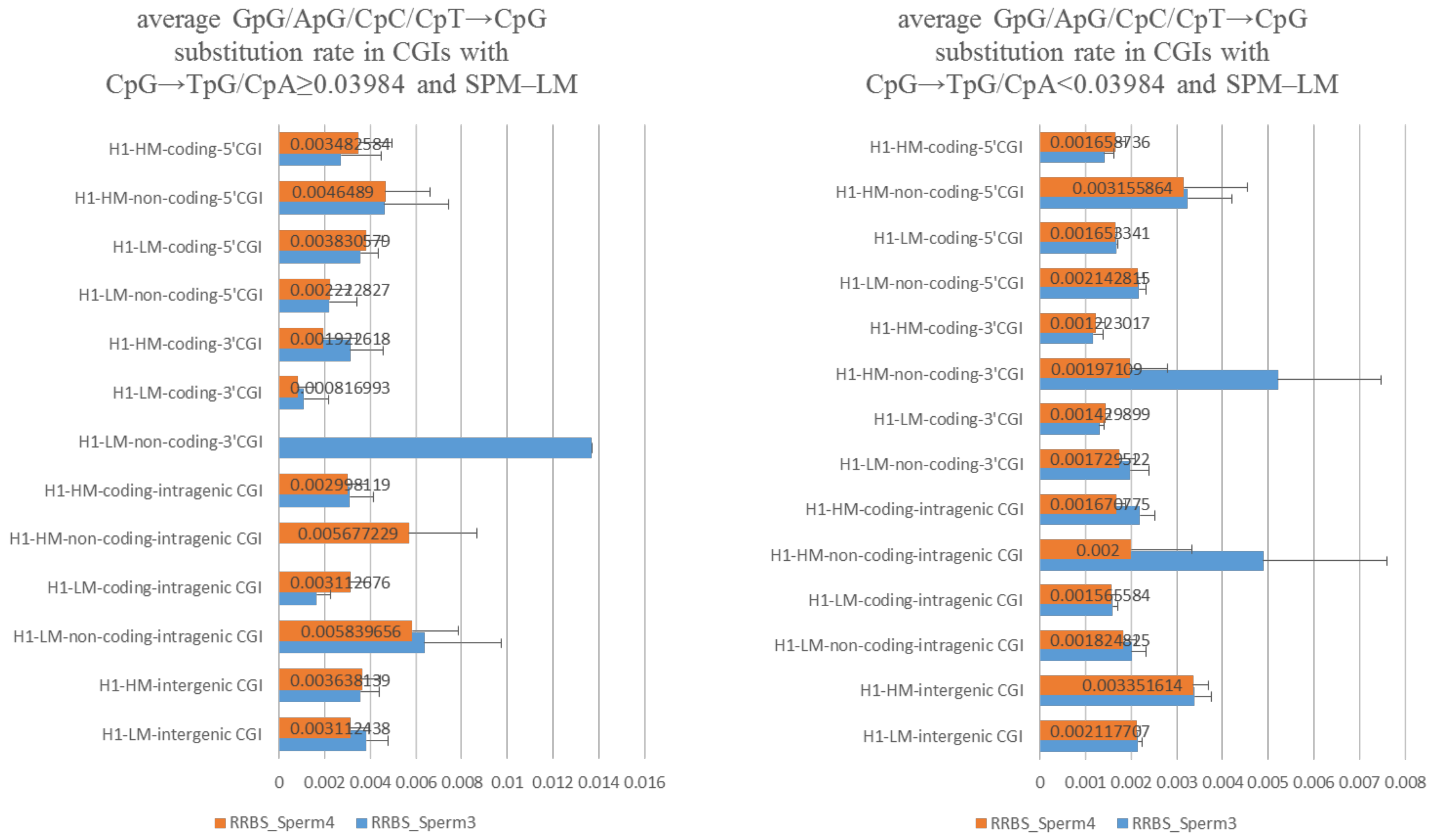
average GpG/ApG/CpC/CpT→CpG substitution rate in CGIs with CpG→TpG/CpA≥0.03984 and SPM–LM
H1-HM-coding-5'CGI
H1-HM-non-coding-5'CGI
H1-LM-coding-5'CGI
H1-LM-non-coding-5'CGI
H1-HM-coding-3'CGI
H1-LM-coding-3'CGI
H1-LM-non-coding-3'CGI
H1-HM-coding-intragenic CGI
H1-HM-non-coding-intragenic CGI
H1-LM-coding-intragenic CGI
H1-LM-non-coding-intragenic CGI
H1-HM-intergenic CGI
H1-LM-intergenic CGI
0.0046489
0.005677229
0.005839656
0 0.002 0.004 0.006 0.008 0.01 0.012 0.014 0.016
RRBS_Sperm4 RRBS_Sperm3
average GpG/ApG/CpC/CpT→CpG substitution rate in CGIs with CpG→TpG/CpA<0.03984 and SPM–LM
H1-HM-coding-5'CGI
H1-HM-non-coding-5'CGI
H1-LM-coding-5'CGI
H1-LM-non-coding-5'CGI
H1-HM-coding-3'CGI
H1-HM-non-coding-3'CGI
H1-LM-coding-3'CGI
H1-LM-non-coding-3'CGI
H1-HM-coding-intragenic CGI
H1-HM-non-coding-intragenic CGI
H1-LM-coding-intragenic CGI
H1-LM-non-coding-intragenic CGI
H1-HM-intergenic CGI
H1-LM-intergenic CGI
0.003155864
0.002
0.003351614
0 0.001 0.002 0.003 0.004 0.005 0.006 0.007 0.008
RRBS_Sperm4 RRBS_Sperm3

E

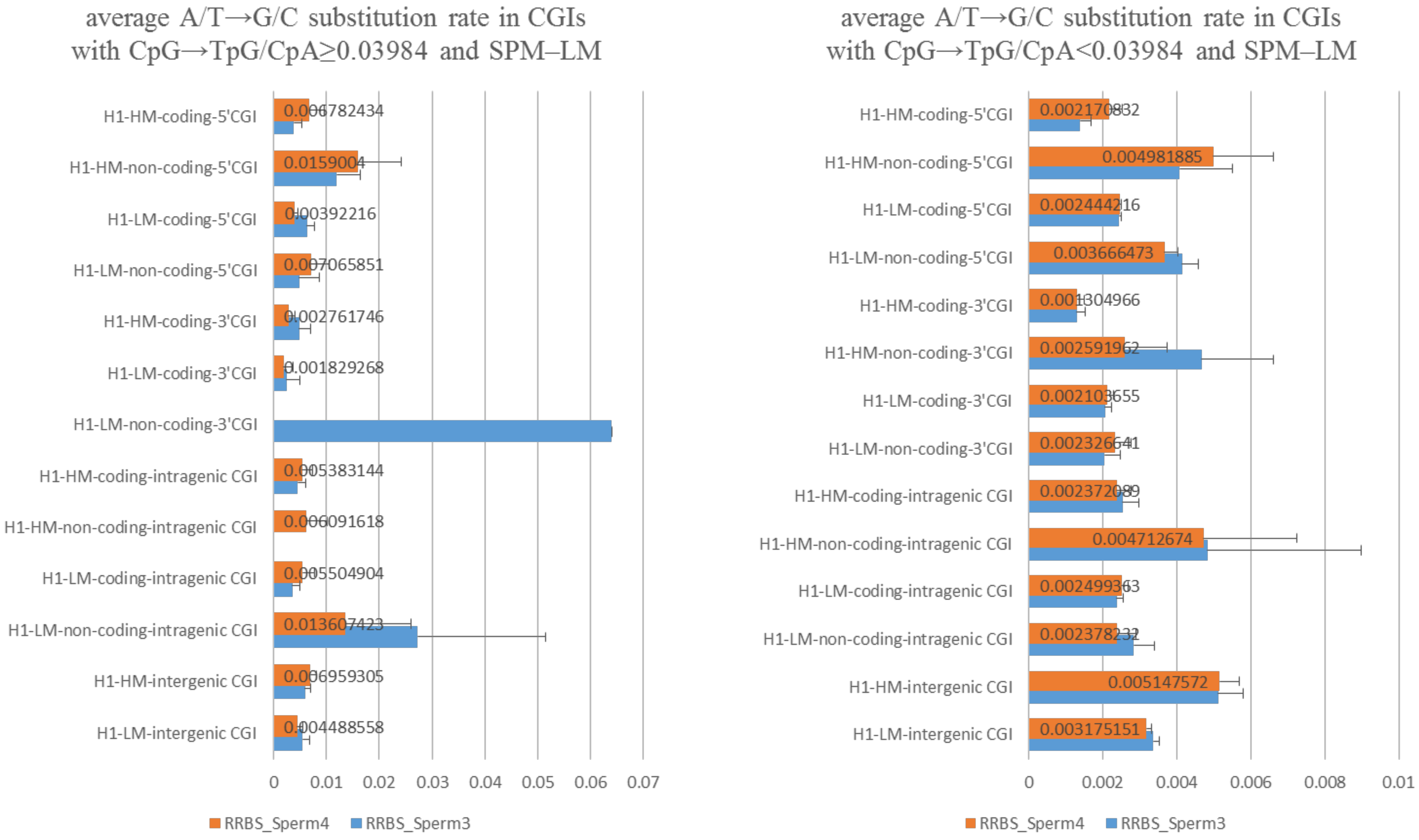
average A/T→G/C substitution rate in CGIs
with CpG→TpG/CpA≥0.03984 and SPM–LM
H1-HM-coding-5'CGI
H1-HM-non-coding-5'CGI
H1-LM-coding-5'CGI
H1-LM-non-coding-5'CGI
H1-HM-coding-3'CGI
H1-LM-coding-3'CGI
H1-LM-non-coding-3'CGI
H1-HM-coding-intragenic CGI
H1-HM-non-coding-intragenic CGI
H1-LM-coding-intragenic CGI
H1-LM-non-coding-intragenic CGI
H1-HM-intergenic CGI
H1-LM-intergenic CGI
0.006782434
0.0159004
0.00392216
0.007065851
0.002761746
0.001829268
0.005383144
0.006091618
0.005504904
0.013607423
0.006959305
0.004488558
0
0.01
0.02
0.03
0.04
0.05
0.06
0.07
RRBS_Sperm4
RRBS_Sperm3
average A/T→G/C substitution rate in CGIs
with CpG→TpG/CpA<0.03984 and SPM–LM
H1-HM-coding-5'CGI
H1-HM-non-coding-5'CGI
H1-LM-coding-5'CGI
H1-LM-non-coding-5'CGI
H1-HM-coding-3'CGI
H1-HM-non-coding-3'CGI
H1-LM-coding-3'CGI
H1-LM-non-coding-3'CGI
H1-HM-coding-intragenic CGI
H1-HM-non-coding-intragenic CGI
H1-LM-coding-intragenic CGI
H1-LM-non-coding-intragenic CGI
H1-HM-intergenic CGI
H1-LM-intergenic CGI
0.002170832
0.004981885
0.002444216
0.003666473
0.001304966
0.002591962
0.002103655
0.002326641
0.002372089
0.004712674
0.002499363
0.002378232
0.005147572
0.003175151
0
0.002
0.004
0.006
0.008
0.01
RRBS_Sperm4
RRBS_Sperm3

F

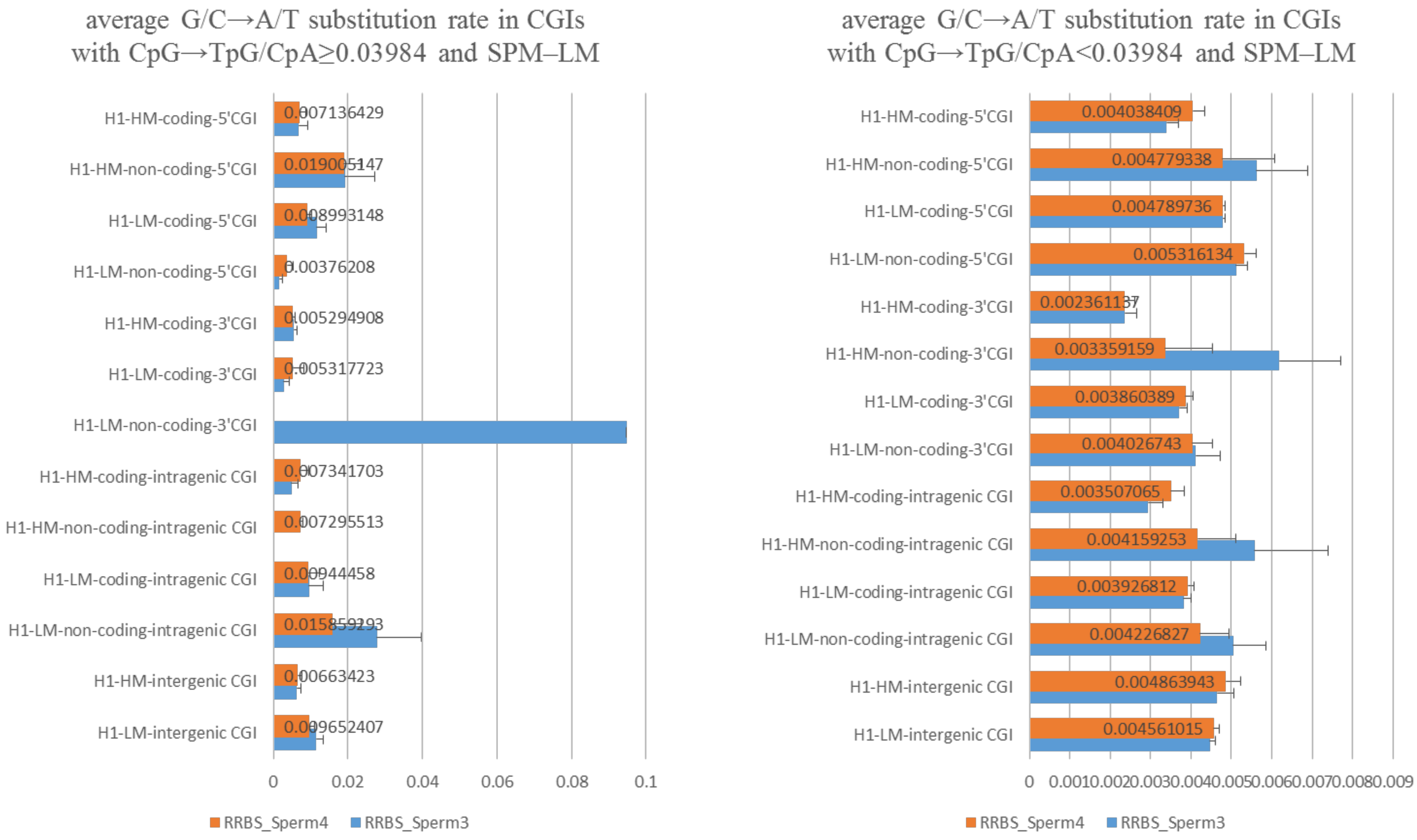

average G/C→A/T substitution rate in CGIs with CpG→TpG/CpA≥0.03984 and SPM–LM
H1-HM-coding-5'CGI
H1-HM-non-coding-5'CGI
H1-LM-coding-5'CGI
H1-LM-non-coding-5'CGI
H1-HM-coding-3'CGI
H1-LM-coding-3'CGI
H1-LM-non-coding-3'CGI
H1-HM-coding-intragenic CGI
H1-HM-non-coding-intragenic CGI
H1-LM-coding-intragenic CGI
H1-LM-non-coding-intragenic CGI
H1-HM-intergenic CGI
H1-LM-intergenic CGI
0.007136429
0.019005147
0.008993148
0.00376208
0.005294908
0.005317723
0.007341703
0.007295513
0.00944458
0.015859293
0.00663423
0.009652407
0
0.02
0.04
0.06
0.08
0.1
RRBS_Sperm4
RRBS_Sperm3
average G/C→A/T substitution rate in CGIs with CpG→TpG/CpA<0.03984 and SPM–LM
0.004038409
0.004779338
0.004789736
0.005316134
0.00236113
0.003359159
0.003860389
0.004026743
0.003507065
0.004159253
0.003926812
0.004226827
0.004863943
0.004561015
0 0.0010.0020.0030.0040.0050.0060.0070.0080.009
RRBS_Sperm4
RRBS_Sperm3
