# Supplementary material for: Factors to preserve CpG-rich sequences in methylated CpG islands
Source: BMC Genomics. 2015 Feb 28;16(1):144. doi: 10.1186/s12864-015-1286-x (PMC4417305; doi:10.1186/s12864-015-1286-x)
Supplement: Additional file 8: Figure S3. — Comparison of base substitution rates between CGIs with SPM-LM and SPM-HM. The details of the figure are the same as those described in the legend to Additional file 5: Figure S2. [file 12864_2015_1286_MOESM8_ESM.pdf]

A

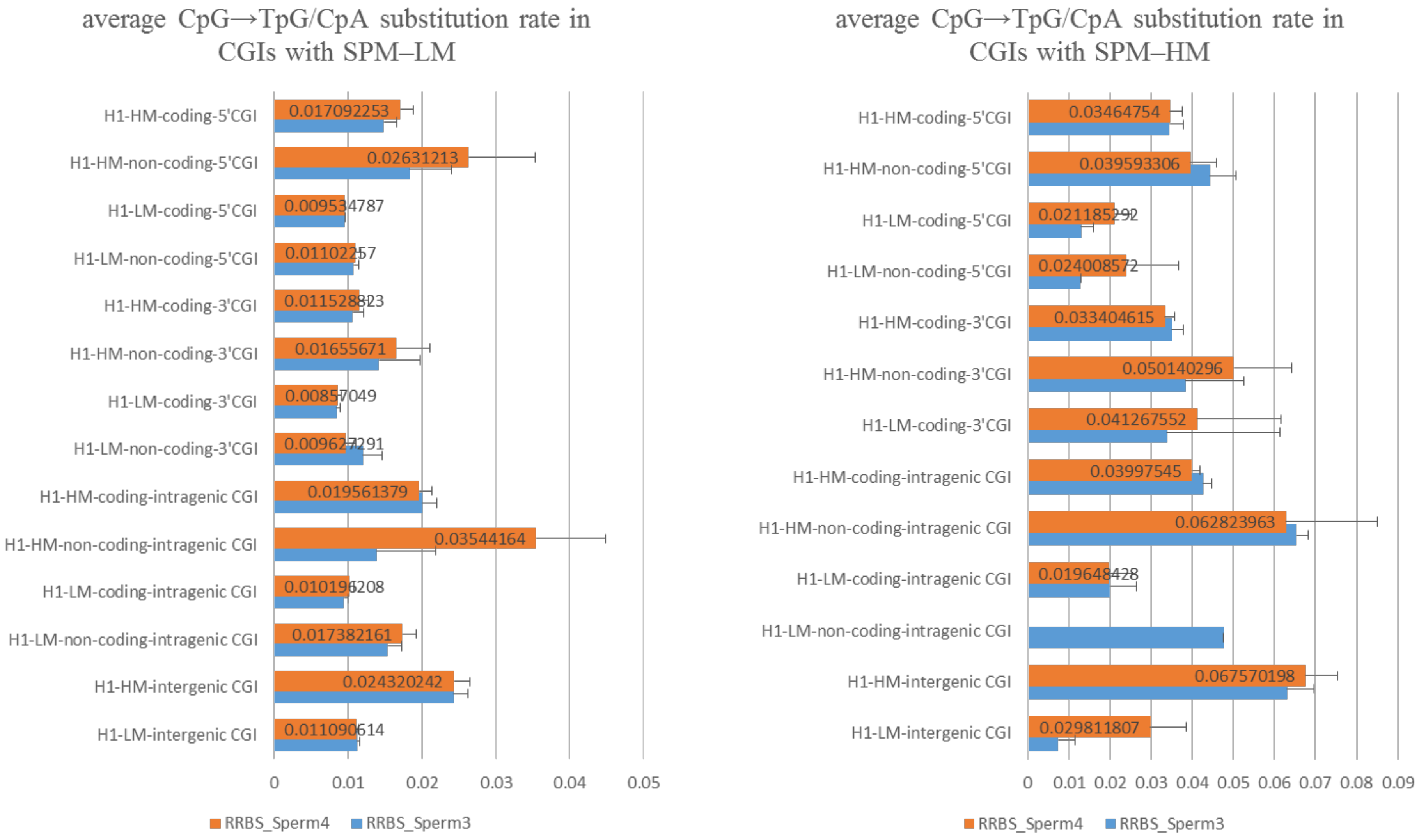

average CpG→TpG/CpA substitution rate in CGIs with SPM–LM
H1-HM-coding-5'CGI
H1-HM-non-coding-5'CGI
H1-LM-coding-5'CGI
H1-LM-non-coding-5'CGI
H1-HM-coding-3'CGI
H1-HM-non-coding-3'CGI
H1-LM-coding-3'CGI
H1-LM-non-coding-3'CGI
H1-HM-coding-intragenic CGI
H1-HM-non-coding-intragenic CGI
H1-LM-coding-intragenic CGI
H1-LM-non-coding-intragenic CGI
H1-HM-intergenic CGI
H1-LM-intergenic CGI
0.017092253
0.02631213
0.009534787
0.01102257
0.011528823
0.01655671
0.00857049
0.009627291
0.019561379
0.03544164
0.010196208
0.017382161
0.024320242
0.011090614
0
0.01
0.02
0.03
0.04
0.05
RRBS_Sperm4
RRBS_Sperm3
average CpG→TpG/CpA substitution rate in CGIs with SPM–HM
H1-HM-coding-5'CGI
H1-HM-non-coding-5'CGI
H1-LM-coding-5'CGI
H1-LM-non-coding-5'CGI
H1-HM-coding-3'CGI
H1-HM-non-coding-3'CGI
H1-LM-coding-3'CGI
H1-HM-coding-intragenic CGI
H1-HM-non-coding-intragenic CGI
H1-LM-coding-intragenic CGI
H1-LM-non-coding-intragenic CGI
H1-HM-intergenic CGI
H1-LM-intergenic CGI
0.03464754
0.039593306
0.021185292
0.024008572
0.033404615
0.050140296
0.041267552
0.03997545
0.062823963
0.019648428
0.067570198
0.029811807
0
0.01
0.02
0.03
0.04
0.05
0.06
0.07
0.08
0.09
RRBS_Sperm4
RRBS_Sperm3

B

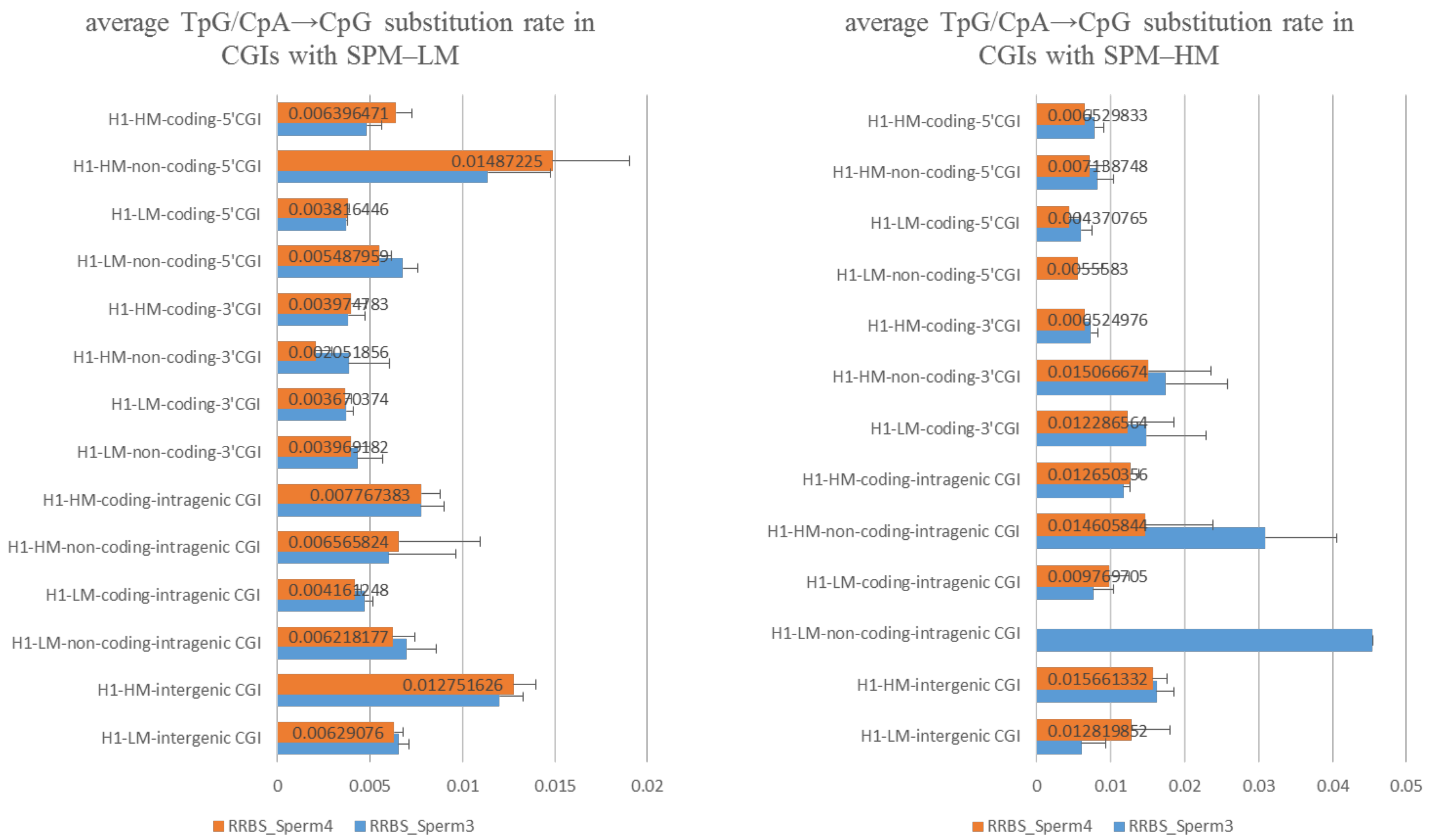

average TpG/CpA→CpG substitution rate in CGIs with SPM–LM
H1-HM-coding-5'CGI
H1-HM-non-coding-5'CGI
H1-LM-coding-5'CGI
H1-LM-non-coding-5'CGI
H1-HM-coding-3'CGI
H1-HM-non-coding-3'CGI
H1-LM-coding-3'CGI
H1-LM-non-coding-3'CGI
H1-HM-coding-intragenic CGI
H1-HM-non-coding-intragenic CGI
H1-LM-coding-intragenic CGI
H1-LM-non-coding-intragenic CGI
H1-HM-intergenic CGI
H1-LM-intergenic CGI
0.006396471
0.01487225
0.003816446
0.005487959
0.003974783
0.002051856
0.003670374
0.003969182
0.007767383
0.006565824
0.004161248
0.006218177
0.012751626
0.00629076
0
0.005
0.01
0.015
0.02
RRBS_Sperm4
RRBS_Sperm3
average TpG/CpA→CpG substitution rate in CGIs with SPM–HM
H1-HM-coding-5'CGI
H1-HM-non-coding-5'CGI
H1-LM-coding-5'CGI
H1-LM-non-coding-5'CGI
H1-HM-coding-3'CGI
H1-HM-non-coding-3'CGI
H1-LM-coding-3'CGI
H1-HM-coding-intragenic CGI
H1-HM-non-coding-intragenic CGI
H1-LM-coding-intragenic CGI
H1-LM-non-coding-intragenic CGI
H1-HM-intergenic CGI
H1-LM-intergenic CGI
0.006529833
0.007138748
0.004370765
0.0055583
0.006524976
0.015066674
0.012286564
0.012650356
0.014605844
0.009769705
0.015661332
0.012819852
0
0.01
0.02
0.03
0.04
0.05
RRBS_Sperm4
RRBS_Sperm3

C

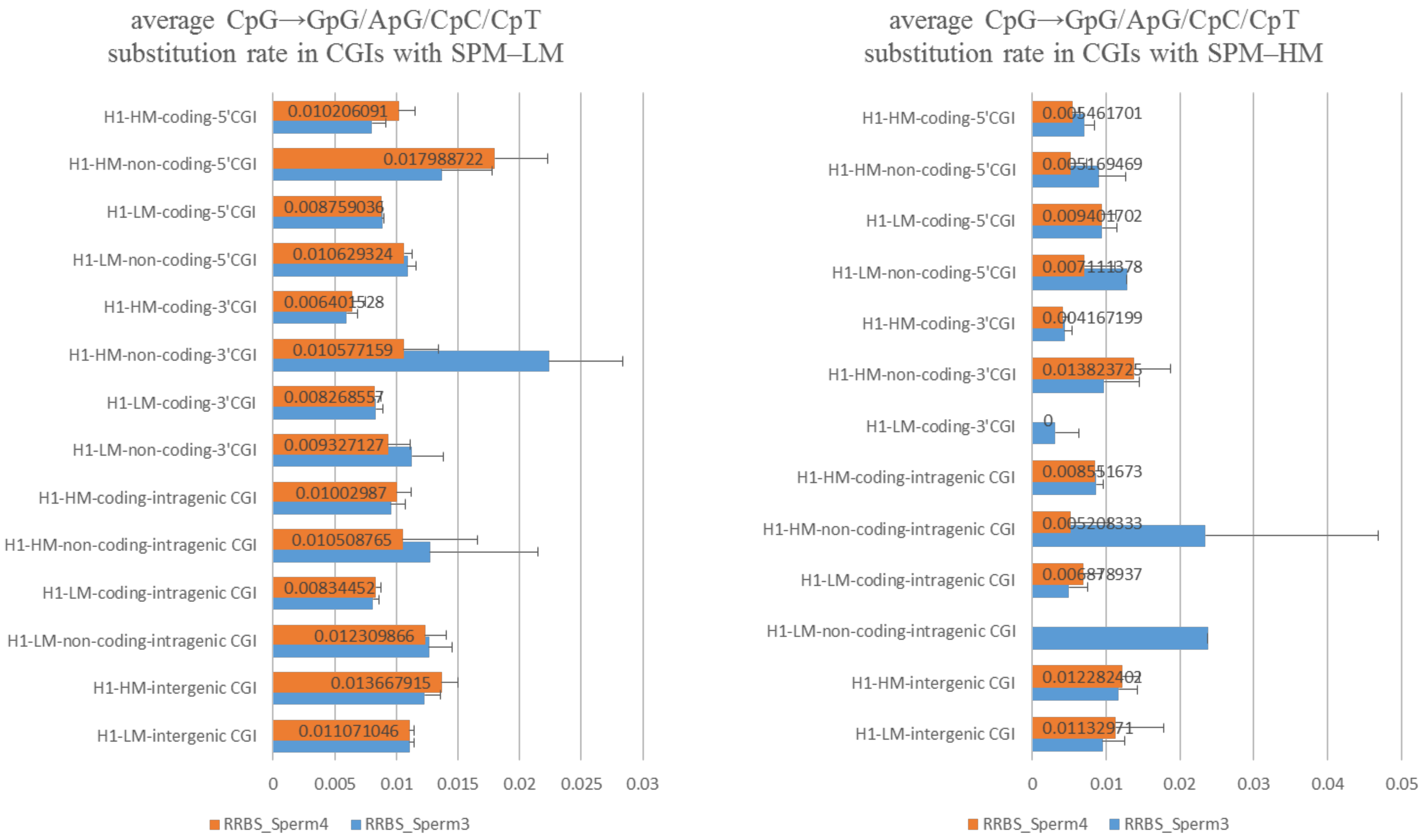
average CpG→GpG/ApG/CpC/CpT substitution rate in CGIs with SPM–LM
H1-HM-coding-5'CGI
H1-HM-non-coding-5'CGI
H1-LM-coding-5'CGI
H1-LM-non-coding-5'CGI
H1-HM-coding-3'CGI
H1-HM-non-coding-3'CGI
H1-LM-coding-3'CGI
H1-LM-non-coding-3'CGI
H1-HM-coding-intragenic CGI
H1-HM-non-coding-intragenic CGI
H1-LM-coding-intragenic CGI
H1-LM-non-coding-intragenic CGI
H1-HM-intergenic CGI
H1-LM-intergenic CGI
0.010206091
0.017988722
0.008759036
0.010629324
0.006401528
0.010577159
0.008268557
0.009327127
0.01002987
0.010508765
0.00834452
0.012309866
0.013667915
0.011071046
0
0.005
0.01
0.015
0.02
0.025
0.03
RRBS_Sperm4
RRBS_Sperm3
average CpG→GpG/ApG/CpC/CpT substitution rate in CGIs with SPM–HM
H1-HM-coding-5'CGI
H1-HM-non-coding-5'CGI
H1-LM-coding-5'CGI
H1-LM-non-coding-5'CGI
H1-HM-coding-3'CGI
H1-HM-non-coding-3'CGI
H1-LM-coding-3'CGI
H1-HM-coding-intragenic CGI
H1-HM-non-coding-intragenic CGI
H1-LM-coding-intragenic CGI
H1-LM-non-coding-intragenic CGI
H1-HM-intergenic CGI
H1-LM-intergenic CGI
0.005461701
0.005169469
0.009401702
0.007111378
0.004167199
0.013823725
0
0.008551673
0.005208333
0.006878937
0.012282402
0.01132971
0
0.01
0.02
0.03
0.04
0.05
RRBS_Sperm4
RRBS_Sperm3

D

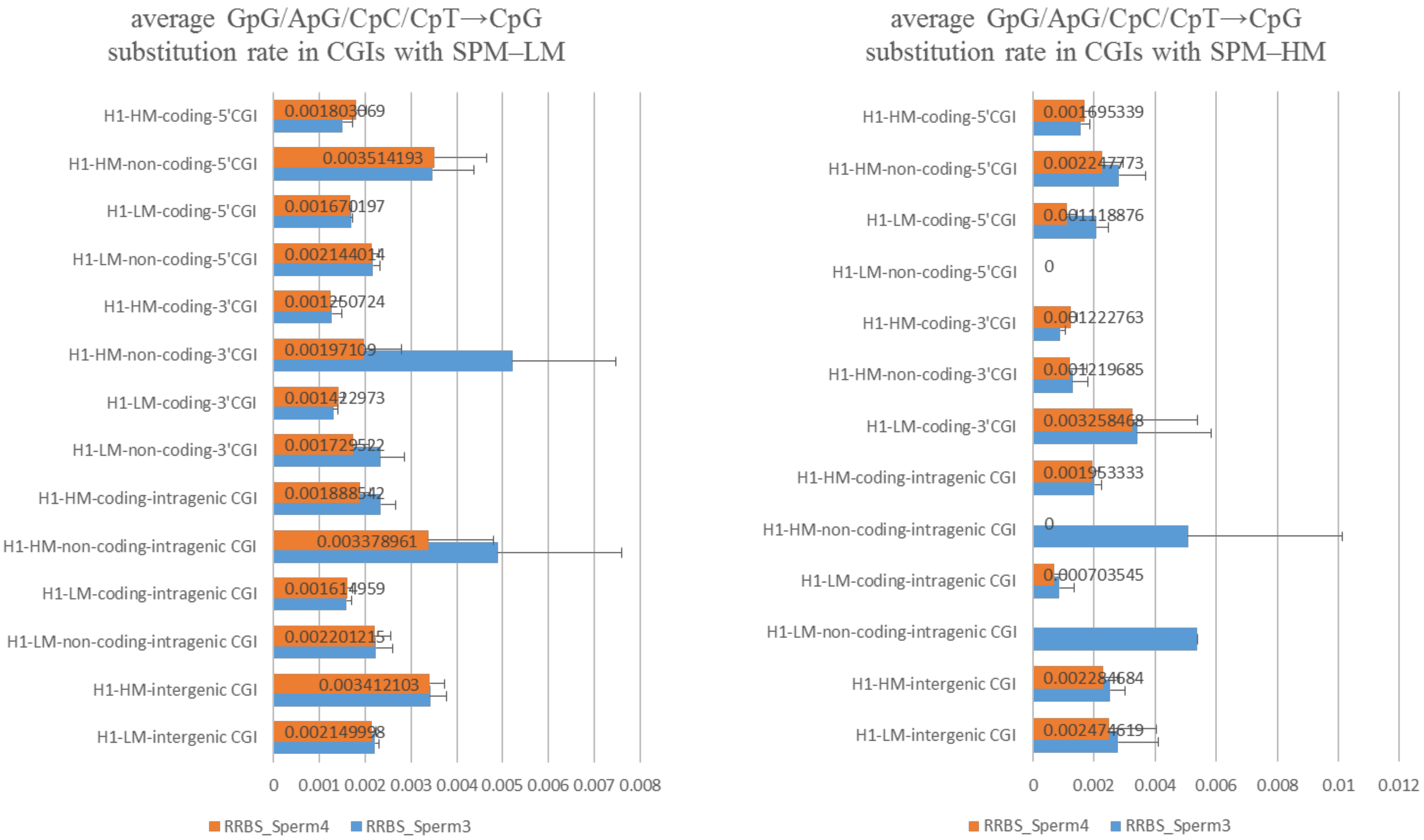
average GpG/ApG/CpC/CpT→CpG substitution rate in CGIs with SPM–LM
H1-HM-coding-5'CGI
H1-HM-non-coding-5'CGI
H1-LM-coding-5'CGI
H1-LM-non-coding-5'CGI
H1-HM-coding-3'CGI
H1-HM-non-coding-3'CGI
H1-LM-coding-3'CGI
H1-LM-non-coding-3'CGI
H1-HM-coding-intragenic CGI
H1-HM-non-coding-intragenic CGI
H1-LM-coding-intragenic CGI
H1-LM-non-coding-intragenic CGI
H1-HM-intergenic CGI
H1-LM-intergenic CGI
0.001803069
0.003514193
0.001670197
0.002144014
0.001250724
0.00197109
0.001422973
0.001729522
0.001888542
0.003378961
0.001614959
0.002201215
0.003412103
0.002149998
0 0.001 0.002 0.003 0.004 0.005 0.006 0.007 0.008
RRBS_Sperm4 RRBS_Sperm3
average GpG/ApG/CpC/CpT→CpG substitution rate in CGIs with SPM–HM
H1-HM-coding-5'CGI
H1-HM-non-coding-5'CGI
H1-LM-coding-5'CGI
H1-LM-non-coding-5'CGI
H1-HM-coding-3'CGI
H1-HM-non-coding-3'CGI
H1-LM-coding-3'CGI
H1-HM-coding-intragenic CGI
H1-HM-non-coding-intragenic CGI
H1-LM-coding-intragenic CGI
H1-LM-non-coding-intragenic CGI
H1-HM-intergenic CGI
H1-LM-intergenic CGI
0.001695339
0.002247773
0.001118876
0
0.001222763
0.001219685
0.003258468
0.001953333
0
0.000703545
0.002284684
0.002474619
0 0.002 0.004 0.006 0.008 0.01 0.012
RRBS_Sperm4 RRBS_Sperm3

E

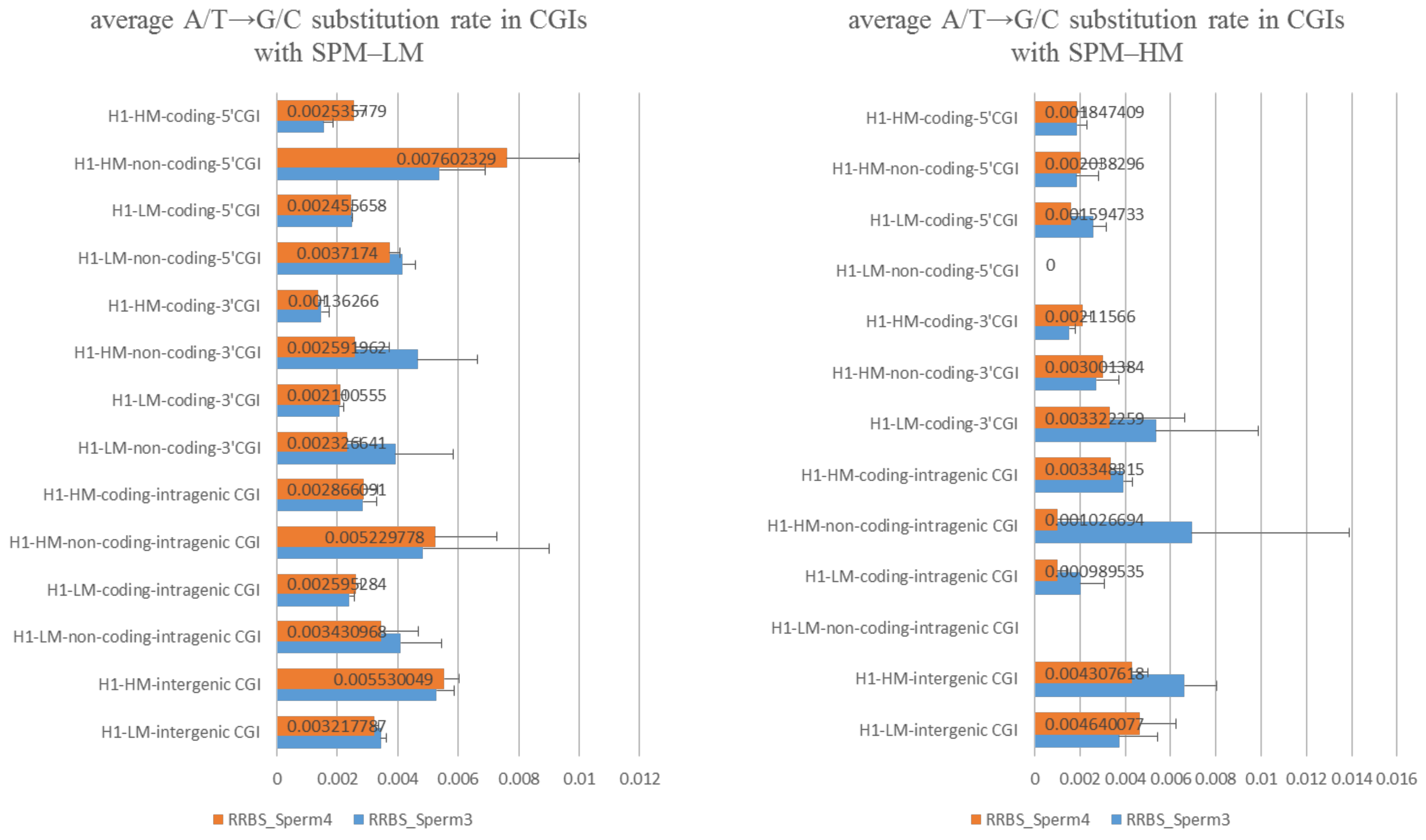

average A/T→G/C substitution rate in CGIs with SPM–LM
H1-HM-coding-5'CGI
H1-HM-non-coding-5'CGI
H1-LM-coding-5'CGI
H1-LM-non-coding-5'CGI
H1-HM-coding-3'CGI
H1-HM-non-coding-3'CGI
H1-LM-coding-3'CGI
H1-LM-non-coding-3'CGI
H1-HM-coding-intragenic CGI
H1-HM-non-coding-intragenic CGI
H1-LM-coding-intragenic CGI
H1-LM-non-coding-intragenic CGI
H1-HM-intergenic CGI
H1-LM-intergenic CGI
0.002535779
0.007602329
0.002455658
0.0037174
0.00136266
0.002591962
0.002100555
0.002326641
0.002866091
0.005229778
0.002595284
0.003430968
0.005530049
0.003217787
0
0.002
0.004
0.006
0.008
0.01
0.012
RRBS_Sperm4
RRBS_Sperm3
average A/T→G/C substitution rate in CGIs with SPM–HM
H1-HM-coding-5'CGI
H1-HM-non-coding-5'CGI
H1-LM-coding-5'CGI
H1-LM-non-coding-5'CGI
H1-HM-coding-3'CGI
H1-HM-non-coding-3'CGI
H1-LM-coding-3'CGI
H1-HM-coding-intragenic CGI
H1-HM-non-coding-intragenic CGI
H1-LM-coding-intragenic CGI
H1-LM-non-coding-intragenic CGI
H1-HM-intergenic CGI
H1-LM-intergenic CGI
0.001847409
0.002038296
0.001594733
0
0.00211566
0.003001384
0.003322259
0.003348315
0.001026694
0.000989535
0.004307618
0.004640077
0
0.002
0.004
0.006
0.008
0.01
0.012
0.014
0.016
RRBS_Sperm4
RRBS_Sperm3

F

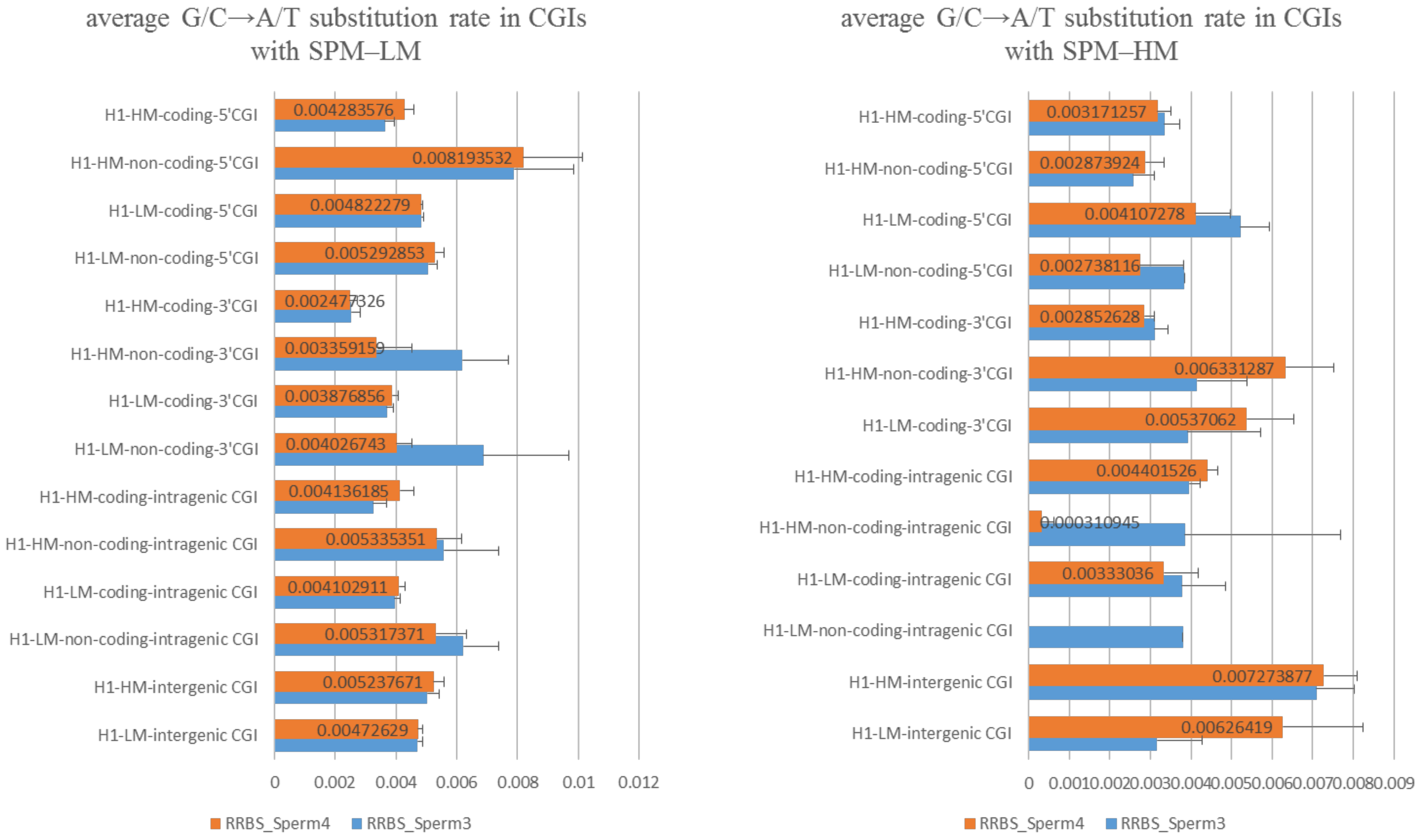

average G/C→A/T substitution rate in CGIs with SPM–LM
H1-HM-coding-5'CGI
H1-HM-non-coding-5'CGI
H1-LM-coding-5'CGI
H1-LM-non-coding-5'CGI
H1-HM-coding-3'CGI
H1-HM-non-coding-3'CGI
H1-LM-coding-3'CGI
H1-LM-non-coding-3'CGI
H1-HM-coding-intragenic CGI
H1-HM-non-coding-intragenic CGI
H1-LM-coding-intragenic CGI
H1-LM-non-coding-intragenic CGI
H1-HM-intergenic CGI
H1-LM-intergenic CGI
0.004283576
0.008193532
0.004822279
0.005292853
0.002477326
0.003359159
0.003876856
0.004026743
0.004136185
0.005335351
0.004102911
0.005317371
0.005237671
0.00472629
0
0.002
0.004
0.006
0.008
0.01
0.012
RRBS_Sperm4
RRBS_Sperm3
average G/C→A/T substitution rate in CGIs with SPM–HM
H1-HM-coding-5'CGI
H1-HM-non-coding-5'CGI
H1-LM-coding-5'CGI
H1-LM-non-coding-5'CGI
H1-HM-coding-3'CGI
H1-HM-non-coding-3'CGI
H1-LM-coding-3'CGI
H1-HM-coding-intragenic CGI
H1-HM-non-coding-intragenic CGI
H1-LM-coding-intragenic CGI
H1-LM-non-coding-intragenic CGI
H1-HM-intergenic CGI
H1-LM-intergenic CGI
0.003171257
0.002873924
0.004107278
0.002738116
0.002852628
0.006331287
0.00537062
0.004401526
0.000310945
0.00333036
0.007273877
0.00626419
0 0.0010.0020.0030.0040.0050.0060.0070.0080.009
RRBS_Sperm4
RRBS_Sperm3
