# Supplementary material for: Factors to preserve CpG-rich sequences in methylated CpG islands
Source: BMC Genomics. 2015 Feb 28;16(1):144. doi: 10.1186/s12864-015-1286-x (PMC4417305; doi:10.1186/s12864-015-1286-x)
Supplement: Additional file 10: Figure S4. — Comparison of base substitution rates between lowly methylated CGIs with CpG → TpG/CpA < 0.03984 and highly methylated CGIs with CpG → TpG/CpA ≥ 0.03984. The details of the figure are the same as those described in the legend to Additional file 5: Figure S2. [file 12864_2015_1286_MOESM10_ESM.pdf]

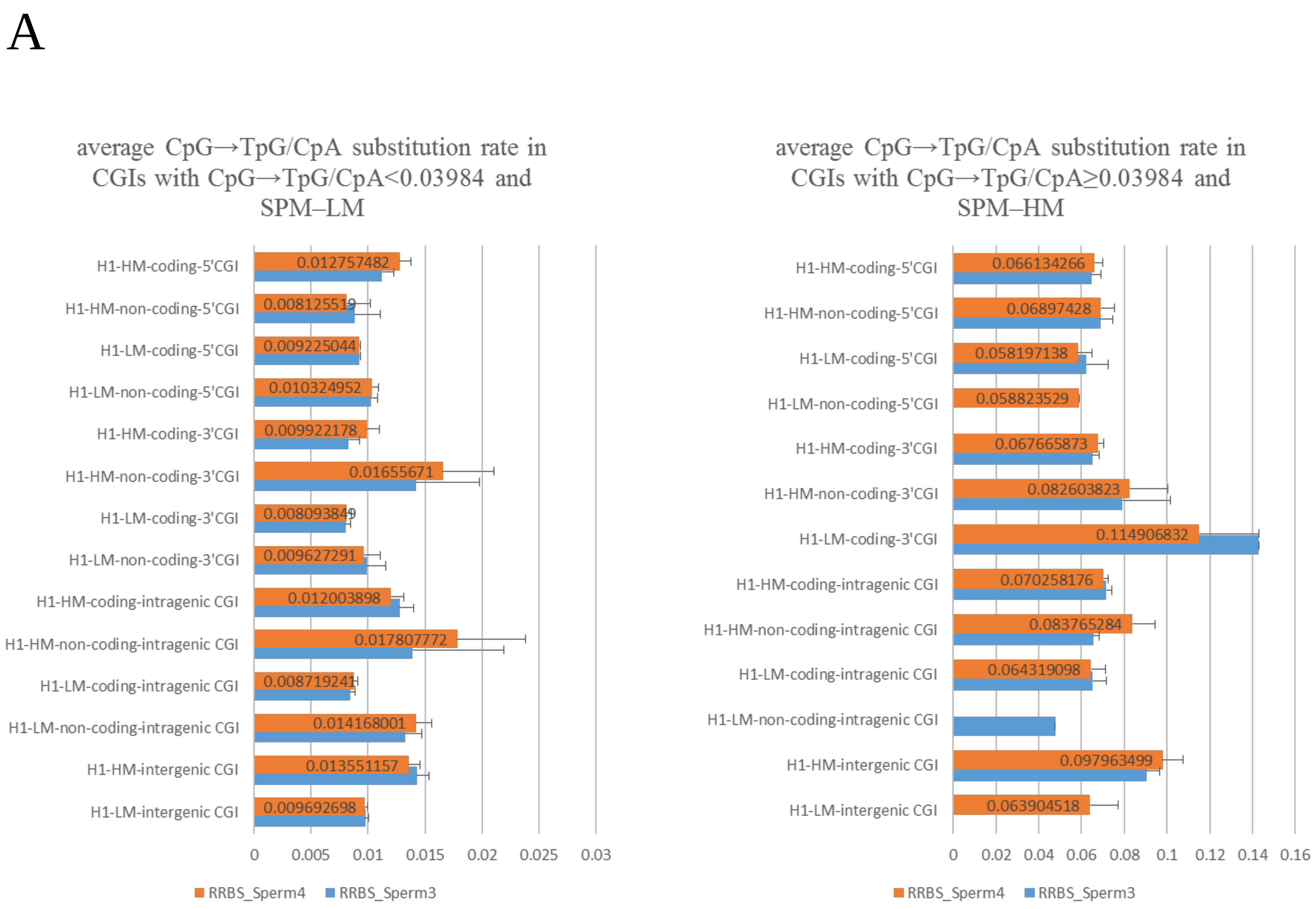
A
average CpG→TpG/CpA substitution rate in CGIs with CpG→TpG/CpA<0.03984 and SPM–LM
H1-HM-coding-5'CGI
H1-HM-non-coding-5'CGI
H1-LM-coding-5'CGI
H1-LM-non-coding-5'CGI
H1-HM-coding-3'CGI
H1-HM-non-coding-3'CGI
H1-LM-coding-3'CGI
H1-LM-non-coding-3'CGI
H1-HM-coding-intragenic CGI
H1-HM-non-coding-intragenic CGI
H1-LM-coding-intragenic CGI
H1-LM-non-coding-intragenic CGI
H1-HM-intergenic CGI
H1-LM-intergenic CGI
0.012757482
0.008125519
0.009225044
0.010324952
0.009922178
0.01655671
0.008093849
0.009627291
0.012003898
0.017807772
0.008719241
0.014168001
0.013551157
0.009692698
0
0.005
0.01
0.015
0.02
0.025
0.03
RRBS_Sperm4
RRBS_Sperm3
average CpG→TpG/CpA substitution rate in CGIs with CpG→TpG/CpA≥0.03984 and SPM–HM
H1-HM-coding-5'CGI
H1-HM-non-coding-5'CGI
H1-LM-coding-5'CGI
H1-LM-non-coding-5'CGI
H1-HM-coding-3'CGI
H1-HM-non-coding-3'CGI
H1-LM-coding-3'CGI
H1-HM-coding-intragenic CGI
H1-HM-non-coding-intragenic CGI
H1-LM-coding-intragenic CGI
H1-LM-non-coding-intragenic CGI
H1-HM-intergenic CGI
H1-LM-intergenic CGI
0.066134266
0.06897428
0.058197138
0.058823529
0.067665873
0.082603823
0.114906832
0.070258176
0.083765284
0.064319098
0.097963499
0.063904518
0
0.02
0.04
0.06
0.08
0.1
0.12
0.14
0.16
RRBS_Sperm4
RRBS_Sperm3

B

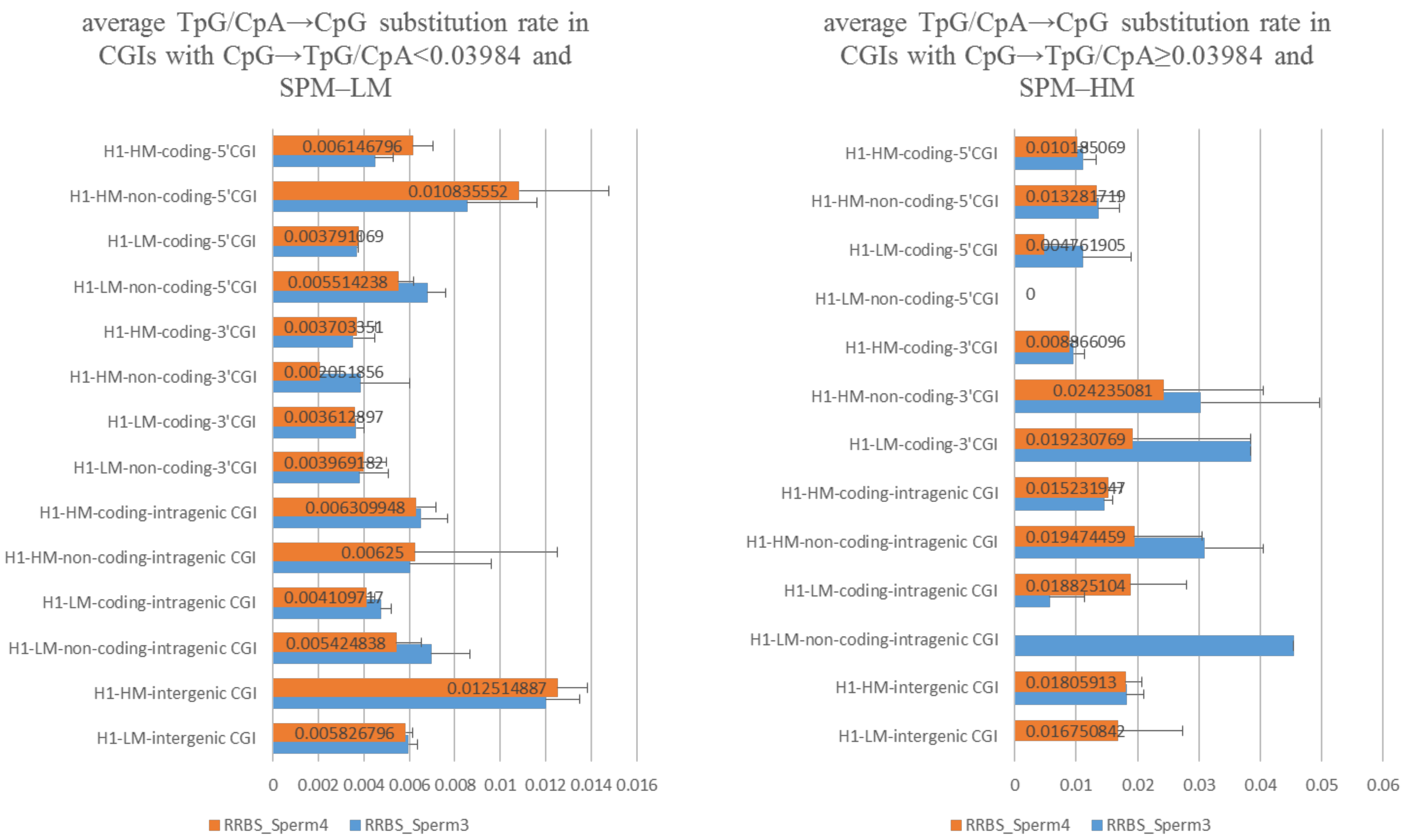

average TpG/CpA→CpG substitution rate in CGIs with CpG→TpG/CpA<0.03984 and SPM–LM
H1-HM-coding-5'CGI
H1-HM-non-coding-5'CGI
H1-LM-coding-5'CGI
H1-LM-non-coding-5'CGI
H1-HM-coding-3'CGI
H1-HM-non-coding-3'CGI
H1-LM-coding-3'CGI
H1-LM-non-coding-3'CGI
H1-HM-coding-intragenic CGI
H1-HM-non-coding-intragenic CGI
H1-LM-coding-intragenic CGI
H1-LM-non-coding-intragenic CGI
H1-HM-intergenic CGI
H1-LM-intergenic CGI
0.006146796
0.010835552
0.003791069
0.005514238
0.003703351
0.002051856
0.003612897
0.003969182
0.006309948
0.00625
0.004109717
0.005424838
0.012514887
0.005826796
0 0.002 0.004 0.006 0.008 0.01 0.012 0.014 0.016
RRBS_Sperm4 RRBS_Sperm3
average TpG/CpA→CpG substitution rate in CGIs with CpG→TpG/CpA≥0.03984 and SPM–HM
H1-HM-coding-5'CGI
H1-HM-non-coding-5'CGI
H1-LM-coding-5'CGI
H1-LM-non-coding-5'CGI
H1-HM-coding-3'CGI
H1-HM-non-coding-3'CGI
H1-LM-coding-3'CGI
H1-HM-coding-intragenic CGI
H1-HM-non-coding-intragenic CGI
H1-LM-coding-intragenic CGI
H1-LM-non-coding-intragenic CGI
H1-HM-intergenic CGI
H1-LM-intergenic CGI
0.010185069
0.013281719
0.004761905
0
0.008866096
0.024235081
0.019230769
0.015231947
0.019474459
0.018825104
0.01805913
0.016750842
0 0.01 0.02 0.03 0.04 0.05 0.06
RRBS_Sperm4 RRBS_Sperm3

C

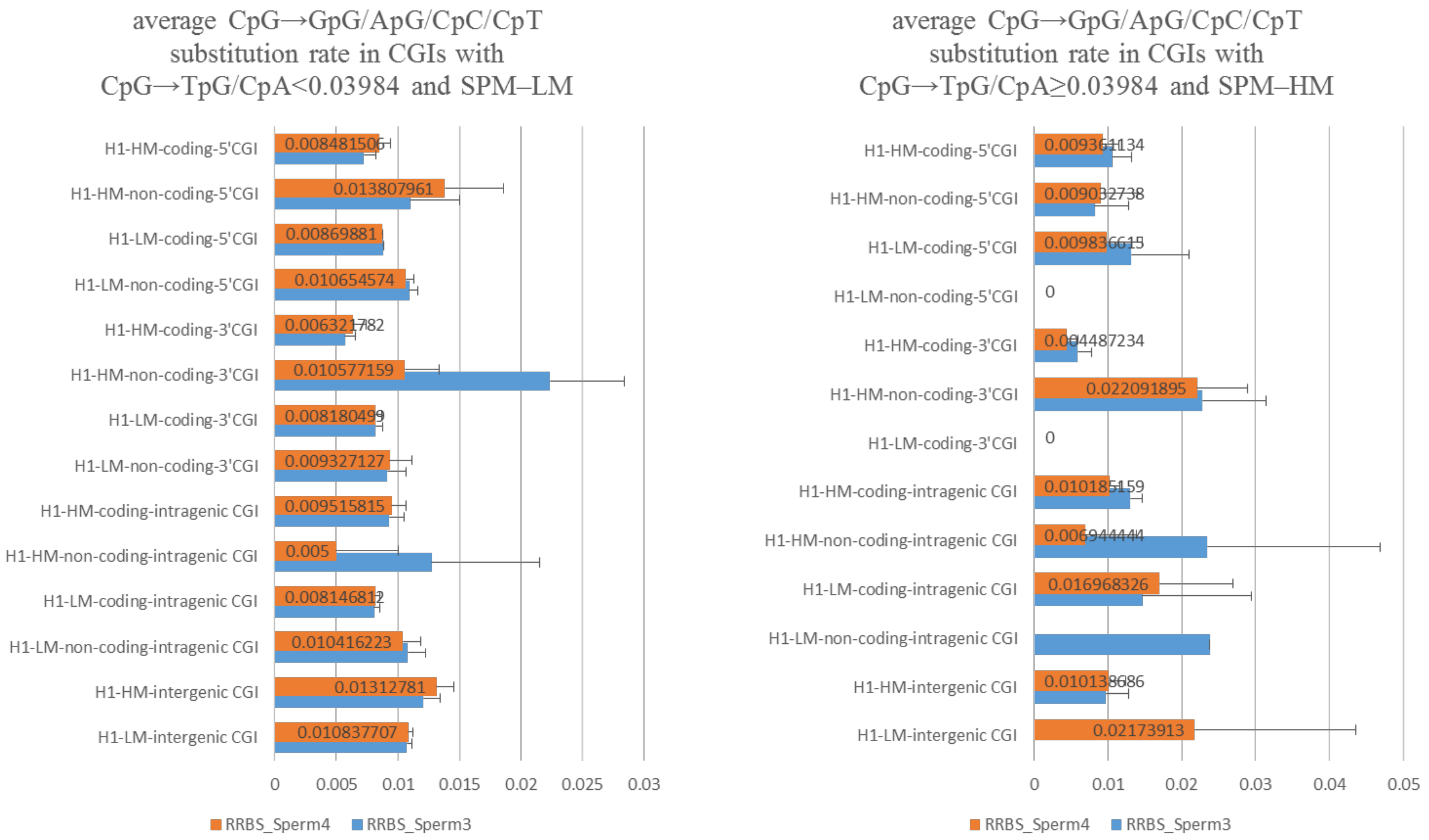
average CpG→GpG/ApG/CpC/CpT substitution rate in CGIs with CpG→TpG/CpA<0.03984 and SPM–LM
H1-HM-coding-5'CGI
H1-HM-non-coding-5'CGI
H1-LM-coding-5'CGI
H1-LM-non-coding-5'CGI
H1-HM-coding-3'CGI
H1-HM-non-coding-3'CGI
H1-LM-coding-3'CGI
H1-LM-non-coding-3'CGI
H1-HM-coding-intragenic CGI
H1-HM-non-coding-intragenic CGI
H1-LM-coding-intragenic CGI
H1-LM-non-coding-intragenic CGI
H1-HM-intergenic CGI
H1-LM-intergenic CGI
0.008481506
0.013807961
0.00869881
0.010654574
0.006321782
0.010577159
0.008180499
0.009327127
0.009515815
0.005
0.008146812
0.010416223
0.01312781
0.010837707
0
0.005
0.01
0.015
0.02
0.025
0.03
RRBS_Sperm4
RRBS_Sperm3
average CpG→GpG/ApG/CpC/CpT substitution rate in CGIs with CpG→TpG/CpA≥0.03984 and SPM–HM
H1-HM-coding-5'CGI
H1-HM-non-coding-5'CGI
H1-LM-coding-5'CGI
H1-LM-non-coding-5'CGI
H1-HM-coding-3'CGI
H1-HM-non-coding-3'CGI
H1-LM-coding-3'CGI
H1-HM-coding-intragenic CGI
H1-HM-non-coding-intragenic CGI
H1-LM-coding-intragenic CGI
H1-LM-non-coding-intragenic CGI
H1-HM-intergenic CGI
H1-LM-intergenic CGI
0.009361134
0.009032738
0.009836615
0
0.004487234
0.022091895
0
0.010185159
0.006944444
0.016968326
0.010138686
0.02173913
0
0.01
0.02
0.03
0.04
0.05
RRBS_Sperm4
RRBS_Sperm3

D

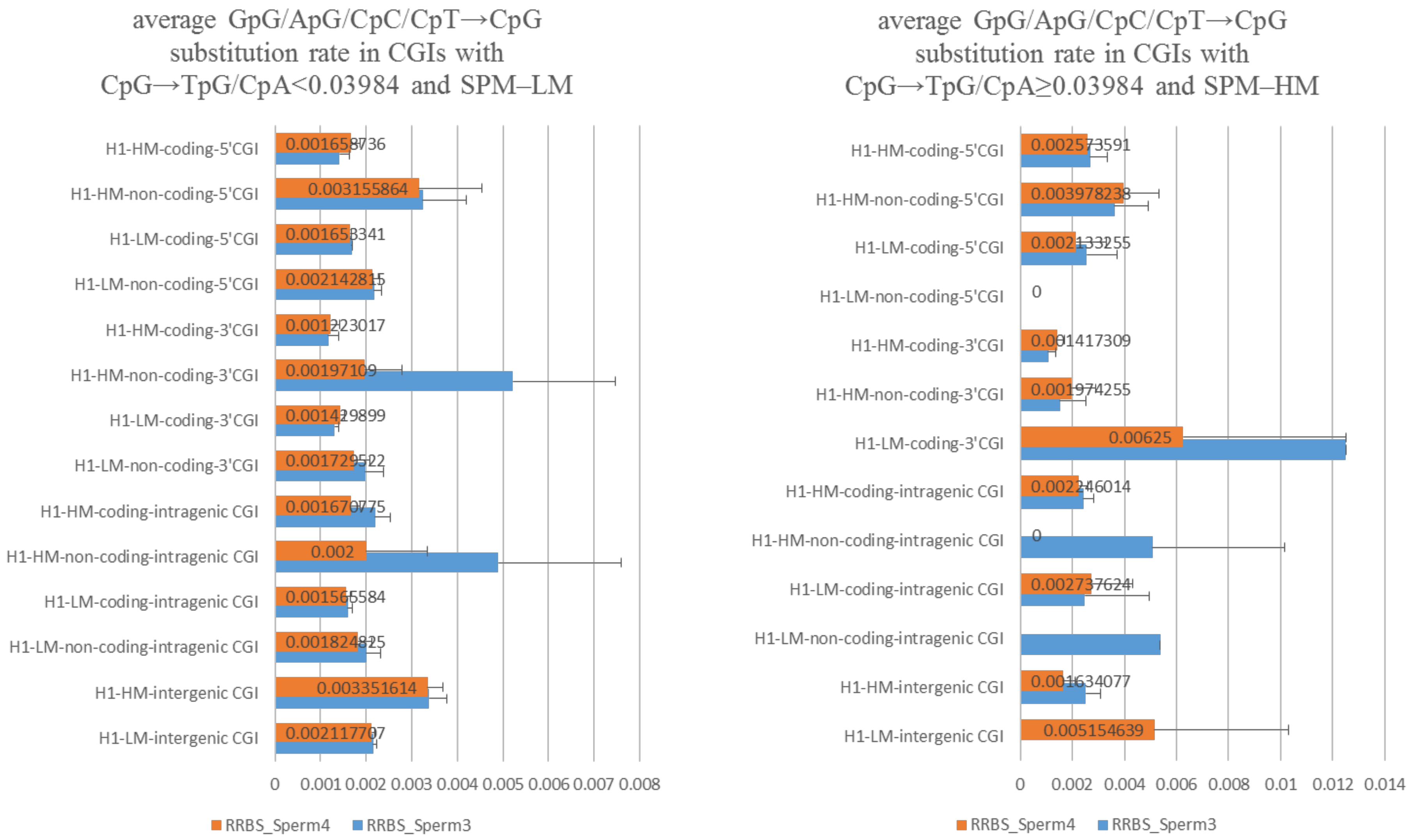

average GpG/ApG/CpC/CpT→CpG substitution rate in CGIs with CpG→TpG/CpA<0.03984 and SPM–LM
H1-HM-coding-5'CGI
H1-HM-non-coding-5'CGI
H1-LM-coding-5'CGI
H1-LM-non-coding-5'CGI
H1-HM-coding-3'CGI
H1-HM-non-coding-3'CGI
H1-LM-coding-3'CGI
H1-LM-non-coding-3'CGI
H1-HM-coding-intragenic CGI
H1-HM-non-coding-intragenic CGI
H1-LM-coding-intragenic CGI
H1-LM-non-coding-intragenic CGI
H1-HM-intergenic CGI
H1-LM-intergenic CGI
0.003155864
0.002
0.003351614
0 0.001 0.002 0.003 0.004 0.005 0.006 0.007 0.008
RRBS_Sperm4 RRBS_Sperm3
average GpG/ApG/CpC/CpT→CpG substitution rate in CGIs with CpG→TpG/CpA≥0.03984 and SPM–HM
H1-HM-coding-5'CGI
H1-HM-non-coding-5'CGI
H1-LM-coding-5'CGI
H1-LM-non-coding-5'CGI
H1-HM-coding-3'CGI
H1-HM-non-coding-3'CGI
H1-LM-coding-3'CGI
H1-HM-coding-intragenic CGI
H1-HM-non-coding-intragenic CGI
H1-LM-coding-intragenic CGI
H1-LM-non-coding-intragenic CGI
H1-HM-intergenic CGI
H1-LM-intergenic CGI
0.003978238
0
0.00625
0
0.005154639
0 0.002 0.004 0.006 0.008 0.01 0.012 0.014
RRBS_Sperm4 RRBS_Sperm3

E

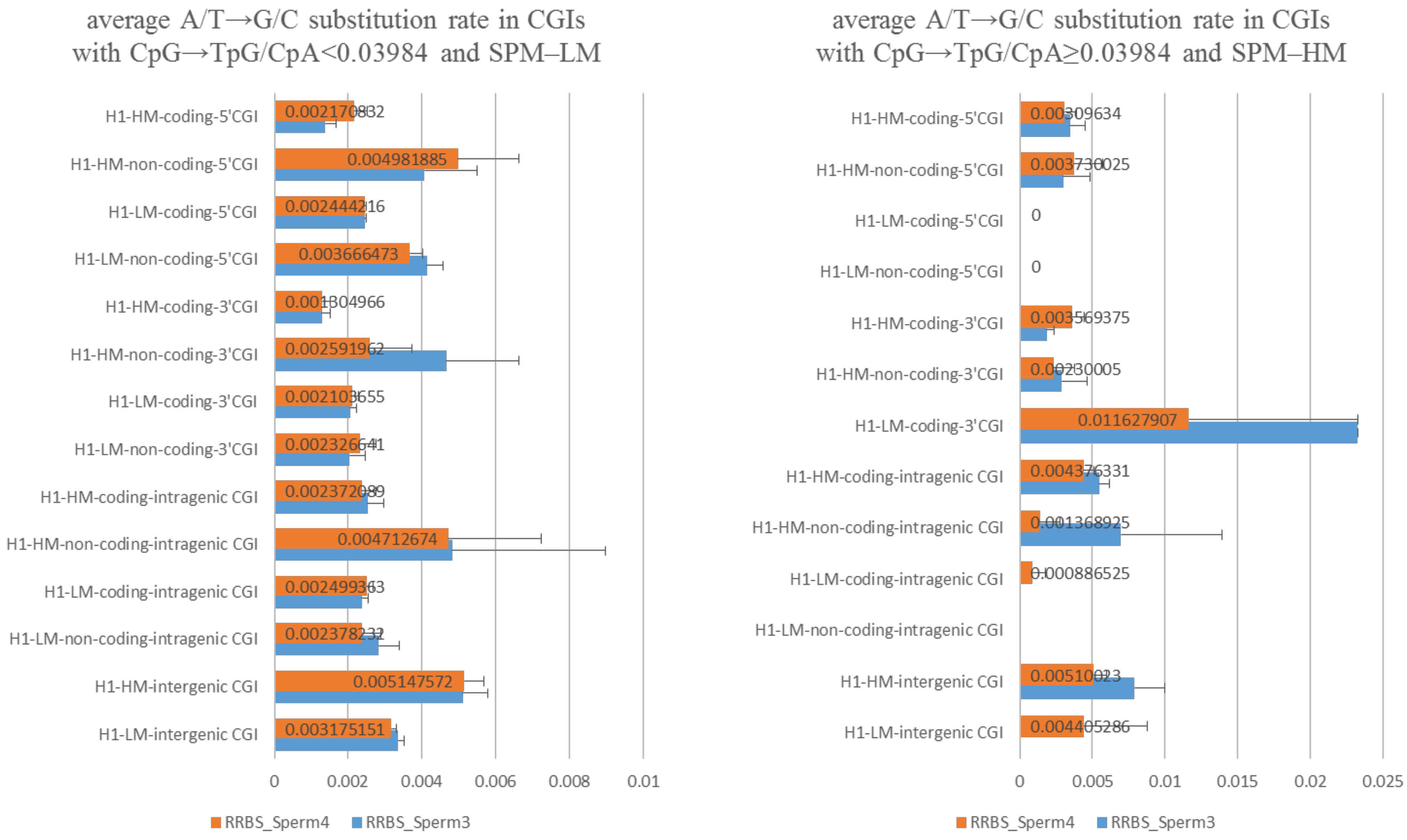
average A/T→G/C substitution rate in CGIs with CpG→TpG/CpA<0.03984 and SPM–LM
H1-HM-coding-5'CGI
H1-HM-non-coding-5'CGI
H1-LM-coding-5'CGI
H1-LM-non-coding-5'CGI
H1-HM-coding-3'CGI
H1-HM-non-coding-3'CGI
H1-LM-coding-3'CGI
H1-LM-non-coding-3'CGI
H1-HM-coding-intragenic CGI
H1-HM-non-coding-intragenic CGI
H1-LM-coding-intragenic CGI
H1-LM-non-coding-intragenic CGI
H1-HM-intergenic CGI
H1-LM-intergenic CGI
0.004981885
0.003666473
0.004712674
0.005147572
0.003175151
0
0.002
0.004
0.006
0.008
0.01
RRBS_Sperm4
RRBS_Sperm3
average A/T→G/C substitution rate in CGIs with CpG→TpG/CpA≥0.03984 and SPM–HM
H1-HM-coding-5'CGI
H1-HM-non-coding-5'CGI
H1-LM-coding-5'CGI
H1-LM-non-coding-5'CGI
H1-HM-coding-3'CGI
H1-HM-non-coding-3'CGI
H1-LM-coding-3'CGI
H1-HM-coding-intragenic CGI
H1-HM-non-coding-intragenic CGI
H1-LM-coding-intragenic CGI
H1-LM-non-coding-intragenic CGI
H1-HM-intergenic CGI
H1-LM-intergenic CGI
0.011627907
0
0.005
0.01
0.015
0.02
0.025
RRBS_Sperm4
RRBS_Sperm3

F

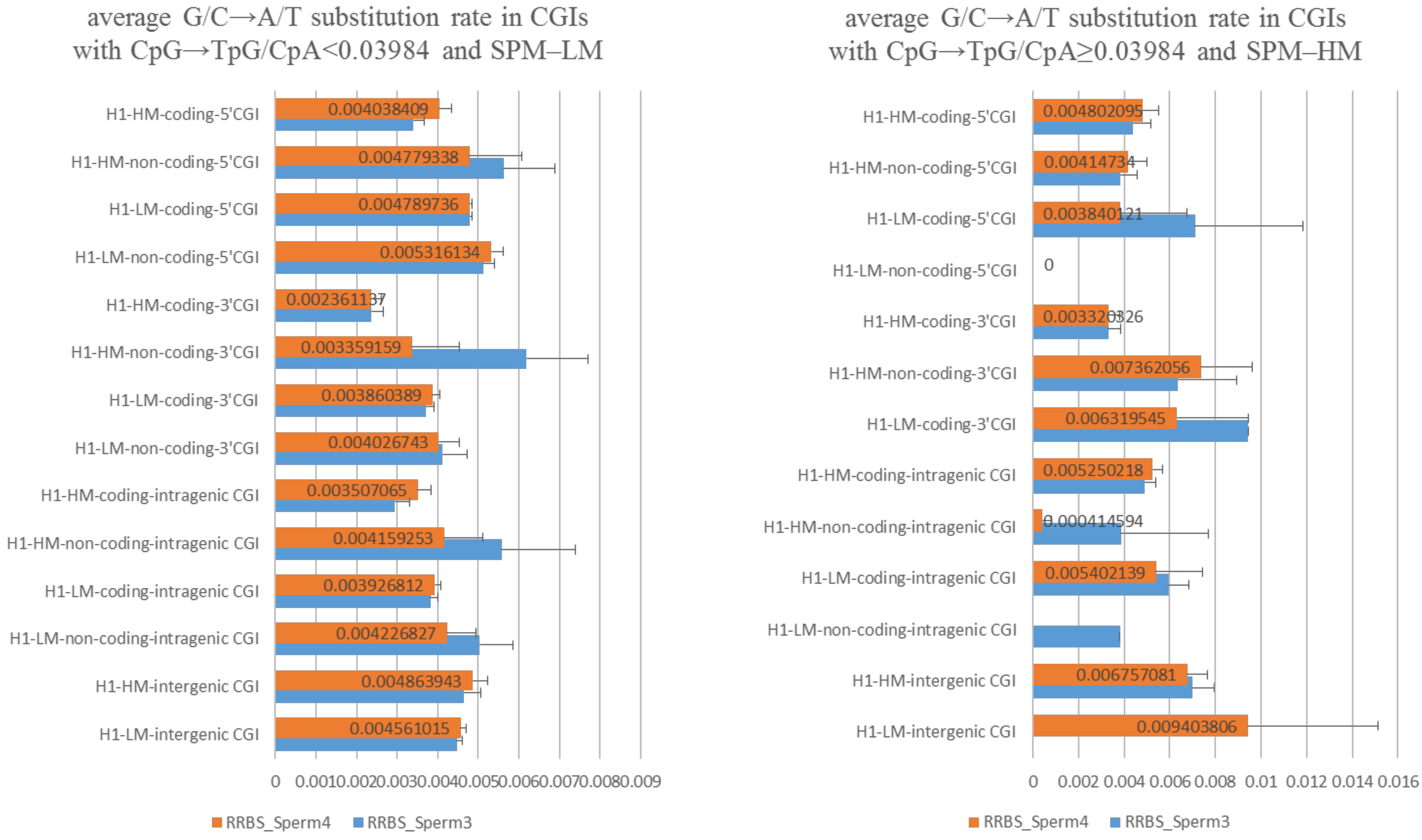

average G/C→A/T substitution rate in CGIs with CpG→TpG/CpA<0.03984 and SPM–LM
H1-HM-coding-5'CGI
H1-HM-non-coding-5'CGI
H1-LM-coding-5'CGI
H1-LM-non-coding-5'CGI
H1-HM-coding-3'CGI
H1-HM-non-coding-3'CGI
H1-LM-coding-3'CGI
H1-LM-non-coding-3'CGI
H1-HM-coding-intragenic CGI
H1-HM-non-coding-intragenic CGI
H1-LM-coding-intragenic CGI
H1-LM-non-coding-intragenic CGI
H1-HM-intergenic CGI
H1-LM-intergenic CGI
0.004038409
0.004779338
0.004789736
0.005316134
0.00236113
0.003359159
0.003860389
0.004026743
0.003507065
0.004159253
0.003926812
0.004226827
0.004863943
0.004561015
0 0.0010.0020.0030.0040.0050.0060.0070.0080.009
RRBS_Sperm4 RRBS_Sperm3
average G/C→A/T substitution rate in CGIs with CpG→TpG/CpA≥0.03984 and SPM–HM
H1-HM-coding-5'CGI
H1-HM-non-coding-5'CGI
H1-LM-coding-5'CGI
H1-LM-non-coding-5'CGI
H1-HM-coding-3'CGI
H1-HM-non-coding-3'CGI
H1-LM-coding-3'CGI
H1-HM-coding-intragenic CGI
H1-HM-non-coding-intragenic CGI
H1-LM-coding-intragenic CGI
H1-LM-non-coding-intragenic CGI
H1-HM-intergenic CGI
H1-LM-intergenic CGI
0.004802095
0.00414734
0.003840121
0
0.003320326
0.007362056
0.006319545
0.005250218
0.000414594
0.005402139
0.006757081
0.009403806
0 0.002 0.004 0.006 0.008 0.01 0.012 0.014 0.016
RRBS_Sperm4 RRBS_Sperm3
