# Supplementary material for: Factors to preserve CpG-rich sequences in methylated CpG islands
Source: BMC Genomics. 2015 Feb 28;16(1):144. doi: 10.1186/s12864-015-1286-x (PMC4417305; doi:10.1186/s12864-015-1286-x)
Supplement: Additional file 15: Figure S5. — Base substitution rate in sperm methylated CGIs with CpG → TpG/CpA < 0.03984. Hydroxymethylated CGIs in H1 cells are not included in these CGIs. The details of the figure are the same as those described in the legend to Figure 3. [file 12864_2015_1286_MOESM15_ESM.pdf]

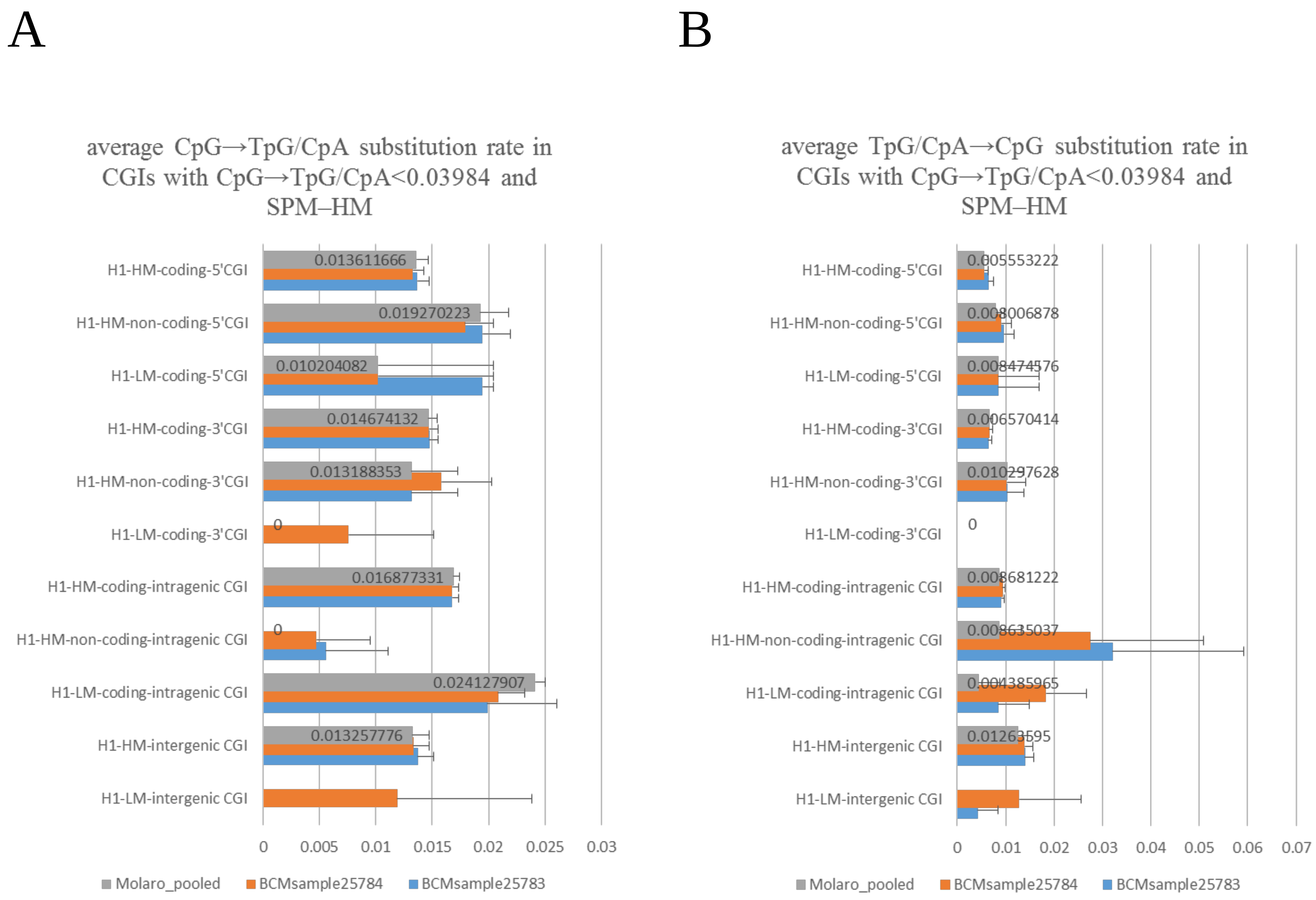

A
average CpG→TpG/CpA substitution rate in CGIs with CpG→TpG/CpA<0.03984 and SPM–HM
H1-HM-coding-5'CGI
H1-HM-non-coding-5'CGI
H1-LM-coding-5'CGI
H1-HM-coding-3'CGI
H1-HM-non-coding-3'CGI
H1-LM-coding-3'CGI
H1-HM-coding-intragenic CGI
H1-HM-non-coding-intragenic CGI
H1-LM-coding-intragenic CGI
H1-HM-intergenic CGI
H1-LM-intergenic CGI
0.013611666
0.019270223
0.010204082
0.014674132
0.013188353
0
0.016877331
0
0.024127907
0.013257776
0 0.005 0.01 0.015 0.02 0.025 0.03
Molaro_pooled BCMsample25784 BCMsample25783
B
average TpG/CpA→CpG substitution rate in CGIs with CpG→TpG/CpA<0.03984 and SPM–HM
H1-HM-coding-5'CGI
H1-HM-non-coding-5'CGI
H1-LM-coding-5'CGI
H1-HM-coding-3'CGI
H1-HM-non-coding-3'CGI
H1-LM-coding-3'CGI
H1-HM-coding-intragenic CGI
H1-HM-non-coding-intragenic CGI
H1-LM-coding-intragenic CGI
H1-HM-intergenic CGI
H1-LM-intergenic CGI
0.005553222
0.008006878
0.008474576
0.006570414
0.010297628
0
0.008681222
0.008635037
0.004385965
0.012635595
0 0.01 0.02 0.03 0.04 0.05 0.06 0.07
Molaro_pooled BCMsample25784 BCMsample25783

C

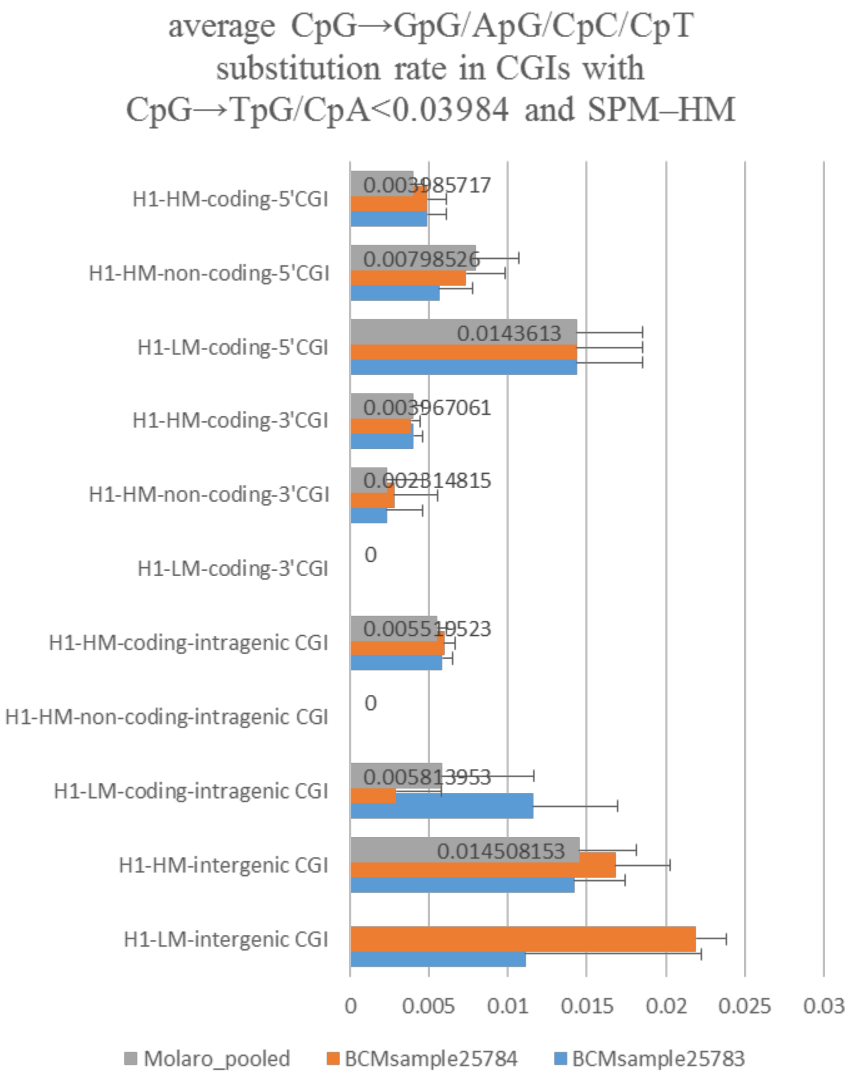
average CpG→GpG/ApG/CpC/CpT substitution rate in CGIs with CpG→TpG/CpA<0.03984 and SPM–HM
H1-HM-coding-5'CGI
H1-HM-non-coding-5'CGI
H1-LM-coding-5'CGI
H1-HM-coding-3'CGI
H1-HM-non-coding-3'CGI
H1-LM-coding-3'CGI
H1-HM-coding-intragenic CGI
H1-HM-non-coding-intragenic CGI
H1-LM-coding-intragenic CGI
H1-HM-intergenic CGI
H1-LM-intergenic CGI
0.003985717
0.00798526
0.0143613
0.003967061
0.002314815
0
0.005519523
0
0.005813953
0.014508153
0
0.005
0.01
0.015
0.02
0.025
0.03
Molaro_pooled
BCMsample25784
BCMsample25783

D

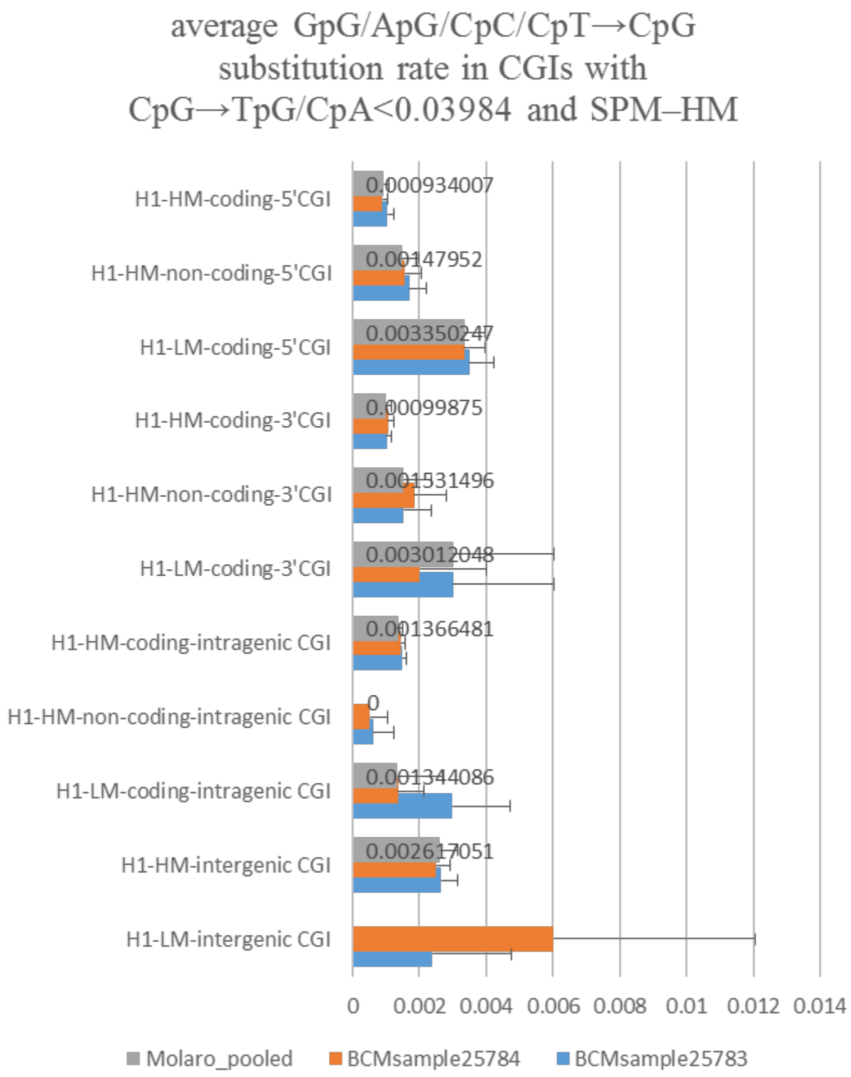
average GpG/ApG/CpC/CpT→CpG substitution rate in CGIs with CpG→TpG/CpA<0.03984 and SPM–HM
H1-HM-coding-5'CGI
H1-HM-non-coding-5'CGI
H1-LM-coding-5'CGI
H1-HM-coding-3'CGI
H1-HM-non-coding-3'CGI
H1-LM-coding-3'CGI
H1-HM-coding-intragenic CGI
H1-HM-non-coding-intragenic CGI
H1-LM-coding-intragenic CGI
H1-HM-intergenic CGI
H1-LM-intergenic CGI
0.000934007
0.00147952
0.003350247
0.00099875
0.001531496
0.003012048
0.001366481
0
0.001344086
0.002617051
0
0.002
0.004
0.006
0.008
0.01
0.012
0.014
Molaro_pooled
BCMsample25784
BCMsample25783

E

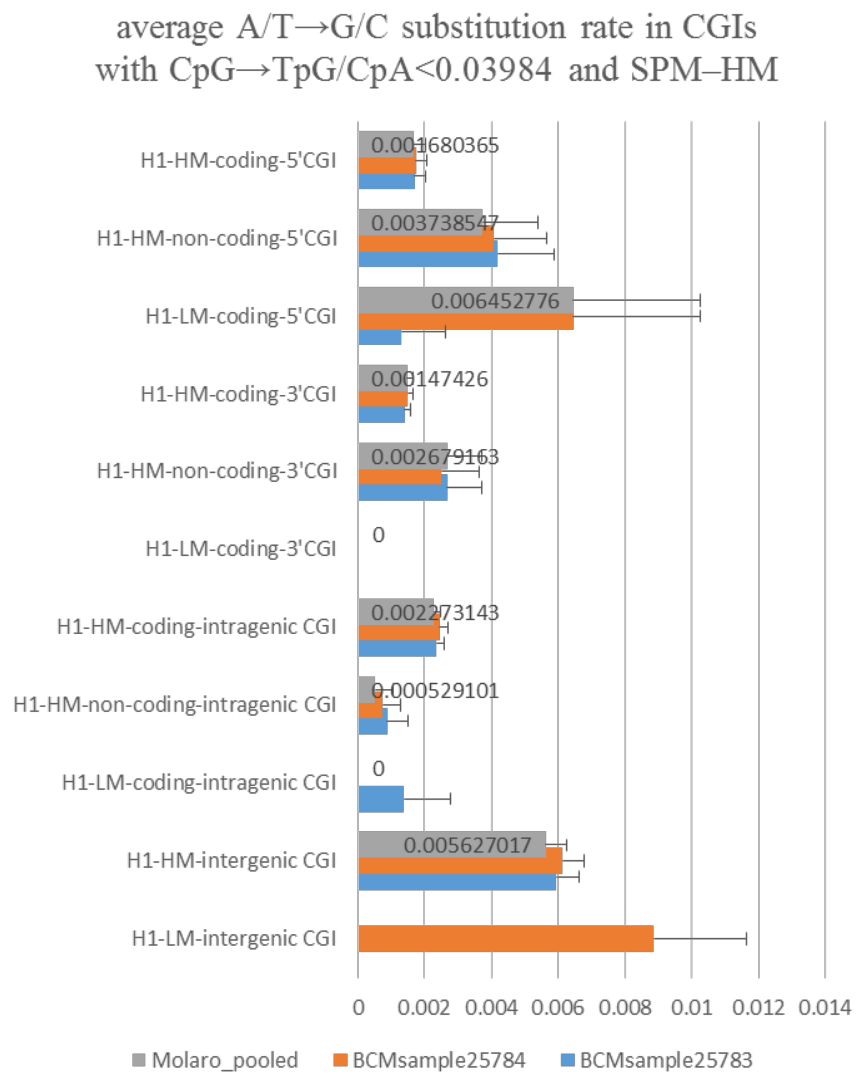
average A/T→G/C substitution rate in CGIs
with CpG→TpG/CpA<0.03984 and SPM–HM
H1-HM-coding-5'CGI
H1-HM-non-coding-5'CGI
H1-LM-coding-5'CGI
H1-HM-coding-3'CGI
H1-HM-non-coding-3'CGI
H1-LM-coding-3'CGI
H1-HM-coding-intragenic CGI
H1-HM-non-coding-intragenic CGI
H1-LM-coding-intragenic CGI
H1-HM-intergenic CGI
H1-LM-intergenic CGI
0.001680365
0.003738547
0.006452776
0.00147426
0.002679163
0
0.002273143
0.000529101
0
0.005627017
0 0.002 0.004 0.006 0.008 0.01 0.012 0.014
Molaro_pooled BCMsample25784 BCMsample25783

F

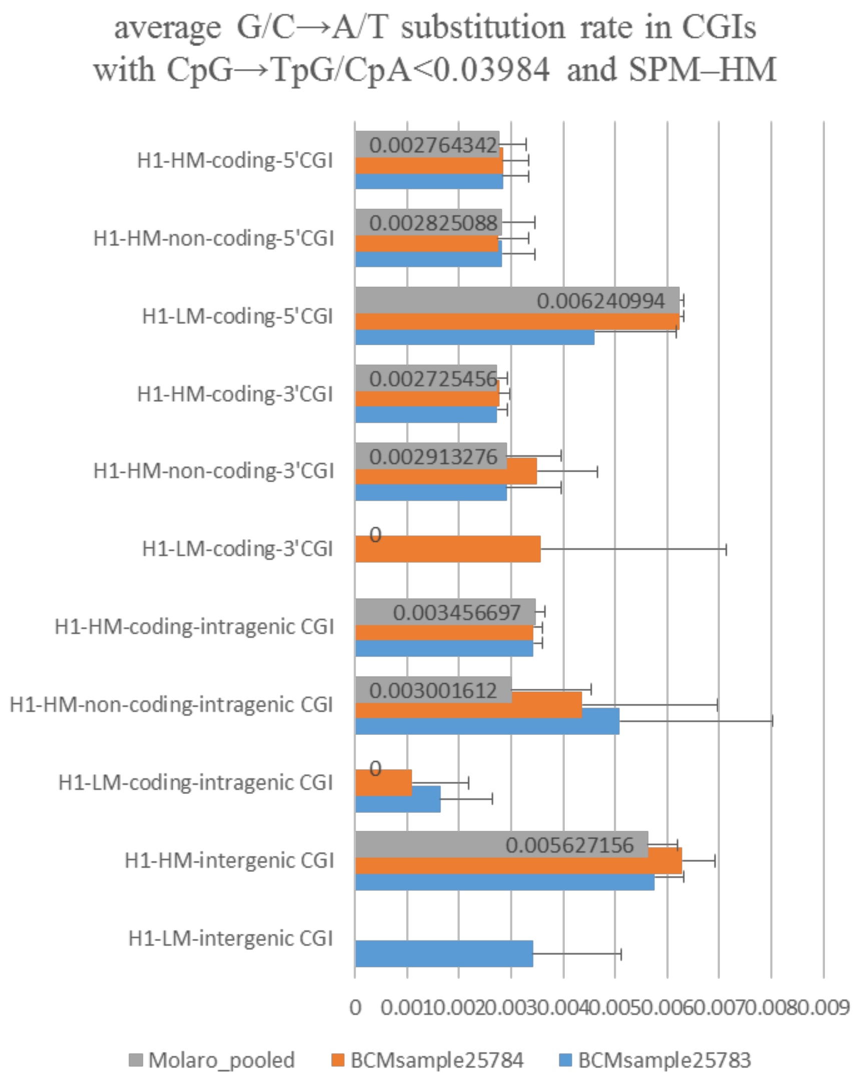
average G/C→A/T substitution rate in CGIs
with CpG→TpG/CpA<0.03984 and SPM–HM
H1-HM-coding-5'CGI
H1-HM-non-coding-5'CGI
H1-LM-coding-5'CGI
H1-HM-coding-3'CGI
H1-HM-non-coding-3'CGI
H1-LM-coding-3'CGI
H1-HM-coding-intragenic CGI
H1-HM-non-coding-intragenic CGI
H1-LM-coding-intragenic CGI
H1-HM-intergenic CGI
H1-LM-intergenic CGI
0.002764342
0.002825088
0.006240994
0.002725456
0.002913276
0
0.003456697
0.003001612
0
0.005627156
0 0.0010.0020.0030.0040.0050.0060.0070.0080.009
Molaro_pooled BCMsample25784 BCMsample25783
