# Supplementary material for: Factors to preserve CpG-rich sequences in methylated CpG islands
Source: BMC Genomics. 2015 Feb 28;16(1):144. doi: 10.1186/s12864-015-1286-x (PMC4417305; doi:10.1186/s12864-015-1286-x)
Supplement: Additional file 17: Figure S6. — Comparison of base substitution rates between lowly methylated CGIs with CpG → TpG/CpA ≥ 0.03984 and CpG → TpG/CpA < 0.03984. Hydroxymethylated CGIs in H1 cells are not included in these CGIs. The details of the figure are the same as those described in the legend to Figure 4. [file 12864_2015_1286_MOESM17_ESM.pdf]

A

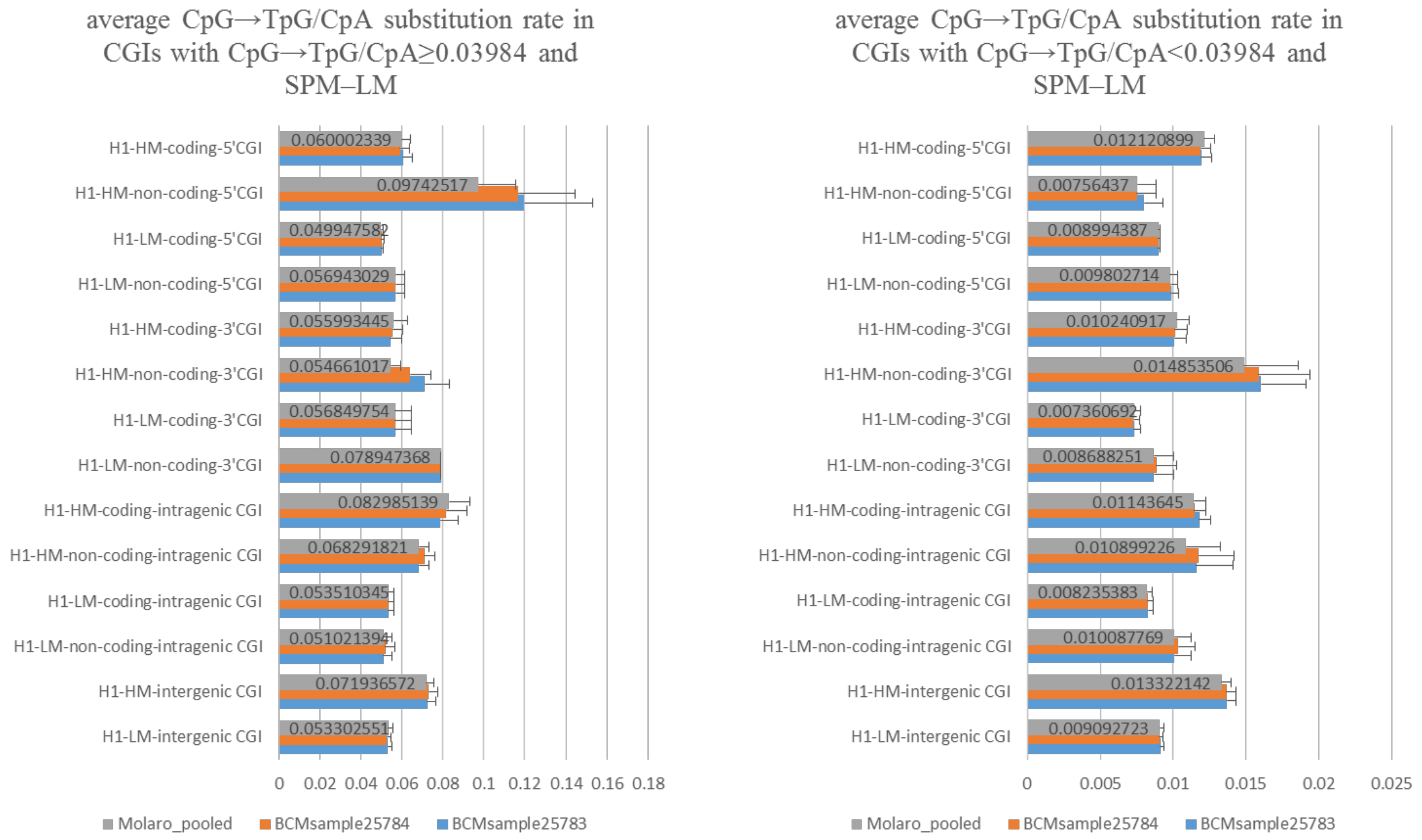
average CpG→TpG/CpA substitution rate in CGIs with CpG→TpG/CpA≥0.03984 and SPM–LM
H1-HM-coding-5'CGI
H1-HM-non-coding-5'CGI
H1-LM-coding-5'CGI
H1-LM-non-coding-5'CGI
H1-HM-coding-3'CGI
H1-HM-non-coding-3'CGI
H1-LM-coding-3'CGI
H1-LM-non-coding-3'CGI
H1-HM-coding-intragenic CGI
H1-HM-non-coding-intragenic CGI
H1-LM-coding-intragenic CGI
H1-LM-non-coding-intragenic CGI
H1-HM-intergenic CGI
H1-LM-intergenic CGI
0.060002339
0.09742517
0.049947582
0.056943029
0.055993445
0.054661017
0.056849754
0.078947368
0.082985139
0.068291821
0.053510345
0.051021394
0.071936572
0.053302551
0 0.02 0.04 0.06 0.08 0.1 0.12 0.14 0.16 0.18
Molaro_pooled
BCMsample25784
BCMsample25783
average CpG→TpG/CpA substitution rate in CGIs with CpG→TpG/CpA<0.03984 and SPM–LM
H1-HM-coding-5'CGI
H1-HM-non-coding-5'CGI
H1-LM-coding-5'CGI
H1-LM-non-coding-5'CGI
H1-HM-coding-3'CGI
H1-HM-non-coding-3'CGI
H1-LM-coding-3'CGI
H1-LM-non-coding-3'CGI
H1-HM-coding-intragenic CGI
H1-HM-non-coding-intragenic CGI
H1-LM-coding-intragenic CGI
H1-LM-non-coding-intragenic CGI
H1-HM-intergenic CGI
H1-LM-intergenic CGI
0.012120899
0.00756437
0.008994387
0.009802714
0.010240917
0.014853506
0.007360692
0.008688251
0.01143645
0.010899226
0.008235383
0.010087769
0.013322142
0.009092723
0 0.005 0.01 0.015 0.02 0.025
Molaro_pooled
BCMsample25784
BCMsample25783

B

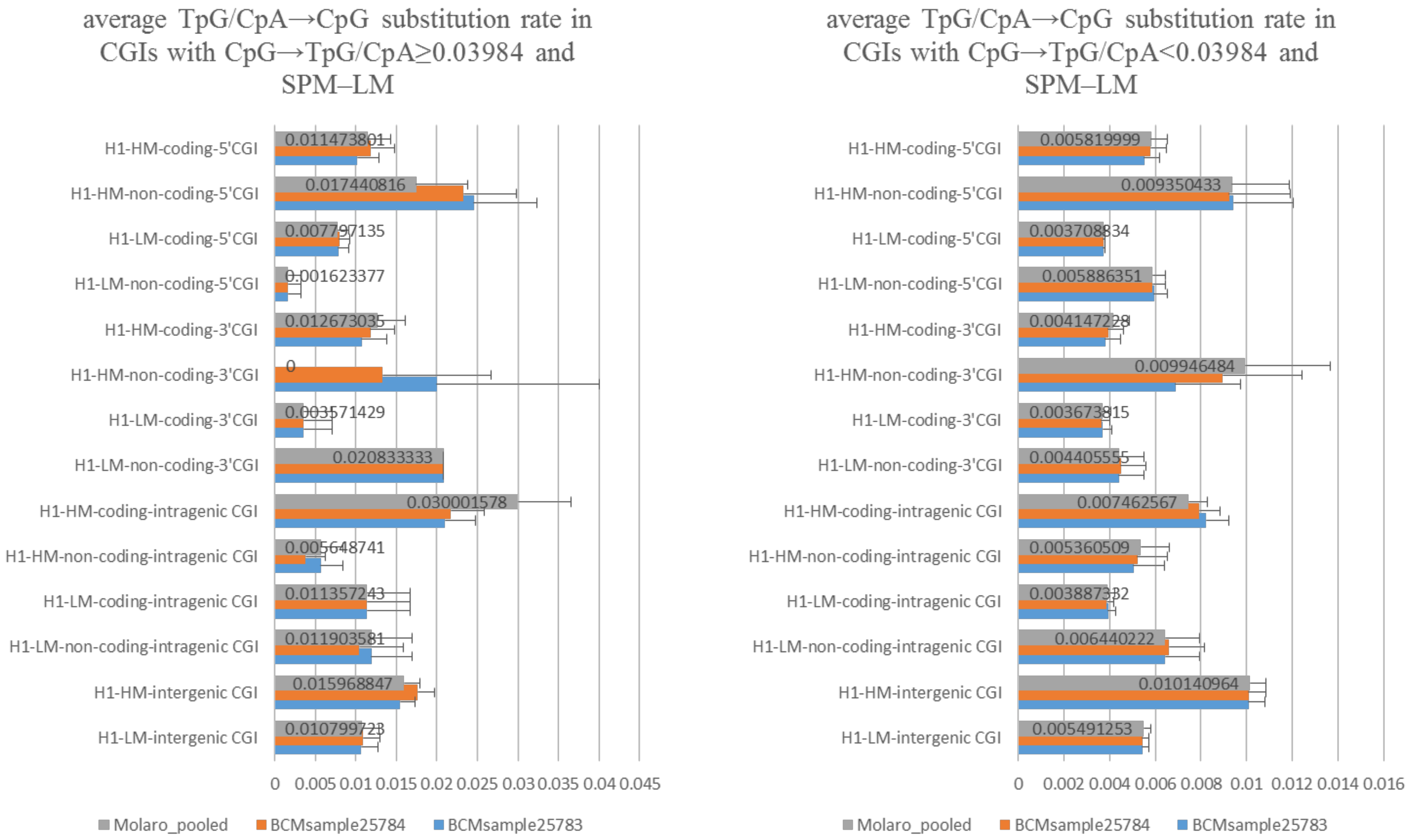
average TpG/CpA→CpG substitution rate in CGIs with CpG→TpG/CpA≥0.03984 and SPM–LM
H1-HM-coding-5'CGI
H1-HM-non-coding-5'CGI
H1-LM-coding-5'CGI
H1-LM-non-coding-5'CGI
H1-HM-coding-3'CGI
H1-HM-non-coding-3'CGI
H1-LM-coding-3'CGI
H1-LM-non-coding-3'CGI
H1-HM-coding-intragenic CGI
H1-HM-non-coding-intragenic CGI
H1-LM-coding-intragenic CGI
H1-LM-non-coding-intragenic CGI
H1-HM-intergenic CGI
H1-LM-intergenic CGI
0.011473801
0.017440816
0.007797135
0.001623377
0.012673035
0
0.003571429
0.020833333
0.030001578
0.005648741
0.011357243
0.011903581
0.015968847
0.010799723
0 0.005 0.01 0.015 0.02 0.025 0.03 0.035 0.04 0.045
Molaro_pooled
BCMsample25784
BCMsample25783
average TpG/CpA→CpG substitution rate in CGIs with CpG→TpG/CpA<0.03984 and SPM–LM
H1-HM-coding-5'CGI
H1-HM-non-coding-5'CGI
H1-LM-coding-5'CGI
H1-LM-non-coding-5'CGI
H1-HM-coding-3'CGI
H1-HM-non-coding-3'CGI
H1-LM-coding-3'CGI
H1-LM-non-coding-3'CGI
H1-HM-coding-intragenic CGI
H1-HM-non-coding-intragenic CGI
H1-LM-coding-intragenic CGI
H1-LM-non-coding-intragenic CGI
H1-HM-intergenic CGI
H1-LM-intergenic CGI
0.005819999
0.009350433
0.003708834
0.005886351
0.004147228
0.009946484
0.003673815
0.004405555
0.007462567
0.005360509
0.003887332
0.006440222
0.010140964
0.005491253
0 0.002 0.004 0.006 0.008 0.01 0.012 0.014 0.016
Molaro_pooled
BCMsample25784
BCMsample25783

C

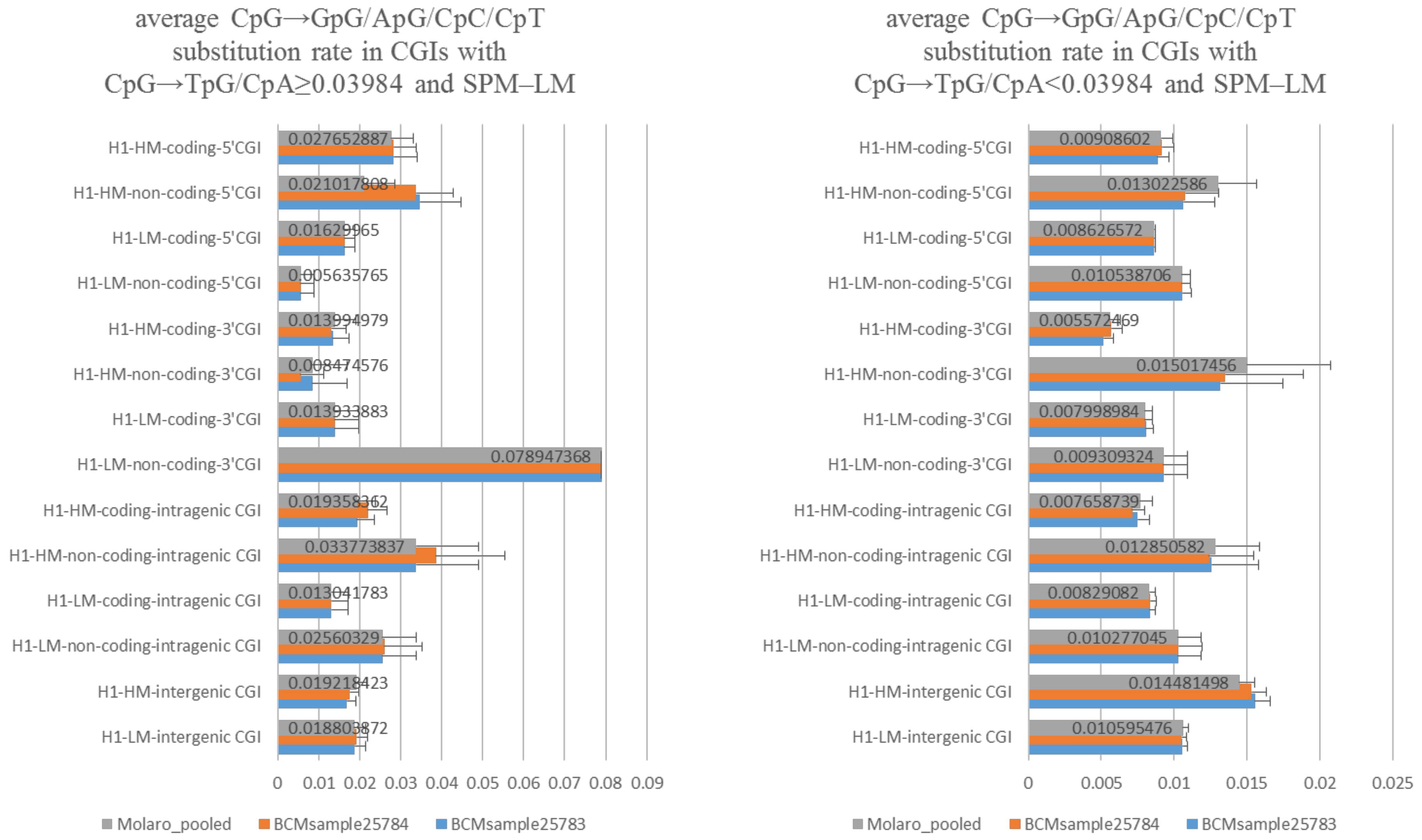

average CpG→GpG/ApG/CpC/CpT substitution rate in CGIs with CpG→TpG/CpA≥0.03984 and SPM–LM
H1-HM-coding-5'CGI
H1-HM-non-coding-5'CGI
H1-LM-coding-5'CGI
H1-LM-non-coding-5'CGI
H1-HM-coding-3'CGI
H1-HM-non-coding-3'CGI
H1-LM-coding-3'CGI
H1-LM-non-coding-3'CGI
H1-HM-coding-intragenic CGI
H1-HM-non-coding-intragenic CGI
H1-LM-coding-intragenic CGI
H1-LM-non-coding-intragenic CGI
H1-HM-intergenic CGI
H1-LM-intergenic CGI
0.027652887
0.021017808
0.01629965
0.005635765
0.013994979
0.008474576
0.013933883
0.078947368
0.019358362
0.033773837
0.013041783
0.02560329
0.019218423
0.018803872
0
0.01
0.02
0.03
0.04
0.05
0.06
0.07
0.08
0.09
Molaro_pooled
BCMsample25784
BCMsample25783
average CpG→GpG/ApG/CpC/CpT substitution rate in CGIs with CpG→TpG/CpA<0.03984 and SPM–LM
H1-HM-coding-5'CGI
H1-HM-non-coding-5'CGI
H1-LM-coding-5'CGI
H1-LM-non-coding-5'CGI
H1-HM-coding-3'CGI
H1-HM-non-coding-3'CGI
H1-LM-coding-3'CGI
H1-LM-non-coding-3'CGI
H1-HM-coding-intragenic CGI
H1-HM-non-coding-intragenic CGI
H1-LM-coding-intragenic CGI
H1-LM-non-coding-intragenic CGI
H1-HM-intergenic CGI
H1-LM-intergenic CGI
0.00908602
0.013022586
0.008626572
0.010538706
0.005572469
0.015017456
0.007998984
0.009309324
0.007658739
0.012850582
0.00829082
0.010277045
0.014481498
0.010595476
0
0.005
0.01
0.015
0.02
0.025
Molaro_pooled
BCMsample25784
BCMsample25783

D

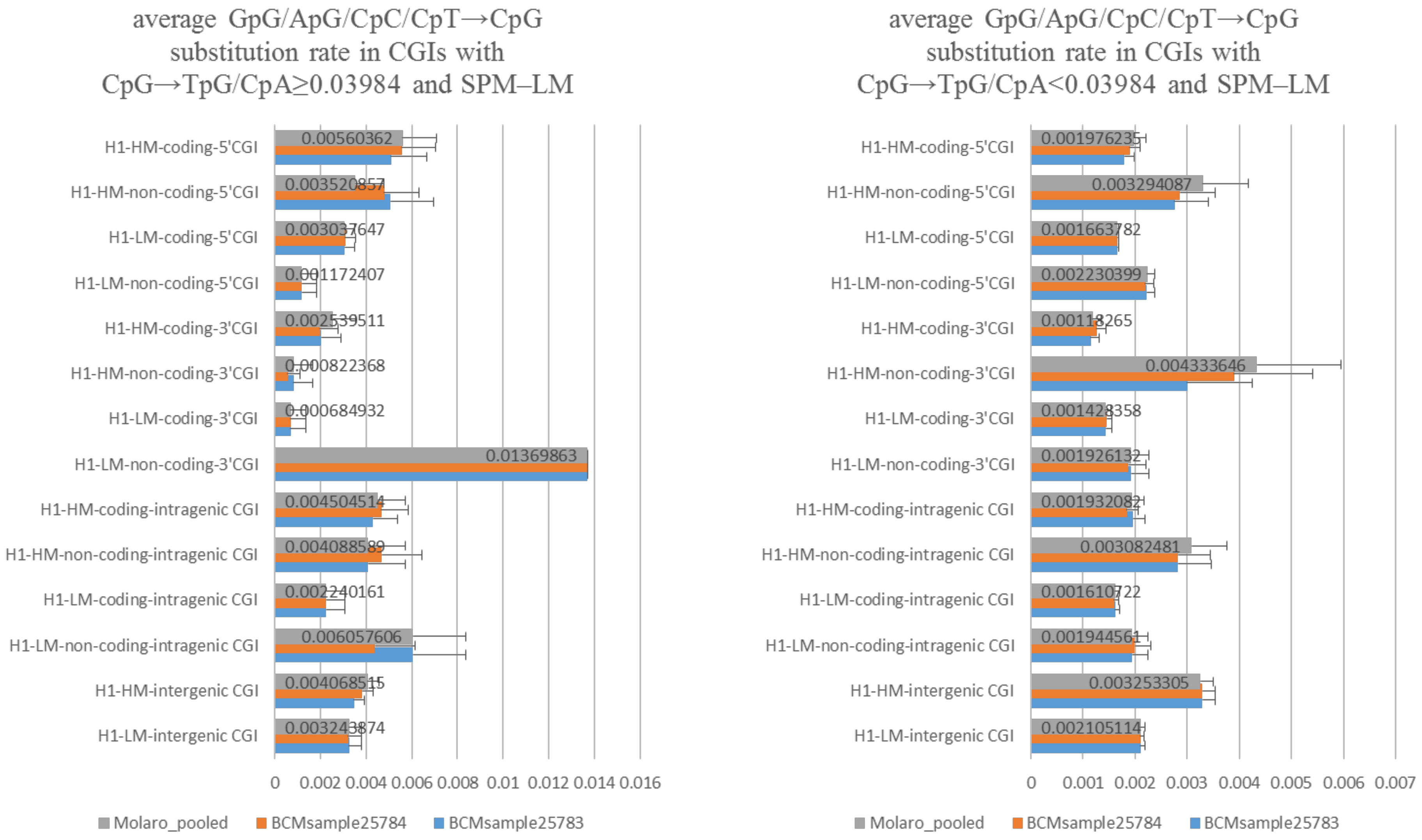
average GpG/ApG/CpC/CpT→CpG substitution rate in CGIs with CpG→TpG/CpA≥0.03984 and SPM–LM
H1-HM-coding-5'CGI
H1-HM-non-coding-5'CGI
H1-LM-coding-5'CGI
H1-LM-non-coding-5'CGI
H1-HM-coding-3'CGI
H1-HM-non-coding-3'CGI
H1-LM-coding-3'CGI
H1-LM-non-coding-3'CGI
H1-HM-coding-intragenic CGI
H1-HM-non-coding-intragenic CGI
H1-LM-coding-intragenic CGI
H1-LM-non-coding-intragenic CGI
H1-HM-intergenic CGI
H1-LM-intergenic CGI
0.00560362
0.003520857
0.003037647
0.001172407
0.002539511
0.000822368
0.000684932
0.01369863
0.004504514
0.004088589
0.002240161
0.006057606
0.004068515
0.003243874
0 0.002 0.004 0.006 0.008 0.01 0.012 0.014 0.016
Molaro_pooled BCMsample25784 BCMsample25783
average GpG/ApG/CpC/CpT→CpG substitution rate in CGIs with CpG→TpG/CpA<0.03984 and SPM–LM
H1-HM-coding-5'CGI
H1-HM-non-coding-5'CGI
H1-LM-coding-5'CGI
H1-LM-non-coding-5'CGI
H1-HM-coding-3'CGI
H1-HM-non-coding-3'CGI
H1-LM-coding-3'CGI
H1-LM-non-coding-3'CGI
H1-HM-coding-intragenic CGI
H1-HM-non-coding-intragenic CGI
H1-LM-coding-intragenic CGI
H1-LM-non-coding-intragenic CGI
H1-HM-intergenic CGI
H1-LM-intergenic CGI
0.001976235
0.003294087
0.001663782
0.002230399
0.001138265
0.004333646
0.001428358
0.001926132
0.001932082
0.003082481
0.001610722
0.001944561
0.003253305
0.002105114
0 0.001 0.002 0.003 0.004 0.005 0.006 0.007
Molaro_pooled BCMsample25784 BCMsample25783

E

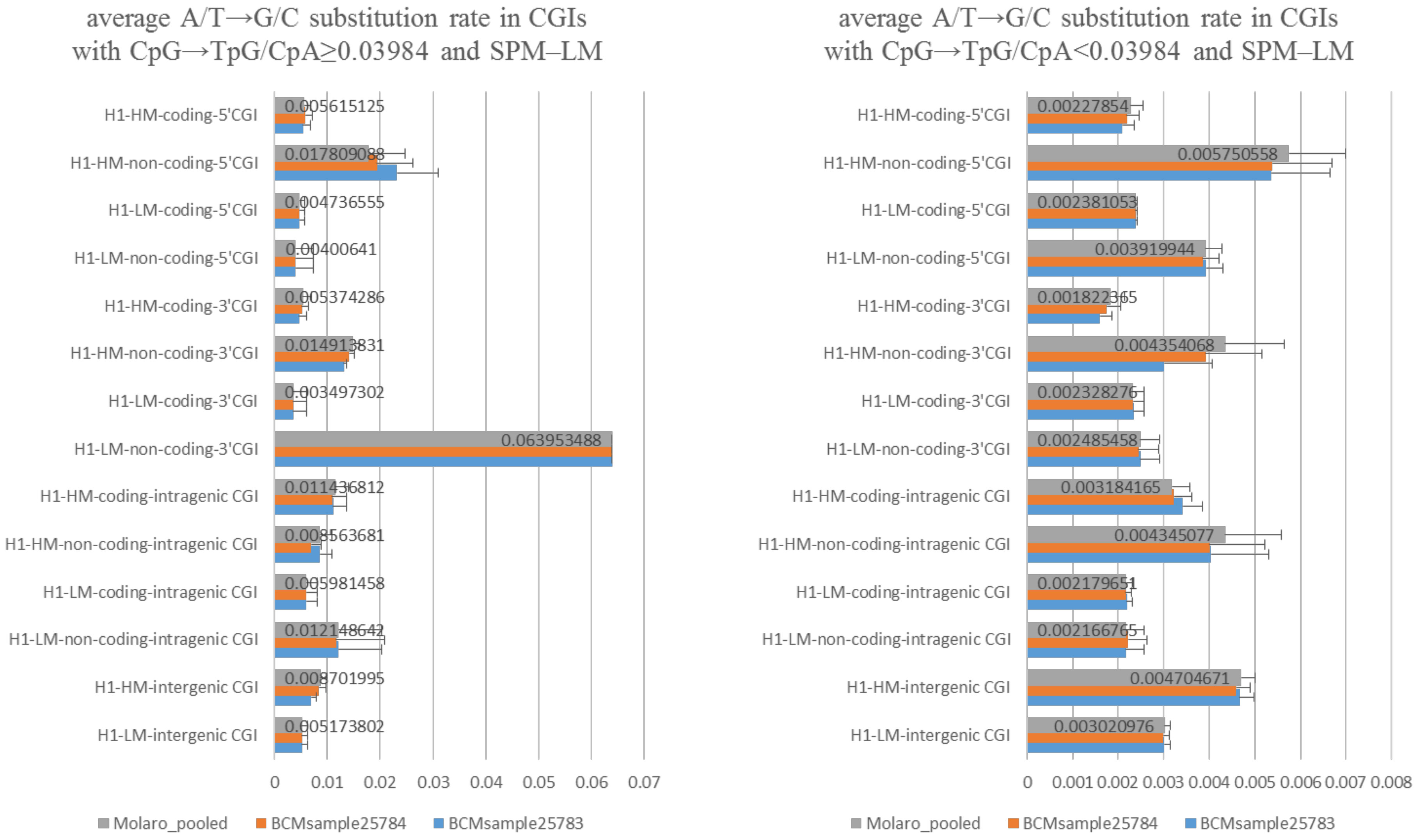

average A/T→G/C substitution rate in CGIs with CpG→TpG/CpA≥0.03984 and SPM–LM
H1-HM-coding-5'CGI
H1-HM-non-coding-5'CGI
H1-LM-coding-5'CGI
H1-LM-non-coding-5'CGI
H1-HM-coding-3'CGI
H1-HM-non-coding-3'CGI
H1-LM-coding-3'CGI
H1-LM-non-coding-3'CGI
H1-HM-coding-intragenic CGI
H1-HM-non-coding-intragenic CGI
H1-LM-coding-intragenic CGI
H1-LM-non-coding-intragenic CGI
H1-HM-intergenic CGI
H1-LM-intergenic CGI
0.005615125
0.017809088
0.004736555
0.00400641
0.005374286
0.014913831
0.003497302
0.063953488
0.011436812
0.008563681
0.005981458
0.012148642
0.008701995
0.005173802
0
0.01
0.02
0.03
0.04
0.05
0.06
0.07
Molaro_pooled
BCMsample25784
BCMsample25783
average A/T→G/C substitution rate in CGIs with CpG→TpG/CpA<0.03984 and SPM–LM
H1-HM-coding-5'CGI
H1-HM-non-coding-5'CGI
H1-LM-coding-5'CGI
H1-LM-non-coding-5'CGI
H1-HM-coding-3'CGI
H1-HM-non-coding-3'CGI
H1-LM-coding-3'CGI
H1-LM-non-coding-3'CGI
H1-HM-coding-intragenic CGI
H1-HM-non-coding-intragenic CGI
H1-LM-coding-intragenic CGI
H1-LM-non-coding-intragenic CGI
H1-HM-intergenic CGI
H1-LM-intergenic CGI
0.00227854
0.005750558
0.002381053
0.003919944
0.001822365
0.004354068
0.002328276
0.002485458
0.003184165
0.004345077
0.002179651
0.002166765
0.004704671
0.003020976
0
0.001
0.002
0.003
0.004
0.005
0.006
0.007
0.008
Molaro_pooled
BCMsample25784
BCMsample25783

F

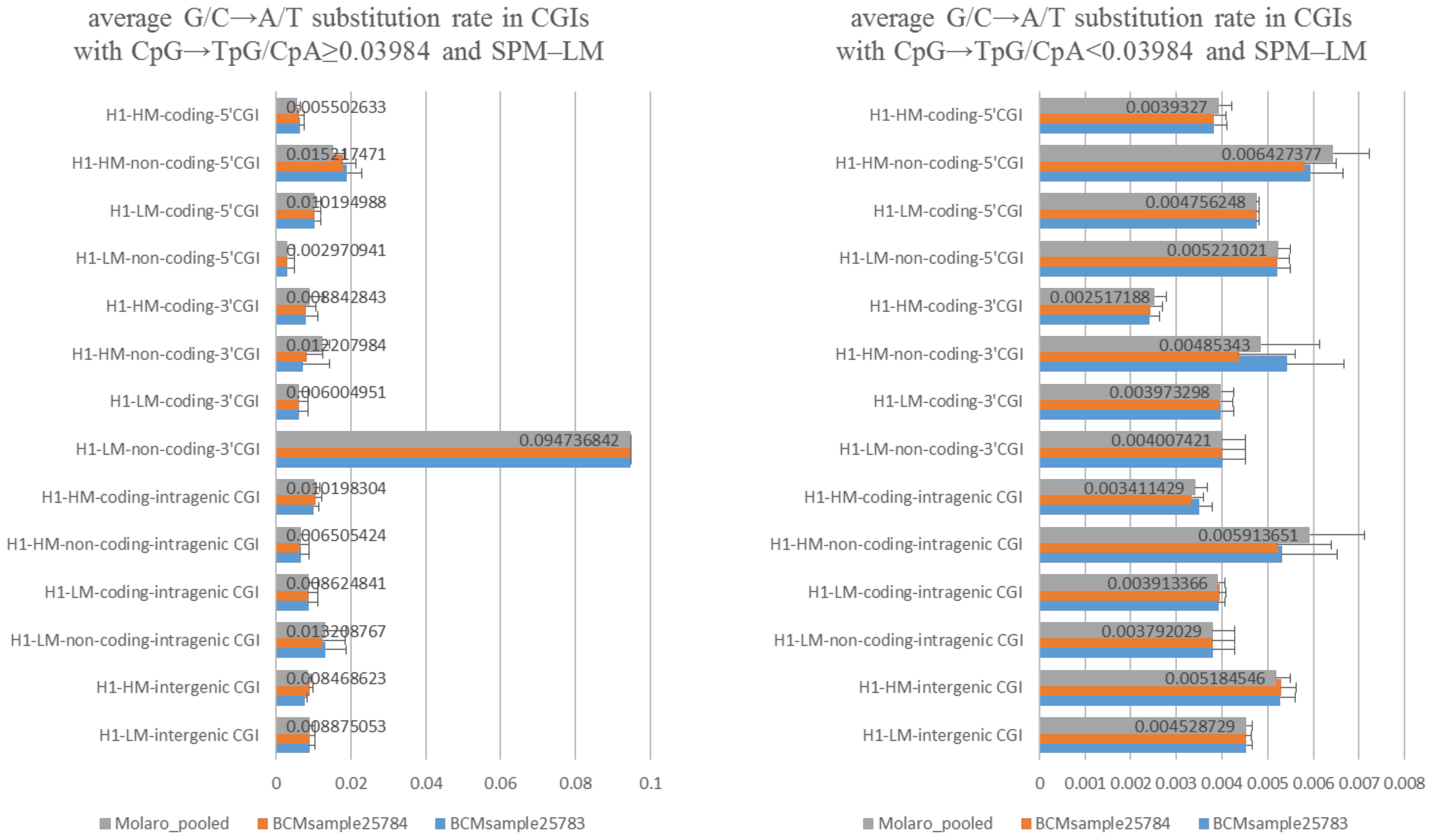
average G/C→A/T substitution rate in CGIs
with CpG→TpG/CpA≥0.03984 and SPM–LM
H1-HM-coding-5'CGI
H1-HM-non-coding-5'CGI
H1-LM-coding-5'CGI
H1-LM-non-coding-5'CGI
H1-HM-coding-3'CGI
H1-HM-non-coding-3'CGI
H1-LM-coding-3'CGI
H1-LM-non-coding-3'CGI
H1-HM-coding-intragenic CGI
H1-HM-non-coding-intragenic CGI
H1-LM-coding-intragenic CGI
H1-LM-non-coding-intragenic CGI
H1-HM-intergenic CGI
H1-LM-intergenic CGI
0.005502633
0.015217471
0.010194988
0.002970941
0.008842843
0.012207984
0.006004951
0.094736842
0.010198304
0.006505424
0.008624841
0.013208767
0.008468623
0.008875053
0
0.02
0.04
0.06
0.08
0.1
Molaro_pooled
BCMsample25784
BCMsample25783
average G/C→A/T substitution rate in CGIs
with CpG→TpG/CpA<0.03984 and SPM–LM
H1-HM-coding-5'CGI
H1-HM-non-coding-5'CGI
H1-LM-coding-5'CGI
H1-LM-non-coding-5'CGI
H1-HM-coding-3'CGI
H1-HM-non-coding-3'CGI
H1-LM-coding-3'CGI
H1-LM-non-coding-3'CGI
H1-HM-coding-intragenic CGI
H1-HM-non-coding-intragenic CGI
H1-LM-coding-intragenic CGI
H1-LM-non-coding-intragenic CGI
H1-HM-intergenic CGI
H1-LM-intergenic CGI
0.0039327
0.006427377
0.004756248
0.005221021
0.002517188
0.00485343
0.003973298
0.004007421
0.003411429
0.005913651
0.003913366
0.003792029
0.005184546
0.004528729
0
0.001
0.002
0.003
0.004
0.005
0.006
0.007
0.008
Molaro_pooled
BCMsample25784
BCMsample25783
