# Supplementary material for: Factors to preserve CpG-rich sequences in methylated CpG islands
Source: BMC Genomics. 2015 Feb 28;16(1):144. doi: 10.1186/s12864-015-1286-x (PMC4417305; doi:10.1186/s12864-015-1286-x)
Supplement: Additional file 18: Figure S7. — Comparison of base substitution rates between CGIs with SPM-LM and SPM-HM. Hydroxymethylated CGIs in H1 cells are not included in these CGIs. The details of the figure are the same as those described in the legend to Figure 5. [file 12864_2015_1286_MOESM18_ESM.pdf]

A

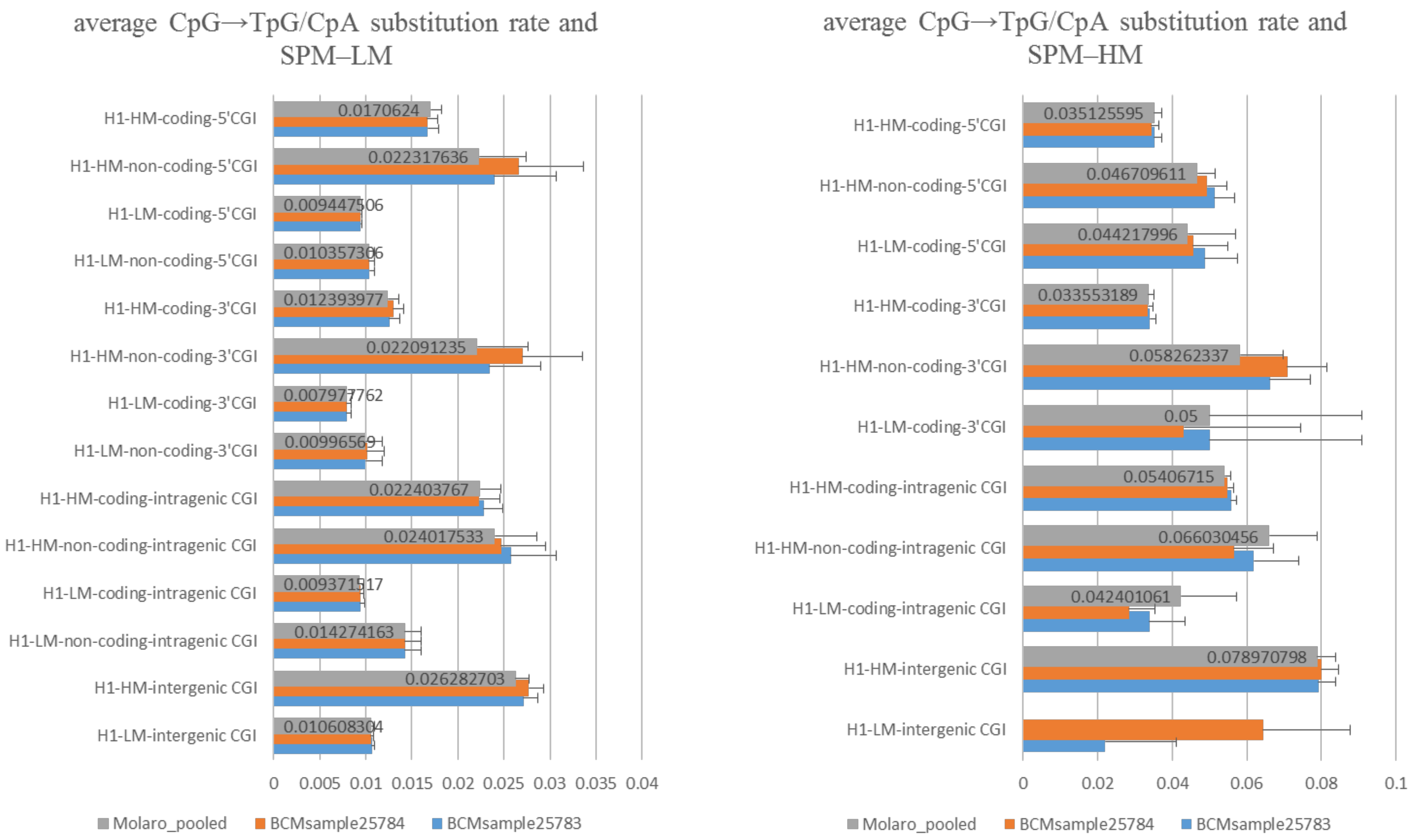

average CpG→TpG/CpA substitution rate and SPM–LM
H1-HM-coding-5'CGI
H1-HM-non-coding-5'CGI
H1-LM-coding-5'CGI
H1-LM-non-coding-5'CGI
H1-HM-coding-3'CGI
H1-HM-non-coding-3'CGI
H1-LM-coding-3'CGI
H1-LM-non-coding-3'CGI
H1-HM-coding-intragenic CGI
H1-HM-non-coding-intragenic CGI
H1-LM-coding-intragenic CGI
H1-LM-non-coding-intragenic CGI
H1-HM-intergenic CGI
H1-LM-intergenic CGI
0.0170624
0.022317636
0.009447506
0.010357306
0.012393977
0.022091235
0.007977762
0.00996569
0.022403767
0.024017533
0.009371517
0.014274163
0.026282703
0.010608304
0
0.005
0.01
0.015
0.02
0.025
0.03
0.035
0.04
Molaro_pooled
BCMsample25784
BCMsample25783
average CpG→TpG/CpA substitution rate and SPM–HM
H1-HM-coding-5'CGI
H1-HM-non-coding-5'CGI
H1-LM-coding-5'CGI
H1-HM-coding-3'CGI
H1-HM-non-coding-3'CGI
H1-LM-coding-3'CGI
H1-HM-coding-intragenic CGI
H1-HM-non-coding-intragenic CGI
H1-LM-coding-intragenic CGI
H1-HM-intergenic CGI
H1-LM-intergenic CGI
0.035125595
0.046709611
0.044217996
0.033553189
0.058262337
0.05
0.05406715
0.066030456
0.042401061
0.078970798
0
0.02
0.04
0.06
0.08
0.1
Molaro_pooled
BCMsample25784
BCMsample25783

B

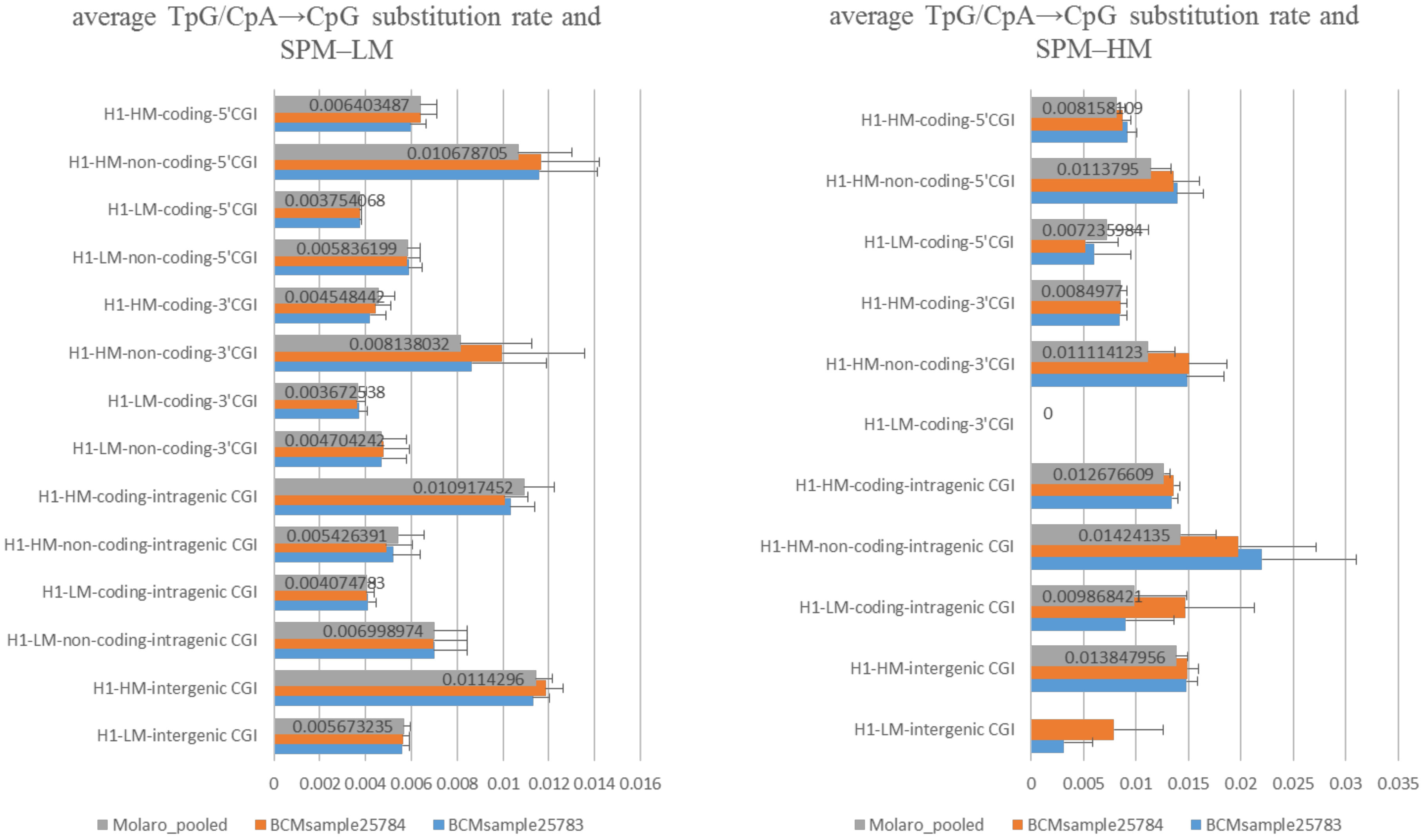
average TpG/CpA→CpG substitution rate and SPM–LM
H1-HM-coding-5'CGI
H1-HM-non-coding-5'CGI
H1-LM-coding-5'CGI
H1-LM-non-coding-5'CGI
H1-HM-coding-3'CGI
H1-HM-non-coding-3'CGI
H1-LM-coding-3'CGI
H1-LM-non-coding-3'CGI
H1-HM-coding-intragenic CGI
H1-HM-non-coding-intragenic CGI
H1-LM-coding-intragenic CGI
H1-LM-non-coding-intragenic CGI
H1-HM-intergenic CGI
H1-LM-intergenic CGI
0.006403487
0.010678705
0.003754068
0.005836199
0.004548442
0.008138032
0.003672538
0.004704242
0.010917452
0.005426391
0.004074783
0.006998974
0.0114296
0.005673235
0
0.002
0.004
0.006
0.008
0.01
0.012
0.014
0.016
Molaro_pooled
BCMsample25784
BCMsample25783
average TpG/CpA→CpG substitution rate and SPM–HM
H1-HM-coding-5'CGI
H1-HM-non-coding-5'CGI
H1-LM-coding-5'CGI
H1-HM-coding-3'CGI
H1-HM-non-coding-3'CGI
H1-LM-coding-3'CGI
H1-HM-coding-intragenic CGI
H1-HM-non-coding-intragenic CGI
H1-LM-coding-intragenic CGI
H1-HM-intergenic CGI
H1-LM-intergenic CGI
0.008158109
0.0113795
0.007235984
0.0084977
0.011114123
0
0.012676609
0.01424135
0.009868421
0.013847956
0
0.005
0.01
0.015
0.02
0.025
0.03
0.035
Molaro_pooled
BCMsample25784
BCMsample25783

C

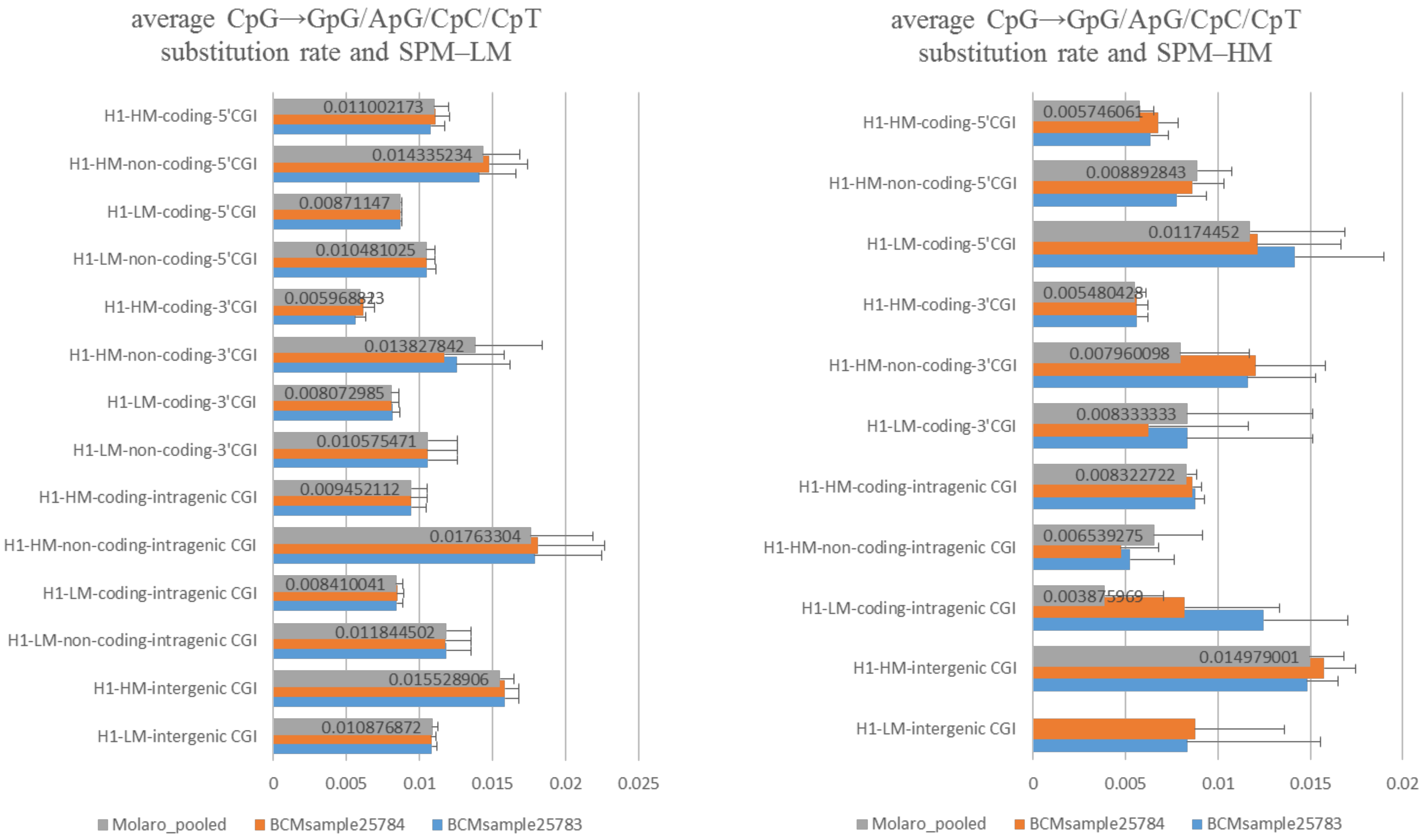
average CpG→GpG/ApG/CpC/CpT substitution rate and SPM–LM
H1-HM-coding-5'CGI
H1-HM-non-coding-5'CGI
H1-LM-coding-5'CGI
H1-LM-non-coding-5'CGI
H1-HM-coding-3'CGI
H1-HM-non-coding-3'CGI
H1-LM-coding-3'CGI
H1-LM-non-coding-3'CGI
H1-HM-coding-intragenic CGI
H1-HM-non-coding-intragenic CGI
H1-LM-coding-intragenic CGI
H1-LM-non-coding-intragenic CGI
H1-HM-intergenic CGI
H1-LM-intergenic CGI
0.011002173
0.014335234
0.00871147
0.010481025
0.005968823
0.013827842
0.008072985
0.010575471
0.009452112
0.01763304
0.008410041
0.011844502
0.015528906
0.010876872
0
0.005
0.01
0.015
0.02
0.025
Molaro_pooled
BCMsample25784
BCMsample25783
average CpG→GpG/ApG/CpC/CpT substitution rate and SPM–HM
H1-HM-coding-5'CGI
H1-HM-non-coding-5'CGI
H1-LM-coding-5'CGI
H1-HM-coding-3'CGI
H1-HM-non-coding-3'CGI
H1-LM-coding-3'CGI
H1-HM-coding-intragenic CGI
H1-HM-non-coding-intragenic CGI
H1-LM-coding-intragenic CGI
H1-HM-intergenic CGI
H1-LM-intergenic CGI
0.005746061
0.008892843
0.01174452
0.005480428
0.007960098
0.008333333
0.008322722
0.006539275
0.003875969
0.014979001
0
0.005
0.01
0.015
0.02
Molaro_pooled
BCMsample25784
BCMsample25783

D

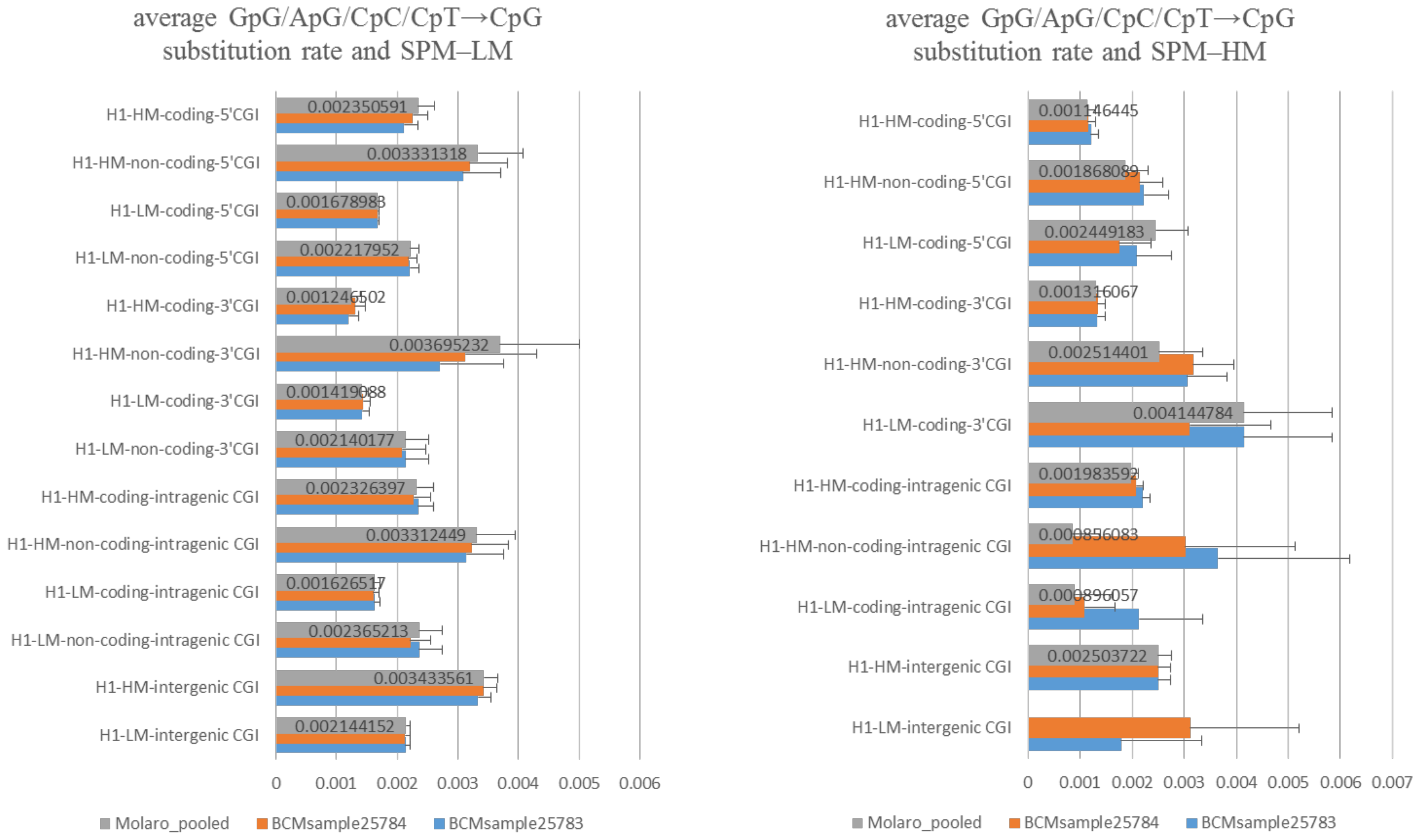

average GpG/ApG/CpC/CpT→CpG substitution rate and SPM–LM
H1-HM-coding-5'CGI
H1-HM-non-coding-5'CGI
H1-LM-coding-5'CGI
H1-LM-non-coding-5'CGI
H1-HM-coding-3'CGI
H1-HM-non-coding-3'CGI
H1-LM-coding-3'CGI
H1-LM-non-coding-3'CGI
H1-HM-coding-intragenic CGI
H1-HM-non-coding-intragenic CGI
H1-LM-coding-intragenic CGI
H1-LM-non-coding-intragenic CGI
H1-HM-intergenic CGI
H1-LM-intergenic CGI
0.002350591
0.003331318
0.001678983
0.002217952
0.001246502
0.003695232
0.001419088
0.002140177
0.002326397
0.003312449
0.001626517
0.002365213
0.003433561
0.002144152
0
0.001
0.002
0.003
0.004
0.005
0.006
Molaro_pooled
BCMsample25784
BCMsample25783
average GpG/ApG/CpC/CpT→CpG substitution rate and SPM–HM
H1-HM-coding-5'CGI
H1-HM-non-coding-5'CGI
H1-LM-coding-5'CGI
H1-HM-coding-3'CGI
H1-HM-non-coding-3'CGI
H1-LM-coding-3'CGI
H1-HM-coding-intragenic CGI
H1-HM-non-coding-intragenic CGI
H1-LM-coding-intragenic CGI
H1-HM-intergenic CGI
H1-LM-intergenic CGI
0.001146445
0.001868089
0.002449183
0.001316067
0.002514401
0.004144784
0.001983592
0.000856083
0.000896057
0.002503722
0
0.001
0.002
0.003
0.004
0.005
0.006
0.007
Molaro_pooled
BCMsample25784
BCMsample25783

E

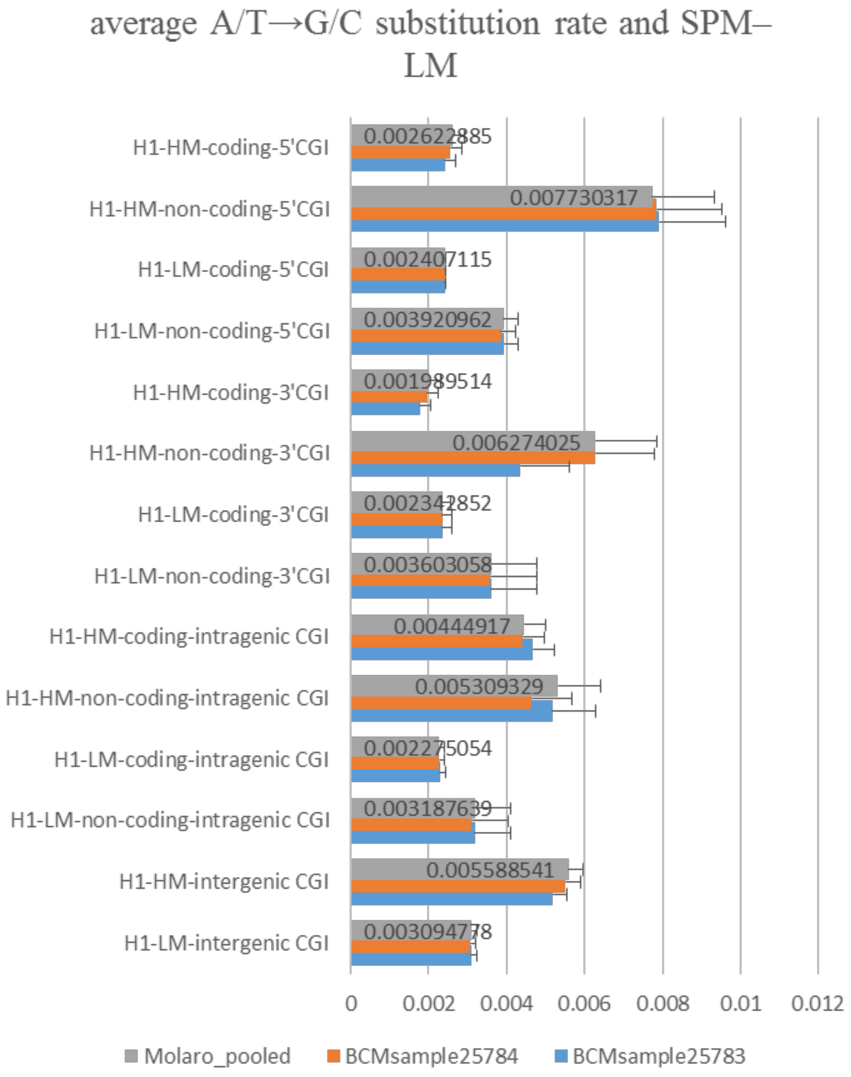

average A/T→G/C substitution rate and SPM–LM
H1-HM-coding-5'CGI
H1-HM-non-coding-5'CGI
H1-LM-coding-5'CGI
H1-LM-non-coding-5'CGI
H1-HM-coding-3'CGI
H1-HM-non-coding-3'CGI
H1-LM-coding-3'CGI
H1-LM-non-coding-3'CGI
H1-HM-coding-intragenic CGI
H1-HM-non-coding-intragenic CGI
H1-LM-coding-intragenic CGI
H1-LM-non-coding-intragenic CGI
H1-HM-intergenic CGI
H1-LM-intergenic CGI
0.002622885
0.007730317
0.002407115
0.003920962
0.001989514
0.006274025
0.002342852
0.003603058
0.00444917
0.005309329
0.002275054
0.003187639
0.005588541
0.003094778
0
0.002
0.004
0.006
0.008
0.01
0.012
Molaro_pooled
BCMsample25784
BCMsample25783

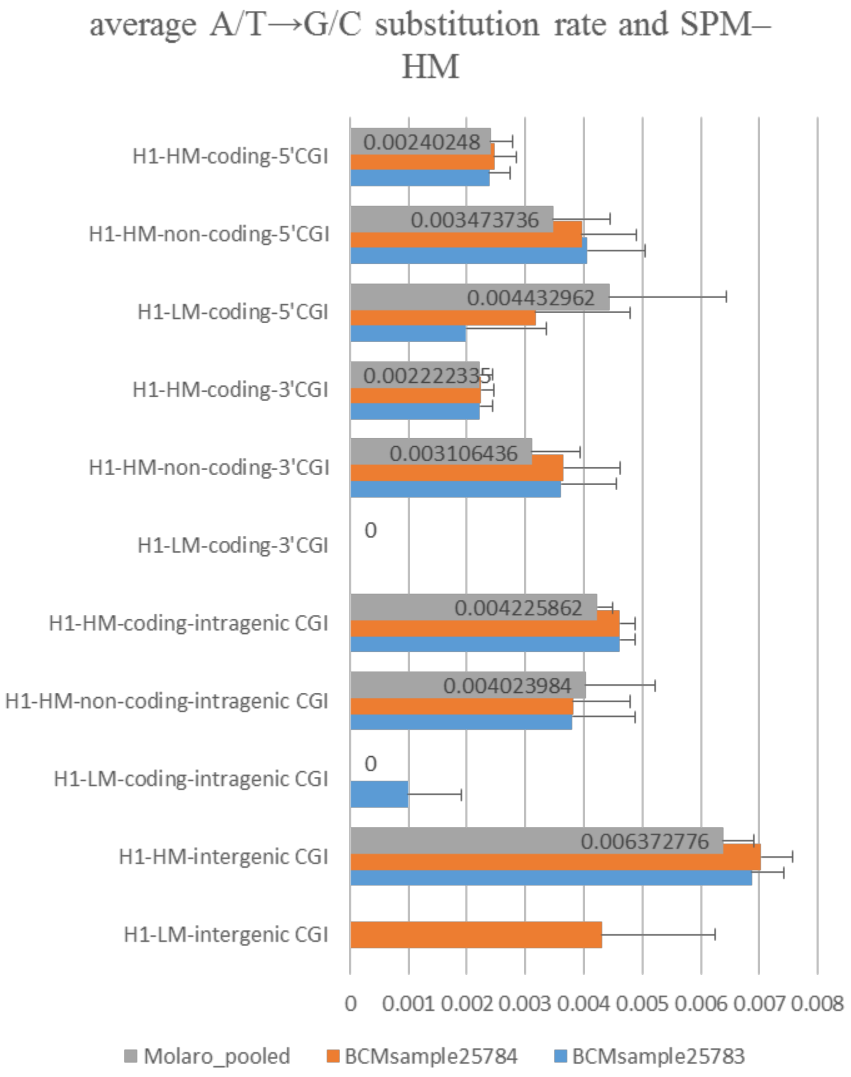

average A/T→G/C substitution rate and SPM–HM
H1-HM-coding-5'CGI
H1-HM-non-coding-5'CGI
H1-LM-coding-5'CGI
H1-HM-coding-3'CGI
H1-HM-non-coding-3'CGI
H1-LM-coding-3'CGI
H1-HM-coding-intragenic CGI
H1-HM-non-coding-intragenic CGI
H1-LM-coding-intragenic CGI
H1-HM-intergenic CGI
H1-LM-intergenic CGI
0.00240248
0.003473736
0.004432962
0.002222335
0.003106436
0
0.004225862
0.004023984
0
0.006372776
0
0.001
0.002
0.003
0.004
0.005
0.006
0.007
0.008
Molaro_pooled
BCMsample25784
BCMsample25783

F

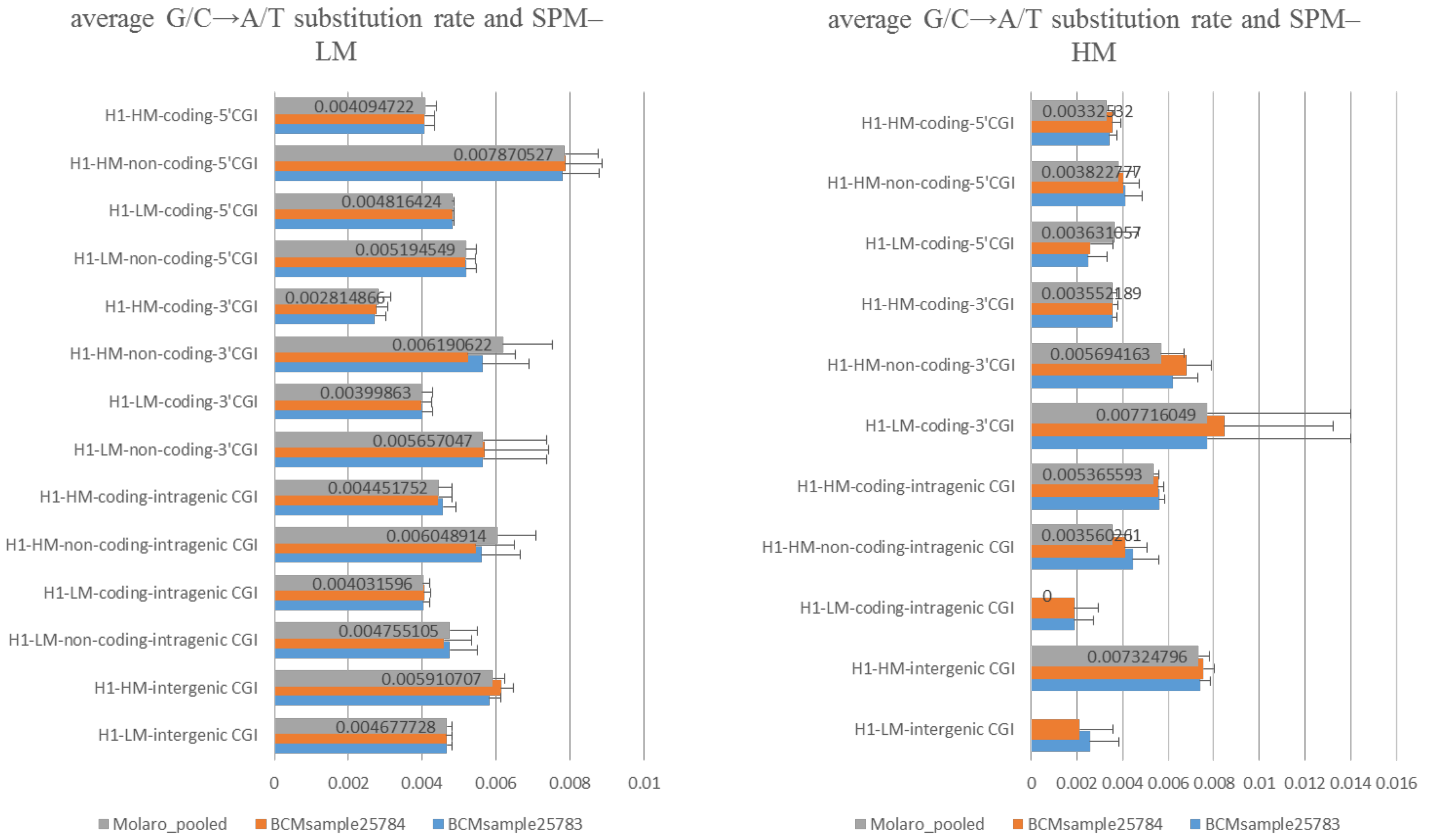
average G/C→A/T substitution rate and SPM–LM
H1-HM-coding-5'CGI
H1-HM-non-coding-5'CGI
H1-LM-coding-5'CGI
H1-LM-non-coding-5'CGI
H1-HM-coding-3'CGI
H1-HM-non-coding-3'CGI
H1-LM-coding-3'CGI
H1-LM-non-coding-3'CGI
H1-HM-coding-intragenic CGI
H1-HM-non-coding-intragenic CGI
H1-LM-coding-intragenic CGI
H1-LM-non-coding-intragenic CGI
H1-HM-intergenic CGI
H1-LM-intergenic CGI
0.004094722
0.007870527
0.004816424
0.005194549
0.002814866
0.006190622
0.00399863
0.005657047
0.004451752
0.006048914
0.004031596
0.004755105
0.005910707
0.004677728
0
0.002
0.004
0.006
0.008
0.01
Molaro_pooled
BCMsample25784
BCMsample25783
average G/C→A/T substitution rate and SPM–HM
H1-HM-coding-5'CGI
H1-HM-non-coding-5'CGI
H1-LM-coding-5'CGI
H1-HM-coding-3'CGI
H1-HM-non-coding-3'CGI
H1-LM-coding-3'CGI
H1-HM-coding-intragenic CGI
H1-HM-non-coding-intragenic CGI
H1-LM-coding-intragenic CGI
H1-HM-intergenic CGI
H1-LM-intergenic CGI
0.003325532
0.003822777
0.003631057
0.003552189
0.005694163
0.007716049
0.005365593
0.003560261
0
0.007324796
0
0.002
0.004
0.006
0.008
0.01
0.012
0.014
0.016
Molaro_pooled
BCMsample25784
BCMsample25783
