# Supplementary material for: Factors to preserve CpG-rich sequences in methylated CpG islands
Source: BMC Genomics. 2015 Feb 28;16(1):144. doi: 10.1186/s12864-015-1286-x (PMC4417305; doi:10.1186/s12864-015-1286-x)
Supplement: Additional file 20: Figure S8. — Comparison of base substitution rates between lowly methylated CGIs with CpG → TpG/CpA < 0.03984 and highly methylated CGIs with CpG → TpG/CpA ≥ 0.03984. Hydroxymethylated CGIs in H1 cells are not included in these CGIs. The details of the figure are the same as those described in the legend to Figure 6. [file 12864_2015_1286_MOESM20_ESM.pdf]

A

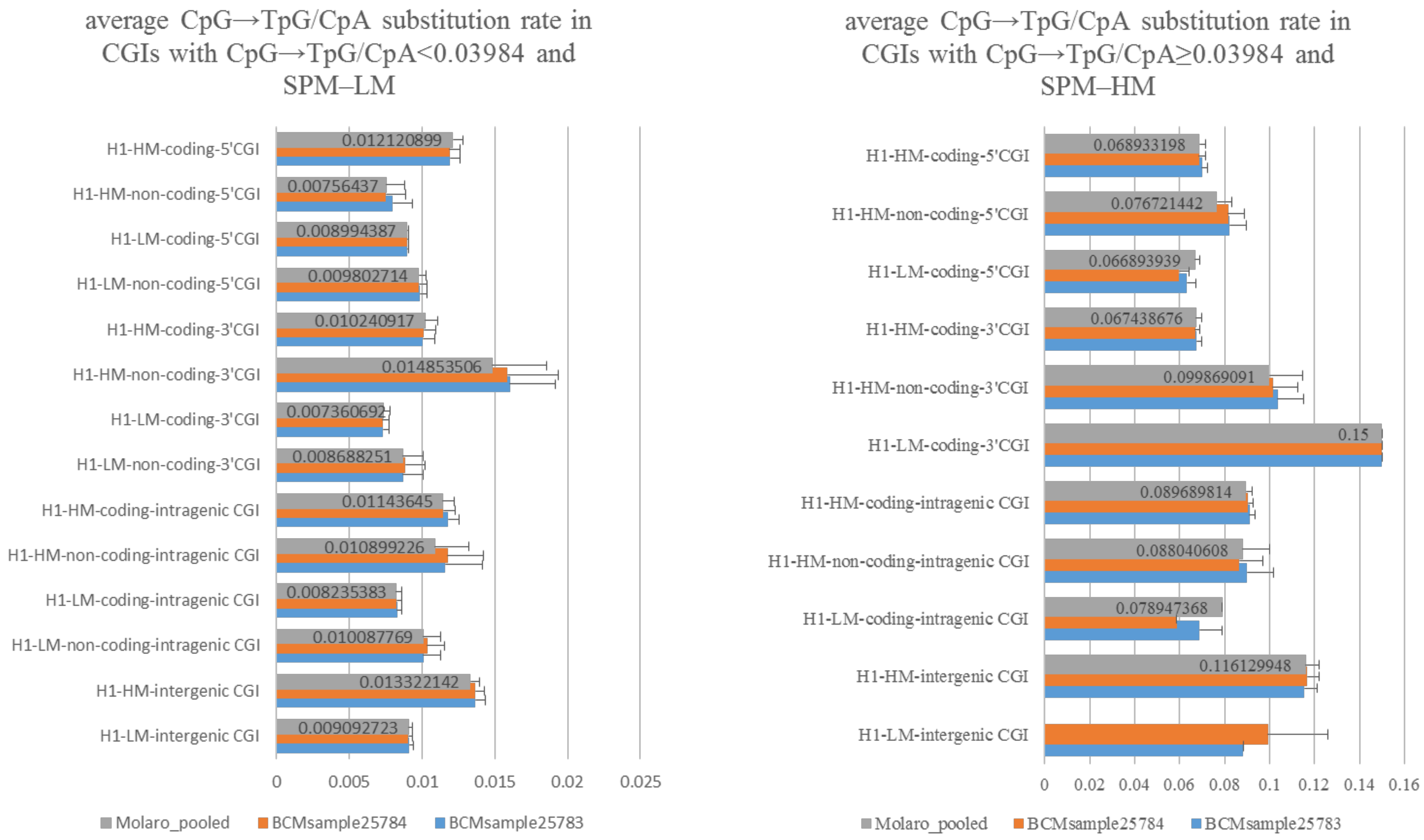
average CpG→TpG/CpA substitution rate in CGIs with CpG→TpG/CpA<0.03984 and SPM–LM
H1-HM-coding-5'CGI
H1-HM-non-coding-5'CGI
H1-LM-coding-5'CGI
H1-LM-non-coding-5'CGI
H1-HM-coding-3'CGI
H1-HM-non-coding-3'CGI
H1-LM-coding-3'CGI
H1-LM-non-coding-3'CGI
H1-HM-coding-intragenic CGI
H1-HM-non-coding-intragenic CGI
H1-LM-coding-intragenic CGI
H1-LM-non-coding-intragenic CGI
H1-HM-intergenic CGI
H1-LM-intergenic CGI
0.012120899
0.00756437
0.008994387
0.009802714
0.010240917
0.014853506
0.007360692
0.008688251
0.01143645
0.010899226
0.008235383
0.010087769
0.013322142
0.009092723
0
0.005
0.01
0.015
0.02
0.025
Molaro_pooled
BCMsample25784
BCMsample25783
average CpG→TpG/CpA substitution rate in CGIs with CpG→TpG/CpA≥0.03984 and SPM–HM
H1-HM-coding-5'CGI
H1-HM-non-coding-5'CGI
H1-LM-coding-5'CGI
H1-HM-coding-3'CGI
H1-HM-non-coding-3'CGI
H1-LM-coding-3'CGI
H1-HM-coding-intragenic CGI
H1-HM-non-coding-intragenic CGI
H1-LM-coding-intragenic CGI
H1-HM-intergenic CGI
H1-LM-intergenic CGI
0.068933198
0.076721442
0.066893939
0.067438676
0.099869091
0.15
0.089689814
0.088040608
0.078947368
0.116129948
0
0.02
0.04
0.06
0.08
0.1
0.12
0.14
0.16
Molaro_pooled
BCMsample25784
BCMsample25783

B

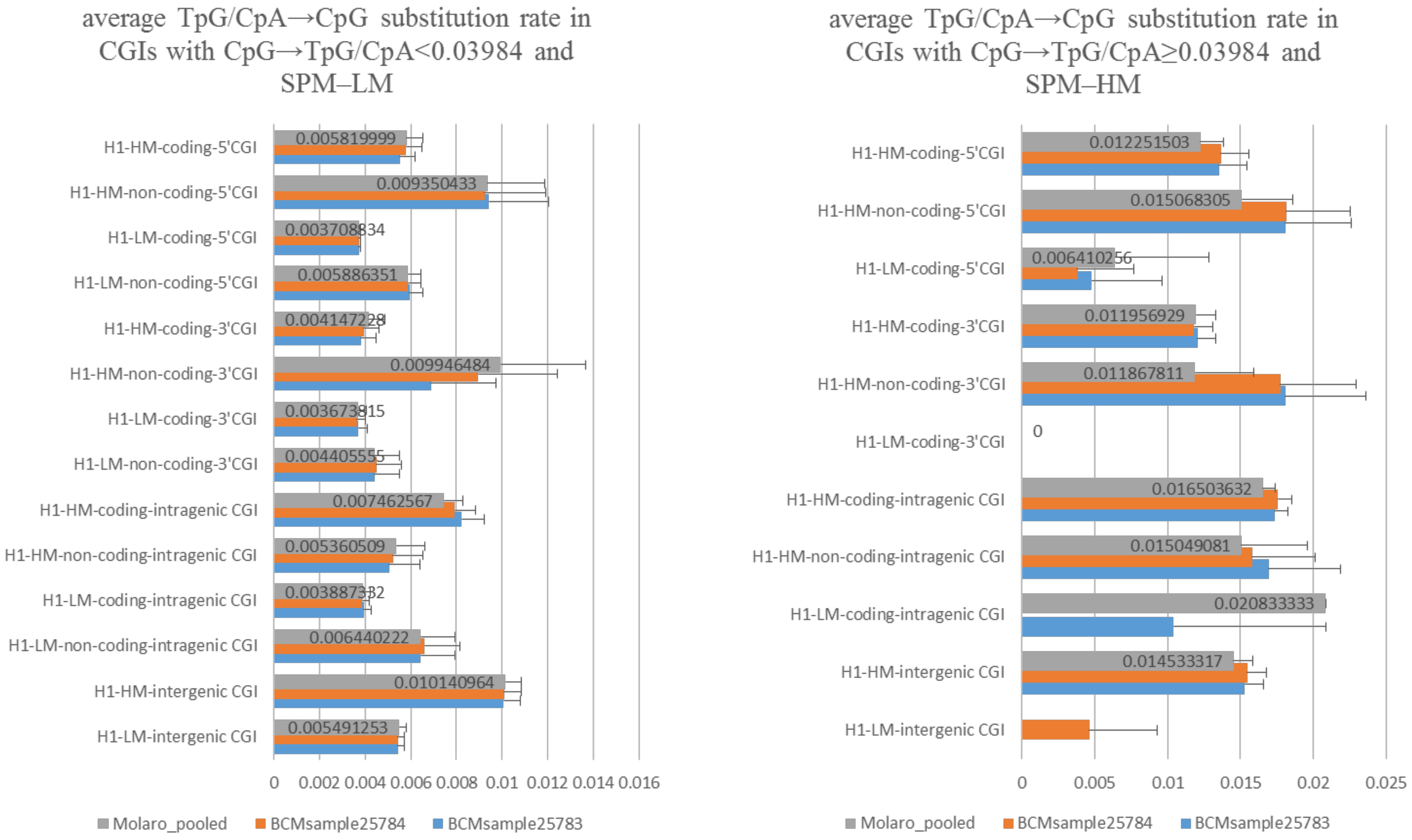
average TpG/CpA→CpG substitution rate in CGIs with CpG→TpG/CpA<0.03984 and SPM–LM
H1-HM-coding-5'CGI
H1-HM-non-coding-5'CGI
H1-LM-coding-5'CGI
H1-LM-non-coding-5'CGI
H1-HM-coding-3'CGI
H1-HM-non-coding-3'CGI
H1-LM-coding-3'CGI
H1-LM-non-coding-3'CGI
H1-HM-coding-intragenic CGI
H1-HM-non-coding-intragenic CGI
H1-LM-coding-intragenic CGI
H1-LM-non-coding-intragenic CGI
H1-HM-intergenic CGI
H1-LM-intergenic CGI
0.005819999
0.009350433
0.003708834
0.005886351
0.004147228
0.009946484
0.003673815
0.004405555
0.007462567
0.005360509
0.003887332
0.006440222
0.010140964
0.005491253
0
0.002
0.004
0.006
0.008
0.01
0.012
0.014
0.016
Molaro_pooled
BCMsample25784
BCMsample25783
average TpG/CpA→CpG substitution rate in CGIs with CpG→TpG/CpA≥0.03984 and SPM–HM
H1-HM-coding-5'CGI
H1-HM-non-coding-5'CGI
H1-LM-coding-5'CGI
H1-HM-coding-3'CGI
H1-HM-non-coding-3'CGI
H1-LM-coding-3'CGI
H1-HM-coding-intragenic CGI
H1-HM-non-coding-intragenic CGI
H1-LM-coding-intragenic CGI
H1-HM-intergenic CGI
H1-LM-intergenic CGI
0.012251503
0.015068305
0.006410256
0.011956929
0.011867811
0
0.016503632
0.015049081
0.020833333
0.014533317
0
0.005
0.01
0.015
0.02
0.025
Molaro_pooled
BCMsample25784
BCMsample25783

C

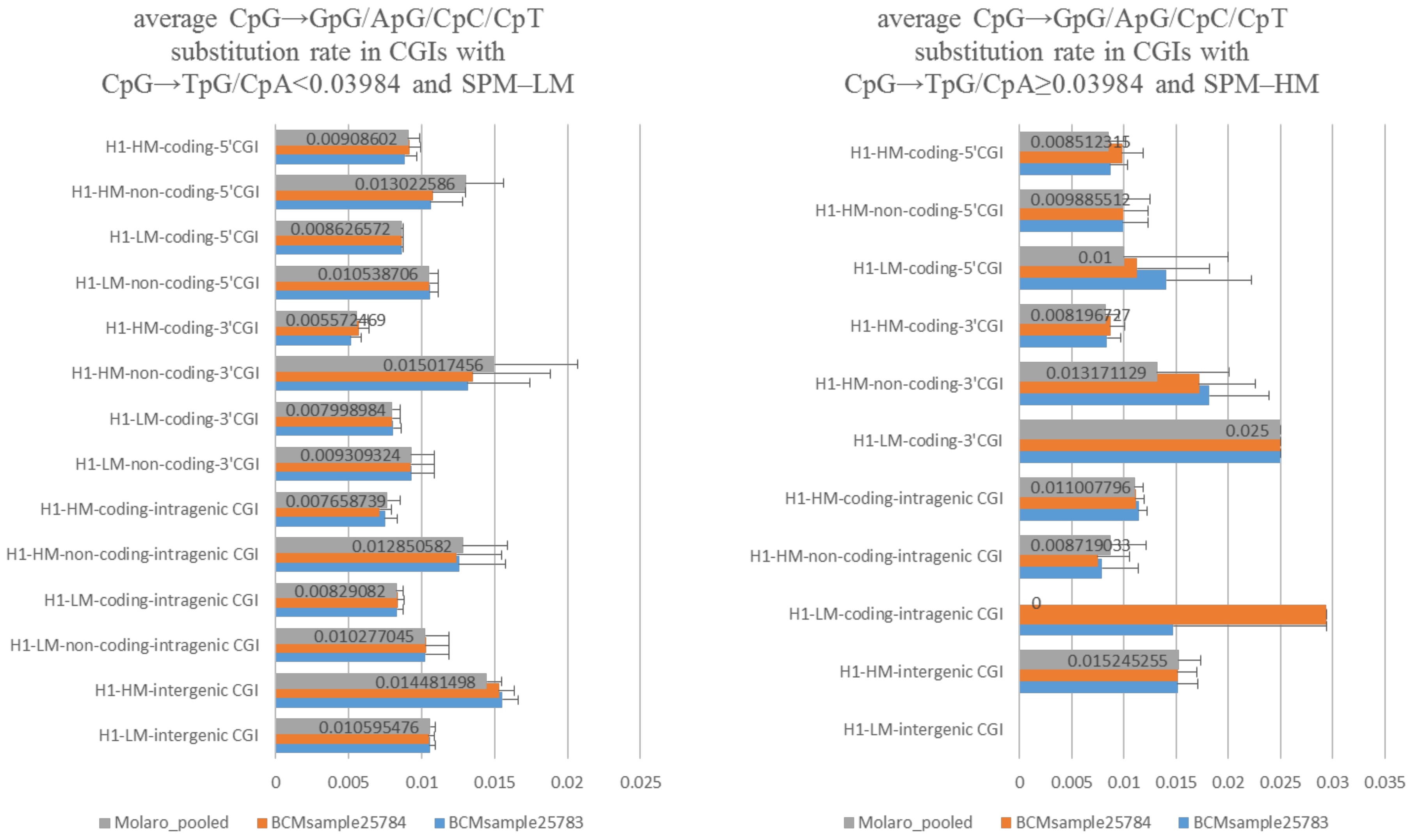

average CpG→GpG/ApG/CpC/CpT substitution rate in CGIs with CpG→TpG/CpA<0.03984 and SPM–LM
H1-HM-coding-5'CGI
H1-HM-non-coding-5'CGI
H1-LM-coding-5'CGI
H1-LM-non-coding-5'CGI
H1-HM-coding-3'CGI
H1-HM-non-coding-3'CGI
H1-LM-coding-3'CGI
H1-LM-non-coding-3'CGI
H1-HM-coding-intragenic CGI
H1-HM-non-coding-intragenic CGI
H1-LM-coding-intragenic CGI
H1-LM-non-coding-intragenic CGI
H1-HM-intergenic CGI
H1-LM-intergenic CGI
0.00908602
0.013022586
0.008626572
0.010538706
0.005572469
0.015017456
0.007998984
0.009309324
0.007658739
0.012850582
0.00829082
0.010277045
0.014481498
0.010595476
0
0.005
0.01
0.015
0.02
0.025
Molaro_pooled
BCMsample25784
BCMsample25783
average CpG→GpG/ApG/CpC/CpT substitution rate in CGIs with CpG→TpG/CpA≥0.03984 and SPM–HM
H1-HM-coding-5'CGI
H1-HM-non-coding-5'CGI
H1-LM-coding-5'CGI
H1-HM-coding-3'CGI
H1-HM-non-coding-3'CGI
H1-LM-coding-3'CGI
H1-HM-coding-intragenic CGI
H1-HM-non-coding-intragenic CGI
H1-LM-coding-intragenic CGI
H1-HM-intergenic CGI
H1-LM-intergenic CGI
0.008512315
0.009885512
0.01
0.008196727
0.013171129
0.025
0.011007796
0.008719033
0
0.015245255
0
0.005
0.01
0.015
0.02
0.025
0.03
0.035
Molaro_pooled
BCMsample25784
BCMsample25783

D

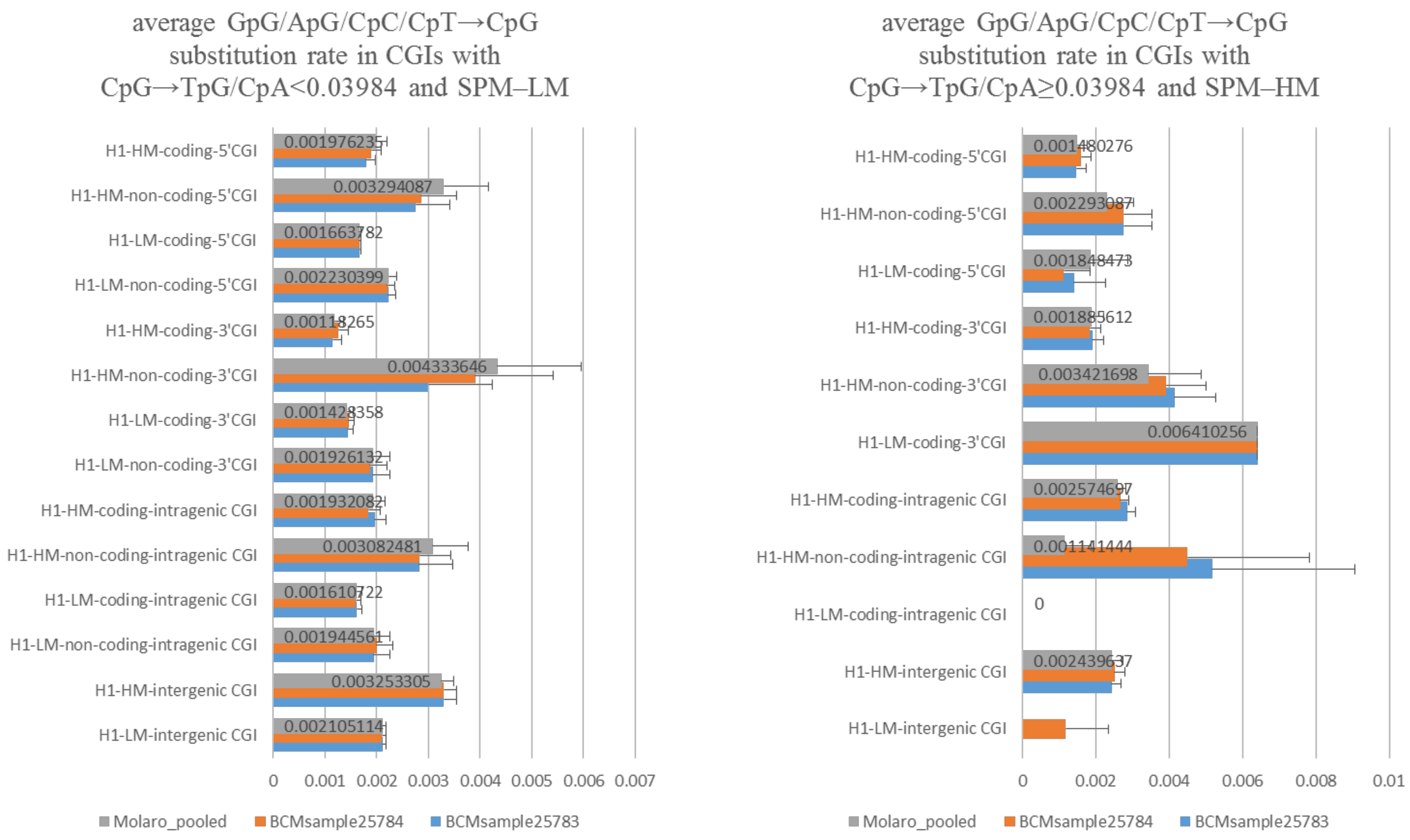
average GpG/ApG/CpC/CpT→CpG substitution rate in CGIs with CpG→TpG/CpA<0.03984 and SPM–LM
H1-HM-coding-5'CGI
H1-HM-non-coding-5'CGI
H1-LM-coding-5'CGI
H1-LM-non-coding-5'CGI
H1-HM-coding-3'CGI
H1-HM-non-coding-3'CGI
H1-LM-coding-3'CGI
H1-LM-non-coding-3'CGI
H1-HM-coding-intragenic CGI
H1-HM-non-coding-intragenic CGI
H1-LM-coding-intragenic CGI
H1-LM-non-coding-intragenic CGI
H1-HM-intergenic CGI
H1-LM-intergenic CGI
0.001976235
0.003294087
0.001663782
0.002230399
0.001118265
0.004333646
0.001428358
0.001926132
0.001932082
0.003082481
0.001610722
0.001944561
0.003253305
0.002105114
0
0.001
0.002
0.003
0.004
0.005
0.006
0.007
Molaro_pooled
BCMsample25784
BCMsample25783
average GpG/ApG/CpC/CpT→CpG substitution rate in CGIs with CpG→TpG/CpA≥0.03984 and SPM–HM
H1-HM-coding-5'CGI
H1-HM-non-coding-5'CGI
H1-LM-coding-5'CGI
H1-HM-coding-3'CGI
H1-HM-non-coding-3'CGI
H1-LM-coding-3'CGI
H1-HM-coding-intragenic CGI
H1-HM-non-coding-intragenic CGI
H1-LM-coding-intragenic CGI
H1-HM-intergenic CGI
H1-LM-intergenic CGI
0.001480276
0.002293087
0.001848473
0.001885612
0.003421698
0.006410256
0.002574697
0.001141444
0
0.002439637
0
0.002
0.004
0.006
0.008
0.01
Molaro_pooled
BCMsample25784
BCMsample25783

E

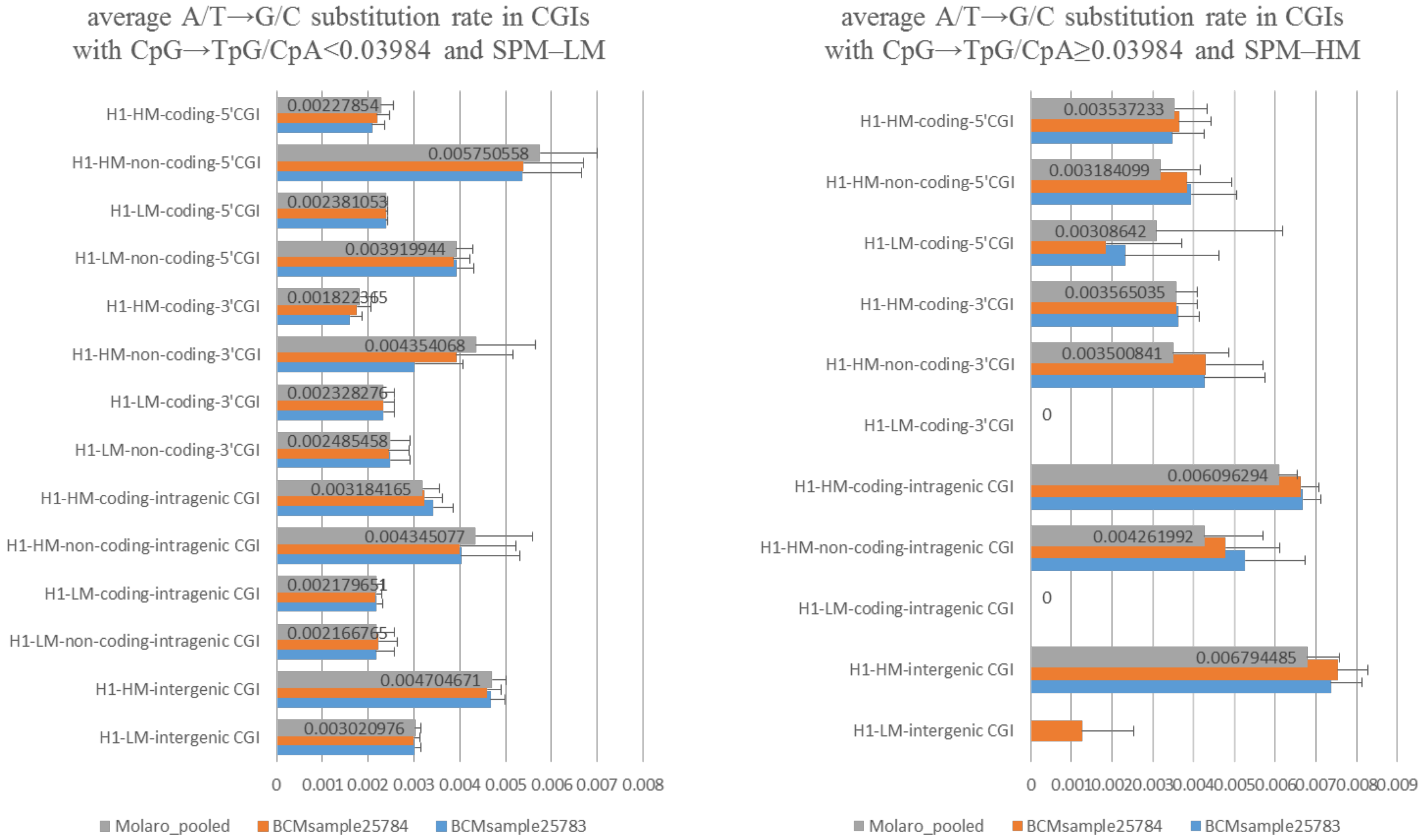
average A/T→G/C substitution rate in CGIs
with CpG→TpG/CpA<0.03984 and SPM–LM
H1-HM-coding-5'CGI
H1-HM-non-coding-5'CGI
H1-LM-coding-5'CGI
H1-LM-non-coding-5'CGI
H1-HM-coding-3'CGI
H1-HM-non-coding-3'CGI
H1-LM-coding-3'CGI
H1-LM-non-coding-3'CGI
H1-HM-coding-intragenic CGI
H1-HM-non-coding-intragenic CGI
H1-LM-coding-intragenic CGI
H1-LM-non-coding-intragenic CGI
H1-HM-intergenic CGI
H1-LM-intergenic CGI
0.00227854
0.005750558
0.002381053
0.003919944
0.001822365
0.004354068
0.002328276
0.002485458
0.003184165
0.004345077
0.002179651
0.002166765
0.004704671
0.003020976
0 0.001 0.002 0.003 0.004 0.005 0.006 0.007 0.008
Molaro_pooled
BCMsample25784
BCMsample25783
average A/T→G/C substitution rate in CGIs
with CpG→TpG/CpA≥0.03984 and SPM–HM
H1-HM-coding-5'CGI
H1-HM-non-coding-5'CGI
H1-LM-coding-5'CGI
H1-HM-coding-3'CGI
H1-HM-non-coding-3'CGI
H1-LM-coding-3'CGI
H1-HM-coding-intragenic CGI
H1-HM-non-coding-intragenic CGI
H1-LM-coding-intragenic CGI
H1-HM-intergenic CGI
H1-LM-intergenic CGI
0.003537233
0.003184099
0.00308642
0.003565035
0.003500841
0
0.006096294
0.004261992
0
0.006794485
0 0.0010.0020.0030.0040.0050.0060.0070.0080.009
Molaro_pooled
BCMsample25784
BCMsample25783

F

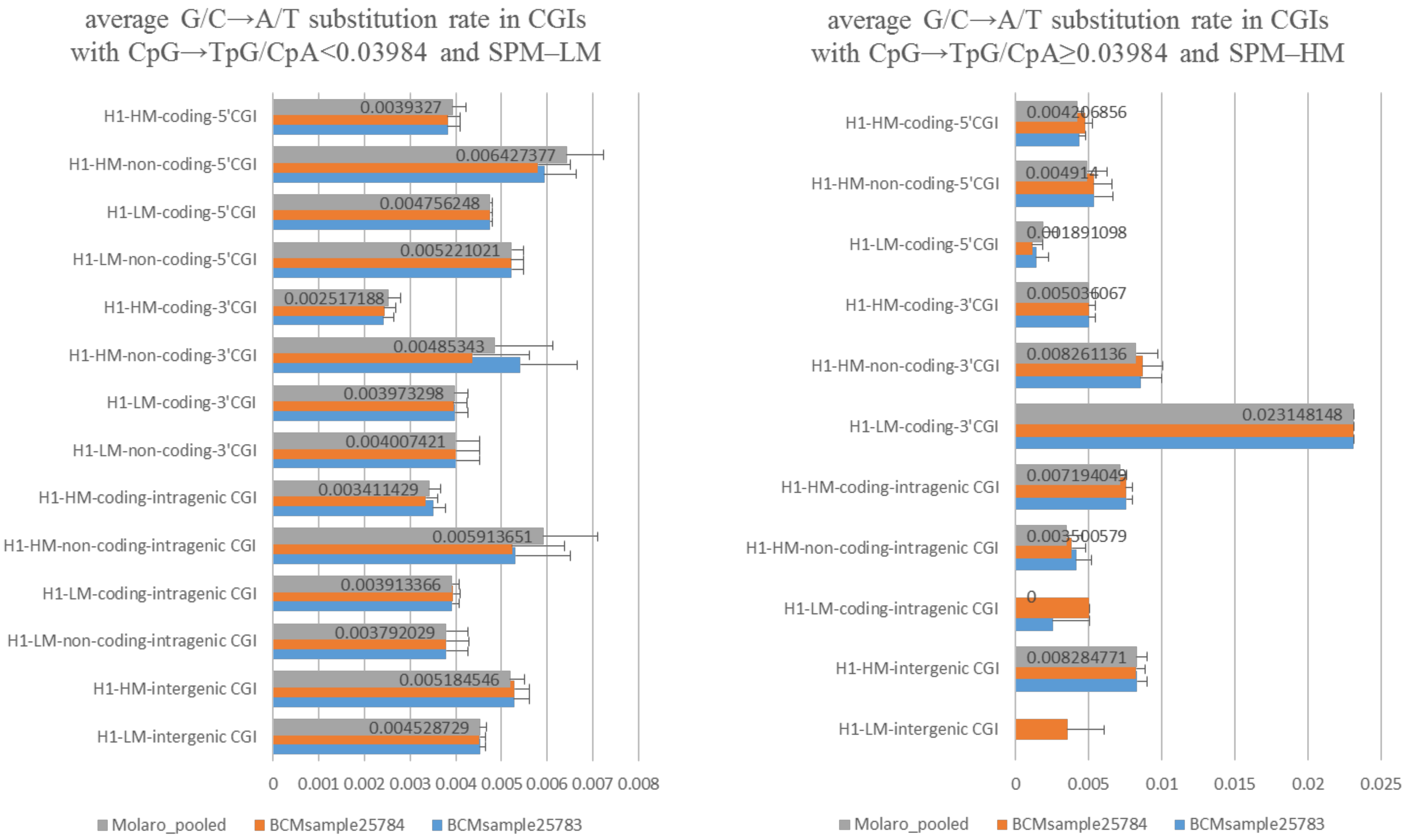

average G/C→A/T substitution rate in CGIs with CpG→TpG/CpA<0.03984 and SPM–LM
H1-HM-coding-5'CGI
H1-HM-non-coding-5'CGI
H1-LM-coding-5'CGI
H1-LM-non-coding-5'CGI
H1-HM-coding-3'CGI
H1-HM-non-coding-3'CGI
H1-LM-coding-3'CGI
H1-LM-non-coding-3'CGI
H1-HM-coding-intragenic CGI
H1-HM-non-coding-intragenic CGI
H1-LM-coding-intragenic CGI
H1-LM-non-coding-intragenic CGI
H1-HM-intergenic CGI
H1-LM-intergenic CGI
0.0039327
0.006427377
0.004756248
0.005221021
0.002517188
0.00485343
0.003973298
0.004007421
0.003411429
0.005913651
0.003913366
0.003792029
0.005184546
0.004528729
0 0.001 0.002 0.003 0.004 0.005 0.006 0.007 0.008
Molaro_pooled BCMsample25784 BCMsample25783
average G/C→A/T substitution rate in CGIs with CpG→TpG/CpA≥0.03984 and SPM–HM
H1-HM-coding-5'CGI
H1-HM-non-coding-5'CGI
H1-LM-coding-5'CGI
H1-HM-coding-3'CGI
H1-HM-non-coding-3'CGI
H1-LM-coding-3'CGI
H1-HM-coding-intragenic CGI
H1-HM-non-coding-intragenic CGI
H1-LM-coding-intragenic CGI
H1-HM-intergenic CGI
H1-LM-intergenic CGI
0.004206856
0.004914
0.001891098
0.005036067
0.008261136
0.023148148
0.007194049
0.003500579
0
0.008284771
0 0.005 0.01 0.015 0.02 0.025
Molaro_pooled BCMsample25784 BCMsample25783
